# Supplementary material for: Using electronic health records to quantify and stratify the severity of type 2 diabetes in primary care in England: rationale and cohort study design
Source: BMJ Open. 2018 Jun 30;8(6):e020926. doi: 10.1136/bmjopen-2017-020926 (PMC6042592; doi:10.1136/bmjopen-2017-020926)
Supplement: Supplementary data [file bmjopen-2017-020926supp001.pdf]

Supplementary data

Table S1 Inclusion medical codes for type 2 diabetes

	Medical code	Read term
**1.**	506	Non-insulin dependent diabetes mellitus
**2.**	758	Type 2 diabetes mellitus
**3.**	1407	Insulin treated Type 2 diabetes mellitus
**4.**	4513	Non-insulin dependent diabetes mellitus
**5.**	5884	NIDDM - Non-insulin dependent diabetes mellitus
**6.**	8403	Non-insulin dependent diabetes mellitus - poor control
**7.**	12640	Type 2 diabetes mellitus with nephropathy
**8.**	12736	Type 2 diabetes mellitus with gangrene
**9.**	17262	Non-insulin-dependent diabetes mellitus with retinopathy
**10.**	17859	Type 2 diabetes mellitus
**11.**	18143	Type II diabetes mellitus with arthropathy
**12.**	18209	Type 2 diabetes mellitus with renal complications
**13.**	18219	Type II diabetes mellitus
**14.**	18264	Insulin treated Type II diabetes mellitus
**15.**	18278	Insulin treated Type 2 diabetes mellitus
**16.**	18390	Type 2 diabetes mellitus with persistent microalbuminuria
**17.**	18425	Type 2 diabetes mellitus with polyneuropathy
**18.**	18496	Type 2 diabetes mellitus with retinopathy
**19.**	18777	Type 2 diabetes mellitus with renal complications
**20.**	22884	Type II diabetes mellitus
**21.**	24458	Type II diabetes mellitus - poor control
**22.**	24693	Non-insulin dependent diabetes mellitus with arthropathy
**23.**	24836	Type 2 diabetes mellitus with nephropathy
**24.**	25041	Dietary advice for type II diabetes
**25.**	25591	Type 2 diabetes mellitus with exudative maculopathy
**26.**	25627	Type 2 diabetes mellitus - poor control
**27.**	26054	Type 2 diabetes mellitus with persistent proteinuria
**28.**	29979	Non-insulin-dependent diabetes mellitus without complication
**29.**	32627	Type 2 diabetes mellitus with ketoacidosis
**30.**	34268	Type 2 diabetes mellitus with neurological complications
**31.**	34450	Hyperosmolar non-ketotic state in type 2 diabetes mellitus
**32.**	34912	Non-insulin dependent diabetes mellitus with ulcer
**33.**	35385	Type 2 diabetes mellitus with neuropathic arthropathy
**34.**	36633	Hyperosmolar non-ketotic state in type 2 diabetes mellitus
**35.**	36695	Diabetes mellitus autosomal dominant type 2
**36.**	37648	Insulin treated non-insulin dependent diabetes mellitus
**37.**	37806	Type 2 diabetes mellitus with peripheral angiopathy
**38.**	40401	Non-insulin dependent diabetes mellitus with gangrene
**39.**	42762	Type 2 diabetes mellitus with retinopathy
**40.**	43227	Type II diabetes mellitus with multiple complications
**41.**	43785	Non-insulin dependent diabetes mellitus with hypoglyca coma
**42.**	44779	Type 2 diabetes mellitus with diabetic cataract
**43.**	44982	Type 2 diabetes mellitus with diabetic cataract
**44.**	45467	Non-insulin dependent diabetes mellitus with polyneuropathy
**45.**	45913	Type 2 diabetes mellitus - poor control
**46.**	45919	Type 2 diabetes mellitus with neurological complications

	Medical code	Read term
**47.**	46150	Type 2 diabetes mellitus with gangrene
**48.**	46917	Type 2 diabetes mellitus with hypoglycaemic coma
**49.**	47315	Type II diabetes mellitus - poor control
**50.**	47321	Type 2 diabetes mellitus with ophthalmic complications
**51.**	47409	Type II diabetes mellitus with polyneuropathy
**52.**	47816	Type II diabetes mellitus with neuropathic arthropathy
**53.**	47954	Type 2 diabetes mellitus without complication
**54.**	48192	Type II diabetes mellitus with diabetic cataract
**55.**	49074	Type 2 diabetes mellitus with ulcer
**56.**	49655	Type II diabetes mellitus with retinopathy
**57.**	49869	Type 2 diabetes mellitus with arthropathy
**58.**	50225	Type II diabetes mellitus with renal complications
**59.**	50429	Non-insulin-dependent diabetes mellitus with ophthalm comps
**60.**	50527	Type II diabetes mellitus with polyneuropathy
**61.**	50609	Pre-existing diabetes mellitus, non-insulin-dependent
**62.**	50813	Type II diabetes mellitus with mononeuropathy
**63.**	51756	Type 2 diabetes mellitus with ketoacidotic coma
**64.**	52303	Non-insulin-dependent diabetes mellitus with renal comps
**65.**	53392	Type II diabetes mellitus without complication
**66.**	54899	Type II diabetes mellitus with peripheral angiopathy
**67.**	55075	Type II diabetes mellitus with ulcer
**68.**	55842	Non-insulin-dependent diabetes mellitus with neuro comps
**69.**	56268	Type II diabetes mellitus with hypoglycaemic coma
**70.**	56803	NIDDM with peripheral circulatory disorder
**71.**	57278	Type II diabetes mellitus with renal complications
**72.**	58604	Type II diabetes mellitus with retinopathy
**73.**	59253	Type 2 diabetes mellitus with arthropathy
**74.**	59365	Non-insulin dependent diabetes mellitus with nephropathy
**75.**	59725	Type II diabetes mellitus with ophthalmic complications
**76.**	60699	Type 2 diabetes mellitus with peripheral angiopathy
**77.**	60796	Type II diabetes mellitus with persistent proteinuria
**78.**	61071	Type 2 diabetes mellitus with hypoglycaemic coma
**79.**	62107	Type II diabetes mellitus with gangrene
**80.**	62146	Non-insulin-dependent diabetes mellitus with multiple comps
**81.**	62674	Type 2 diabetes mellitus with mononeuropathy
**82.**	63690	Type 2 diabetes mellitus with gastroparesis
**83.**	64571	Type II diabetes mellitus with nephropathy
**84.**	64668	Insulin treated Type II diabetes mellitus
**85.**	65267	Type 2 diabetes mellitus with multiple complications
**86.**	65704	Type 2 diabetes mellitus with ulcer
**87.**	66965	Type 2 diabetes mellitus with neuropathic arthropathy
**88.**	67905	Type II diabetes mellitus with neurological complications
**89.**	69278	Non-insulin depend diabetes mellitus with diabetic cataract
**90.**	70316	Type 2 diabetes mellitus with ophthalmic complications
**91.**	72320	Non-insulin dependent diabetes mellitus with mononeuropathy
**92.**	83532	Diabetes type 2 review
**93.**	85991	Type II diabetes mellitus with persistent microalbuminuria
**94.**	91646	Type II diabetes mellitus with ulcer
**95.**	93727	Type II diabetes mellitus with diabetic cataract
**96.**	95351	Type II diabetes mellitus with mononeuropathy
**97.**	98616	Type II diabetes mellitus with neurological complications
**98.**	98723	Type II diabetes mellitus with hypoglycaemic coma
**99.**	100964	Type II diabetes mellitus with ophthalmic complications

	Medical code	Read term
**100.**	101801	Type II diabetic dietary review
**101.**	102201	Type II diabetes mellitus with nephropathy
**102.**	102611	Type 2 diabetic dietary review
**103.**	103902	Type II diabetes mellitus with arthropathy
**104.**	104323	Type II diabetes mellitus with gangrene
**105.**	104639	Type II diabetes mellitus with peripheral angiopathy
**106.**	105784	Type 2 diabetes mellitus without complication
**107.**	106061	Type II diabetes mellitus with ketoacidotic coma
**108.**	106528	Type II diabetes mellitus with ketoacidosis
**109.**	107701	Hyperosmolar non-ketotic state in type II diabetes mellitus
**110.**	107824	Type II diabetes mellitus in remission
**111.**	108005	Type 2 diabetes mellitus with multiple complications
**112.**	109103	Type II diabetes mellitus without complication
**113.**	109197	Type II diabetes mellitus with neuropathic arthropathy
**114.**	109865	Type 2 diabetes mellitus with polyneuropathy
**115.**	111798	Type II diabetes mellitus with exudative maculopathy

Table S2 Exclusion type 1 diabetes codes

	Medical code	Read term
**1.**	1038	Insulin dependent diabetes mellitus
**2.**	1549	Type 1 diabetes mellitus
**3.**	1647	Insulin dependent diabetes mellitus
**4.**	6509	Insulin dependent diabetes mellitus with retinopathy
**5.**	6791	Insulin dependent diabetes mellitus - poor control
**6.**	10418	Type 1 diabetes mellitus with nephropathy
**7.**	10692	Type 1 diabetes mellitus with ketoacidosis
**8.**	12455	Type I diabetes mellitus
**9.**	17545	Type I diabetes mellitus with diabetic cataract
**10.**	17858	Type 1 diabetes mellitus
**11.**	18230	Type 1 diabetes mellitus with neuropathic arthropathy
**12.**	18387	Type 1 diabetes mellitus with retinopathy
**13.**	18505	IDDM-Insulin dependent diabetes mellitus
**14.**	18642	Type 1 diabetes mellitus with arthropathy
**15.**	18683	Type 1 diabetes mellitus with ulcer
**16.**	21983	Type 1 diabetes mellitus with renal complications
**17.**	22871	Type 1 diabetes mellitus with exudative maculopathy
**18.**	24423	Type I diabetes mellitus
**19.**	24490	Diabetes mellitus, juvenile type, no mention of complication
**20.**	24694	Insulin dependent diabetes mellitus with mononeuropathy
**21.**	26855	Unstable insulin dependent diabetes mellitus
**22.**	30294	Type 1 diabetes mellitus with persistent microalbuminuria
**23.**	30323	Type 1 diabetes mellitus with persistent proteinuria
**24.**	31310	Insulin dependent diabetes maturity onset
**25.**	32359	Perceived control of insulin-dependent diabetes
**26.**	35288	Type 1 diabetes mellitus - poor control
**27.**	38161	Type I diabetes mellitus with retinopathy
**28.**	39070	Type 1 diabetes mellitus with hypoglycaemic coma
**29.**	39809	Insulin dependent diab mell with neuropathic arthropathy
**30.**	40023	Diabetes mellitus, juvenile type, with hyperosmolar coma
**31.**	40682	Type 1 diabetes mellitus maturity onset
**32.**	40837	Type 1 diabetes mellitus with ketoacidotic coma
**33.**	41049	Type 1 diabetes mellitus with retinopathy
**34.**	41716	Insulin dependent diabetes mellitus with polyneuropathy
**35.**	42567	Diabetes mellitus, juvenile type, with ketoacidotic coma
**36.**	42729	Type I diabetes mellitus with hypoglycaemic coma
**37.**	42831	Type 1 diabetes mellitus with neurological complications
**38.**	43921	Unstable type 1 diabetes mellitus
**39.**	44260	Insulin dependent diabetes mellitus with diabetic cataract
**40.**	44440	Insulin dependent diabetes mellitus with hypoglycaemic coma
**41.**	44443	Insulin dependent diabetes mellitus with ulcer
**42.**	45276	Insulin dependent diabetes mellitus with multiple complicat
**43.**	45914	Type 1 diabetes mellitus - poor control
**44.**	46301	Type 1 diabetes mellitus with polyneuropathy
**45.**	46850	Type I diabetes mellitus - poor control
**46.**	46963	Insulin-dependent diabetes mellitus with renal complications
**47.**	47582	Type 1 diabetes mellitus with renal complications

	Medical code	Read term
**48.**	47649	Type 1 diabetes mellitus with ophthalmic complications
**49.**	47650	Type 1 diabetes mellitus with multiple complications
**50.**	49146	Type I diabetes mellitus with neurological complications
**51.**	49276	Insulin-dependent diabetes mellitus with ophthalmic comps
**52.**	49554	Type 1 diabetes mellitus with diabetic cataract
**53.**	49949	Unstable type I diabetes mellitus
**54.**	50960	Pre-existing diabetes mellitus, insulin-dependent
**55.**	51261	Insulin dependent diabetes mellitus
**56.**	51957	Type I diabetes mellitus with ulcer
**57.**	52104	Insulin dependent diabetes mellitus with multiple complicatn
**58.**	52283	Insulin-dependent diabetes mellitus with neurological comps
**59.**	53200	Diabetes mellitus, juvenile type, with ketoacidosis
**60.**	54008	Type 1 diabetes mellitus with neuropathic arthropathy
**61.**	54600	Unstable insulin dependent diabetes mellitus
**62.**	55239	Type 1 diabetes mellitus with gastroparesis
**63.**	56448	Insulin-dependent diabetes without complication
**64.**	57621	Insulin dependent diabetes mellitus with nephropathy
**65.**	60107	Unstable type I diabetes mellitus
**66.**	60208	Type I diabetes mellitus with neuropathic arthropathy
**67.**	60499	Insulin dependent diabetes mellitus with gangrene
**68.**	61344	Type I diabetes mellitus with renal complications
**69.**	61829	Type 1 diabetes mellitus with neurological complications
**70.**	62209	Type I diabetes mellitus with ketoacidosis
**71.**	62352	Type I diabetes mellitus with arthropathy
**72.**	62613	Type I diabetes mellitus without complication
**73.**	63017	Type I diabetes mellitus maturity onset
**74.**	64446	Insulin dependent diab mell with peripheral angiopathy
**75.**	65616	Insulin dependent diabetes mellitus with arthropathy
**76.**	66145	Type I diabetes mellitus with ketoacidotic coma
**77.**	66872	Type I diabetes mellitus with nephropathy
**78.**	68105	Type 1 diabetes mellitus with mononeuropathy
**79.**	68390	Type 1 diabetes mellitus with ulcer
**80.**	68792	Diabetes mellitus, juvenile type, + unspecified complication
**81.**	69043	Dietary advice for type I diabetes
**82.**	69676	Type 1 diabetes mellitus without complication
**83.**	69748	Diabetes mellitus, juvenile type, + ophthalmic manifestation
**84.**	69993	Type 1 diabetes mellitus with gangrene
**85.**	70766	Type 1 diabetes mellitus with hypoglycaemic coma
**86.**	72702	Insulin dependent diabetes mellitus - poor control
**87.**	85660	Diabetes type 1 review
**88.**	91942	Type I diabetes mellitus with multiple complications
**89.**	91943	Type I diabetes mellitus with polyneuropathy
**90.**	93468	Type 1 diabetes mellitus with peripheral angiopathy
**91.**	93875	Insulin dependent diabetes mellitus with retinopathy
**92.**	93878	Type I diabetes mellitus with ulcer
**93.**	93922	Diabetes mellitus, juvenile type, with renal manifestation
**94.**	95343	Type I diabetes mellitus with retinopathy
**95.**	95992	Type I diabetes mellitus without complication
**96.**	96235	Type I diabetes mellitus maturity onset
**97.**	97446	Type 1 diabetes mellitus maturity onset
**98.**	97474	Unstable type 1 diabetes mellitus
**99.**	97849	Insulin dependent diabetes maturity onset
**100.**	97894	Type I diabetes mellitus with exudative maculopathy

	Medical code	Read term
**101.**	98071	Insulin-dependent diabetes mellitus with ophthalmic comps
**102.**	98392	Maturity onset diabetes in youth type 1
**103.**	98704	Insulin dependent diabetes mellitus with ulcer
**104.**	99231	Type I diabetes mellitus with mononeuropathy
**105.**	99311	Type I diabetes mellitus with ophthalmic complications
**106.**	99716	Insulin dependent diabetes mellitus with hypoglycaemic coma
**107.**	99719	Insulin-dependent diabetes without complication
**108.**	100770	Insulin dependent diabetes mellitus with diabetic cataract
**109.**	101311	Insulin dependent diabetes mellitus with polyneuropathy
**110.**	101735	Insulin-dependent diabetes mellitus with neurological comps
**111.**	102112	Type I diabetes mellitus with gangrene
**112.**	102163	Insulin dependent diabetes mellitus with nephropathy
**113.**	102620	Type I diabetes mellitus with persistent microalbuminuria
**114.**	102704	Type I diabetic dietary review
**115.**	102740	Type 1 diabetes mellitus with ophthalmic complications
**116.**	102946	Insulin-dependent diabetes mellitus with renal complications
**117.**	104453	Type 1 diabetic dietary review
**118.**	105337	Type I diabetes mellitus - poor control
**119.**	108007	Type I diabetes mellitus with multiple complications
**120.**	108360	Type I diabetes mellitus in remission
**121.**	108724	Type I diabetes mellitus with gastroparesis
**122.**	109051	Insulin dependent diabetes mellitus with gangrene
**123.**	109628	Type 1 diabetes mellitus in remission
**124.**	109837	Type I diabetes mellitus with renal complications
**125.**	109878	Diet advice for insulin-dependent diabetes
**126.**	110400	Type 1 diabetes mellitus with diabetic cataract
**127.**	111106	Type 1 diabetes mellitus without complication

Figure S1 Severity hierarchy of cardiovascular disease domains (cardiac muscle- myocardium and pericardium).

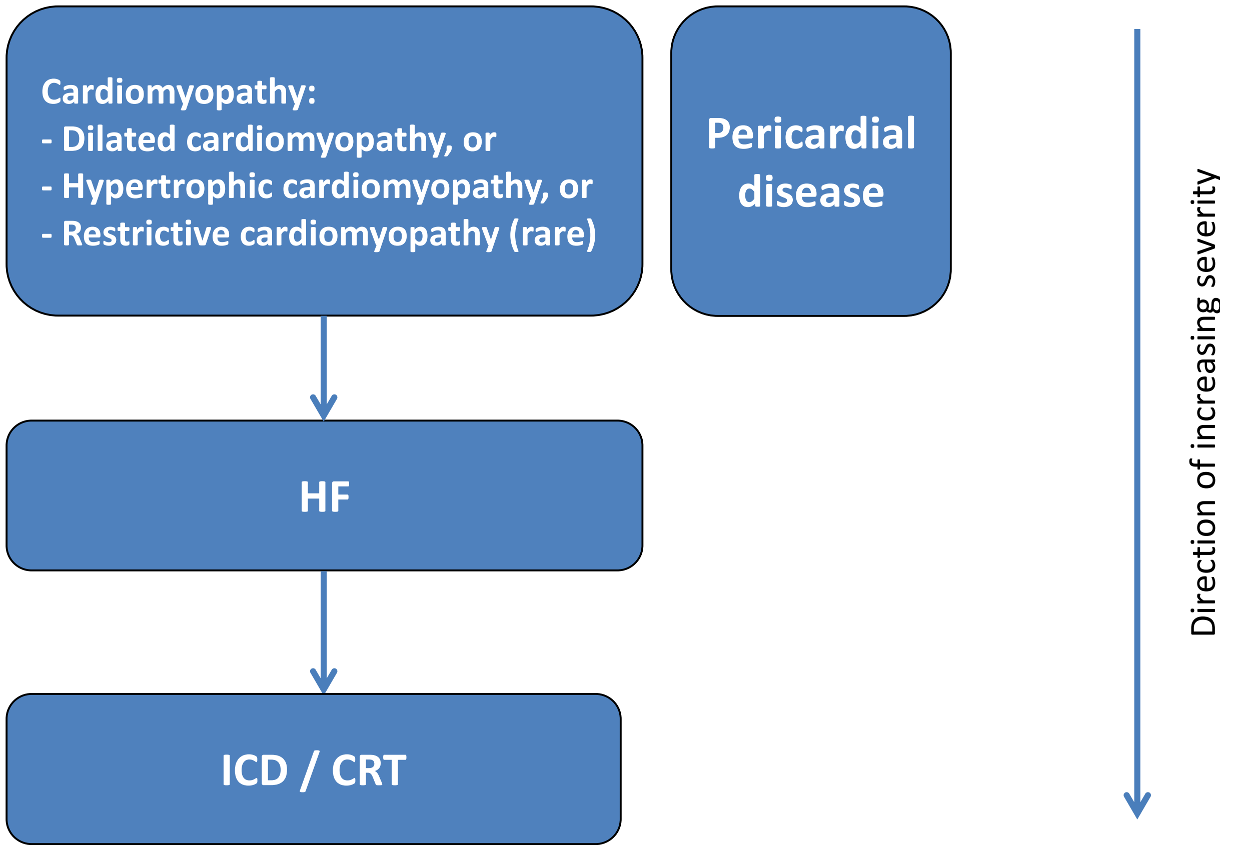

Figure S2 Severity hierarchy of cardiovascular disease domains (Heart valve diseases)

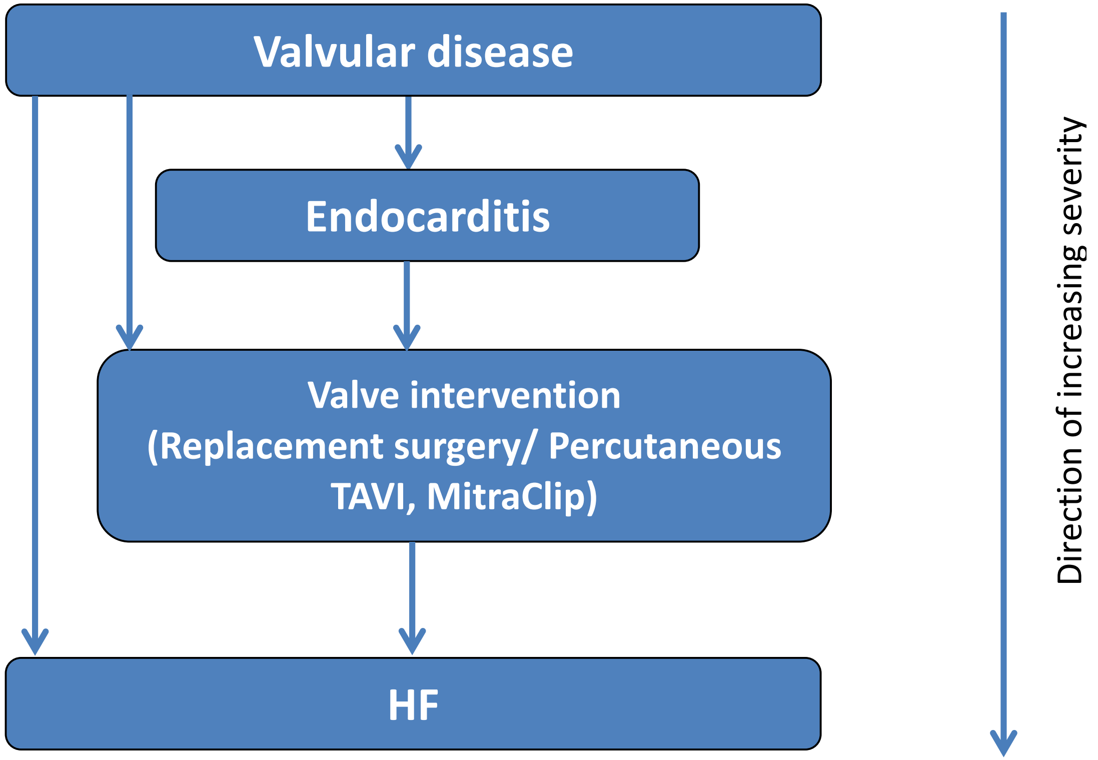

Table S3 Factors used in previous studies to define severity of diabetes

Study ID	Factors used to define diabetes severity / Outcome(s)	Severity scale/ levels	Validation study
**Gini et al. (2016)[(1)]**	• Indication for insulin therapy<br>• The presence of diabetes-related complications<br><br>**Outcomes:**<br>Assessing the positive predictive value (PPV) for the presence/absence of type 2 diabetes against the GP's response to the questionnaire and measure the agreement on the severity level.	Four levels of T2DM severity:<br>• **Level 1**: Clinical definition of the disease, no indication for Insulin therapy and no complications listed in Level 3.<br>• **Level 2**: as in Level 1 except insulin is indicated.<br>• **Level 3**: as in Level 1 except with one of the following complications: (1) arterial stenosis, angina, MI, TIA, ischaemic stroke, intermittent claudication, diabetic foot ulcer, lower limb amputation, (2) retinopathy, (3) incipient or overt diabetic nephropathy/ dialysis.<br>• **Level 4**: As in Level 3 except insulin is indicated	A random sample of identified cases was validated by interviewing their self-selected general practitioners (GPs).
**Grootenhuis et al. (1994)[(2)]**	Type 2 diabetes symptom severity items categorised over six clinical dimensions:<br>• Hyperglycaemic<br>• Hypoglycaemic<br>• Psychological<br>• Cardiovascular<br>• Neuropathic<br>• Ophthalmological<br><br>**Outcomes:**<br>To measure differences in symptom severity between patients and detect changes over time within patients.	A 34-item patient-derived checklist used as a measure for type 2 diabetes symptom severity.	The face and content validity of severity items and dimension structures were based on literature and experiences of diabetologists.
**Young et al. (2008)[(3)]**	From laboratory data and diabetes-related complications:<br>• Retinopathy<br>• Nephropathy<br>• Neuropathy<br>• Cerebrovascular<br>• Cardiovascular<br>• Peripheral vascular disease<br>• Metabolic<br><br>**Outcomes:**<br>Risk of mortality, hospitalisation and healthcare utilisation.	Developed the Diabetes Complication Severity Index (DCSI) that is categorised to two or three levels (0=no abnormality; 1=some abnormality, 2=severe abnormality) depending on the severity of the complication. So if no abnormalities were present, the patient received no score, a 1 was added to the DCSI if patient had any complication classified as some abnormality and 2 was added if patient had any complication classified as severe abnormality.	The study design was a validation sample

References

1. Gini R, Schuemie MJ, Mazzaglia G, Lapi F, Francesconi P, Pasqua A, et al. Automatic identification of type 2 diabetes, hypertension, ischaemic heart disease, heart failure and their levels of severity from Italian General Practitioners' electronic medical records: a validation study. BMJ open. 2016;6(12):e012413. Epub 2016/12/13.
2. Grootenhuis PA, Snoek FJ, Heine RJ, Bouter LM. Development of a type 2 diabetes symptom checklist: a measure of symptom severity. Diabetic medicine : a journal of the British Diabetic Association. 1994;11(3):253-61. Epub 1994/04/01.
3. Young BA, Lin E, Von Korff M, Simon G, Ciechanowski P, Ludman EJ, et al. Diabetes complications severity index and risk of mortality, hospitalization, and healthcare utilization. Am J Manag Care. 2008;14(1):15-24.
