# Supplementary material for: Using electronic health records to quantify and stratify the severity of type 2 diabetes in primary care in England: rationale and cohort study design
Source: BMJ Open. 2018 Jun 30;8(6):e020926. doi: 10.1136/bmjopen-2017-020926 (PMC6042592; doi:10.1136/bmjopen-2017-020926)
Supplement: Author's manuscript [file bmjopen-2017-020926.draft_revisions.pdf]

BMJ Open

BMJ Open is committed to open peer review. As part of this commitment we make the peer review history of every article we publish publicly available.

When an article is published we post the peer reviewers' comments and the authors' responses online. We also post the versions of the paper that were used during peer review. These are the versions that the peer review comments apply to.

The versions of the paper that follow are the versions that were submitted during the peer review process. They are not the versions of record or the final published versions. They should not be cited or distributed as the published version of this manuscript.

BMJ Open is an open access journal and the full, final, typeset and author-corrected version of record of the manuscript is available on our site with no access controls, subscription charges or pay-per-view fees (http://bmjopen.bmj.com).

If you have any questions on BMJ Open's open peer review process please email info.bmjopen@bmj.com

BMJ Open

Protocol for a cohort study to quantify and stratify the severity of type 2 diabetes in primary care in England: rationale and study design

Journal:	*BMJ Open*
Manuscript ID	bmjopen-2017-020926
Article Type:	Protocol
Date Submitted by the Author:	01-Dec-2017
Complete List of Authors:	Zghebi, Salwa; University of Manchester, NIHR School for Primary Care Research, Centre for Primary Care<br>Rutter, Martin; University of Manchester Faculty of Biology, Medicine and Health, Division of Diabetes, Endocrinology and Gastroenterology; Manchester University NHS Foundation Trust, Manchester Diabetes Centre<br>Ashcroft, Darren; University of Manchester<br>Salisbury, Chris; University of Bristol, Academic Unit of Primary Health Care<br>Mallen, Christian; Keele University, Arthritis Research UK Primary Care Centre<br>Chew-Graham, Carolyn; University of Keele<br>Reeves, David; University of Manchester, Institute of Population Health; Centre for Biostatistics<br>Van Marwijk, H; The University of Manchester, Centre for Primary Care<br>Qureshi, Nadeem; University of Nottingham, of Primary Care<br>Weng, Stephen; University of Nottingham, NIHR School of Primary Care Research<br>Peek, N; Health e-Research Centre, Institute of Population Health, University of Manchester<br>Planner, Claire; NIHR School for Primary Care Research, Centre for Primary Care, Institute of Population Health<br>Mamas, Mamas; Keele Cardiovascular Research group, Centre for Prognosis Research<br>Kontopantelis, Evangelos; University of Manchester, Faculty of Biology, Medicine and Health; NIHR School for Primary Care Research, Centre for Primary Care
**Primary Subject Heading**:	Diabetes and endocrinology
Secondary Subject Heading:	General practice / Family practice, Health services research
Keywords:	Type 2 diabetes, Diabetes Severity, Electronic Health Records, PRIMARY CARE, Hospitalisation records, Cardiovascular disease

SCHOLARONE™
Manuscripts

For peer review only

Protocol for a cohort study to quantify and stratify the severity of type 2 diabetes in primary care in England: rationale and study design

Salwa S Zghebi,[1] Martin K Rutter,[2,3] Darren M Ashcroft,[4] Chris Salisbury,[5] Christian Mallen,[6] Carolyn A. Chew-Graham,[6] David Reeves,[1] Harm Van marwijk,[1] Nadeem Qureshi,[7] Stephen Weng,[7] Niels Peek,[8] Claire Planner,[1] Mamas Mamas,[9] Evangelos Kontopantelis[1,8]

1. Centre for Primary Care, School of Health Sciences, Faculty of Biology, Medicine and Health, Health Innovation Manchester, University of Manchester, Manchester, UK.

2. Division of Diabetes, Endocrinology and Gastroenterology, School of Medical Sciences, Faculty of Biology, Medicine and Health, University of Manchester, Manchester, M13 9PL, UK.

3. Manchester Diabetes Centre, 193 Hathersage Rd, Manchester University NHS Foundation Trust, Manchester Academic Health Science Centre,, Manchester, M13 0JE, UK.

4. Centre for Pharmacoepidemiology and Drug Safety, School of Health Sciences, Faculty of Biology, Medicine and Health, Health Innovation Manchester, University of Manchester, Manchester, UK.

5. Centre for Academic Primary Care, Population Health Sciences, Bristol Medical School, University of Bristol, Bristol, UK.

6. Research Institute for Primary Care and Health Sciences, Keele University, Staffordshire, UK.

7. School of Medicine, University of Nottingham, Nottingham, UK.

8. Division of Informatics, School of Health Sciences, Faculty of Biology, Medicine and Health, Imaging & Data Sciences (L5), Health Innovation Manchester, University of Manchester, Manchester, UK.

9. Keele Cardiovascular Research group, Centre for Prognosis Research, Institute for Primary Care and Health Sciences, Stoke-on-Trent, UK.

Corresponding author. Salwa Zghebi. Centre for Primary Care, University of Manchester, Manchester, UK. Email address: Salwa.Zghebi@manchester.ac.uk

Number of words (Abstract): 300
Number of words (full text): 3,998
Number of Tables 1
Number of Figures: 4

For peer review only - http://bmjopen.bmj.com/site/about/guidelines.xhtml

Abstract

Introduction: The increasing prevalence of type 2 diabetes (T2DM) presents a significant burden on affected individuals and health-care systems internationally. There is, however, no agreed validated measure to infer diabetes severity from electronic health records (EHRs). We aim to quantify T2DM severity and validate it in relation to clinical adverse outcomes.

Methods and Analysis: Routinely-collected primary care data from the Clinical Practice Research Datalink (CPRD), linked hospitalisation and mortality records between January-2006 and December-2016 for T2DM patients in England will be used to develop a clinical algorithm to grade T2DM severity. The EHR-based algorithm will incorporate main risk factors (severity domains) for adverse outcomes to stratify T2DM case cohorts by baseline and longitudinal severity scores. Provisionally, the severity domains for T2DM, identified through a systematic review and expert opinion are: diabetes duration, HbA_{1c}, diabetes microvascular complications, comorbidities, and co-prescribed treatments. Experts by experience will review and agree domains prior to finalising the algorithm. Survival analysis for the association between the measured severity scores and future outcomes: cardiovascular events (myocardial infarction, stroke); hospitalisation (diabetes-related, cardiovascular admissions); and mortality (diabetes-related, cardiovascular, and all-cause mortality) will be performed as a statistical validation. The proposed EHR-based approach will quantify the T2DM severity for primary care performance management and inform the methodology for measuring the severity of other chronic conditions managed in primary care. We anticipate that the developed severity algorithm will be a practical tool for practitioners, aid clinical management decision-making, inform stratified medicine approaches to management, support future clinical trials and contribute to more effective service-planning and policy-making.

Ethics and Dissemination: The study protocol was approved by the Independent Scientific Advisory Committee (ISAC) (Reference:17_168R, September-2017). Some data were presented at the NIHR School for Primary Care Research Showcase, September-2017, Oxford,UK. The study findings will be disseminated in relevant academic conferences and peer-reviewed journals.

For peer review only

For peer review only - http://bmjopen.bmj.com/site/about/guidelines.xhtml

Strengths and limitations of this study

- This is the first UK-based study to develop a diabetes severity scoring tool based on real-world electronic healthcare data to grade people with type 2 diabetes by their clinical severity.
- The study will include a large sample size and use high-quality medical data routinely-collected from general practices with access to linked national hospitalisation and cause-specific mortality datasets.
- The association between the computed severity scores and future adverse outcomes (cardiovascular event, hospitalisation and mortality) will be used to validate the developed severity algorithm.
- There is a possibility to miss other severity indicators not recorded in the data sources used such as pharmacy dispensing data.
- Given that the linkage scheme is only available for consented English general practices, the study cohort will be restricted to eligible patients registered within England (form nearly 58% of total CPRD general practices).

For peer review only

For peer review only - http://bmjopen.bmj.com/site/about/guidelines.xhtml

Introduction

The worldwide prevalence of diabetes mellitus is increasing with World Health Organisation (WHO) estimating that over 422 million adults had diabetes in 2016 with a growth in the global prevalence from 4.7% in 1980 to 8.5% in 2014.[(1,2)] In the UK, the prevalence of type 2 diabetes (T2DM) has nearly doubled between 2004 and 2014.[(3,4)] People with diabetes have a higher risk for morbidity and mortality when compared to individuals without diabetes,[(3,5)] and hence the increasing prevalence presents a significant burden on health care resources. Diabetes management expenses are estimated to consume up to 11% of the total health care budget in the UK and USA.[(6,7)] Diabetes is a complex metabolic condition, of an increasing severity,[(8,9)] at individually varying levels, and progressive development of vascular complications and end organ damage over time. Diabetes is mainly managed in primary care in the UK,[(10)] overall Europe,[(11)] USA,[(12)] and Asia.[(13,14)] In the UK, nearly 75% of the diabetes-associated costs relate to management of diabetic complications.[(6)]

The 'severity' of clinical conditions can be conceptualised as a progression of the underlying disease process. Increasing disease severity and development of associated complications lead to greater treatment complexity and clinical impact. The severity of chronic conditions, such as diabetes, has not been widely considered despite the clinical relevance and its likely impact on health care systems, where few studies have assessed disease severity among patients with T2DM or have quantified temporal trends or scores of severity.[(15,16)]

Severity scores could be clinically useful, particularly in T2DM, as such a new summary score could contribute above what existing risk scores offer (e.g. moving beyond risk for death) and also be highly relevant for use in six main clinical and research areas: i) identifying complex patients with a higher need for future care; ii) identifying patients at early stages of disease (benchmarking); iii) providing information that will directly inform clinical care; iv) identifying trajectories in severity over time; v) providing research data such as serving as an important diabetes-specific covariate to consider in current research, similarly to the Charlson Comorbidity Index; and vi) providing data that will inform resource allocation in health systems, such as the National Health Service (NHS).

For peer review only - http://bmjopen.bmj.com/site/about/guidelines.xhtml

Here, we describe the conceptual development of a T2DM severity algorithm model, using electronic health records (EHRs) on clinical consultations and treatments. Our aim is to use routinely-collected clinical and administrative data to develop a scoring tool which would quantify and grade the severity of T2DM. This model could potentially be used by clinicians to stratify patients based on disease severity and we aim to demonstrate potential advantages over similar risk scores. To validate the algorithm, our secondary aim is to examine the association of severity grades with risk of three main adverse outcomes: cardiovascular disease, hospitalisation, and mortality. The developed severity stratification algorithm is anticipated to have direct impact on clinical practice and have wider implications on service-planning and policy-making.

Methods and Analysis

Data Source

This study will use data from the Clinical Practice Research Datalink (CPRD). The CPRD is one of the world's largest EHR with anonymised primary care records from over 650 general practices over the UK.[17] Nearly 76% of all CPRD registered patients are in England.[17] CPRD data include clinical diagnoses, prescribed therapies, biochemical test results and referrals to healthcare services. The accuracy and completeness of diagnostic coding and validity of CPRD data, in clinical research, have been reported as excellent with positive predictive values (PPVs) of over 90% for nearly 14 conditions (including diabetes and cerebrovascular disease).[18] PPVs were defined as the proportion of CPRD diagnoses that were validated as true cases when compared to a gold standard such as GP questionnaire or primary care records.

Two metrics for research-quality data, recommended to be used by researchers, are available for CPRD records.[17] For patient-level data, the flag 'acceptable' indicates that a patient's record has met certain quality standards such as registration status, valid age and gender, and record of patient events. For practice-level data, the up-to-standard (UTS) date is used as a data quality measure. The UTS date is calculated for each CPRD general practice as the latest date at which the general practice meets minimum quality criteria based on

For peer review only - http://bmjopen.bmj.com/site/about/guidelines.xhtml

two central concepts: the continuity of recorded data and the number of recorded deaths, in comparison to an expected national range.[17,19]

Nearly 75% of general practices in England (approximately 58% of all CPRD practices) have consented to the CPRD Linkage Scheme for access to a number of linked datasets and national disease registries.[17] These include hospital data (including outpatient, admissions, and Accident and Emergency (A&E) data), mortality records (held by the Office for National Statistics (ONS)), socio-economic status and the cancer registry. The linked hospitalisation records, held by the Hospital Episode Statistics (HES), provide ethnicity data, admission and discharge dates, clinical diagnoses and procedures during hospitalisation recorded using the tenth revision of the International Classification of Diseases (ICD-10) and operating procedure codes (OPCS). This study will only include patients registered with English general practices which provide up-to-standard data and which participate in the CPRD Linkage Scheme.

Study Population

The study cohort will include individuals aged 35 years or over with T2DM (with ≥1 diagnostic code as in Table S1) between 1st January 2006 and 31st December 2016. Patients with an ever record of type 1 diabetes (with ever code for type 1 diabetes as in Table S2) will be excluded unless have records of non-insulin antidiabetic therapies. For this cohort construction, we will consider implementing previously-validated algorithms designed to identify diabetes cases by avoiding potential misclassification in routinely-collected data such as CPRD.[20,21] Eligible patients will be followed-up until censored at the earliest instance of any of the following event dates: patient transferred out of the practice (any cause), last collection date for the practice, the study end on the 31st December 2016 or death. The main demographic and clinical characteristics of the defined diabetes cohorts will be identified. Based on our previous studies, the expected sample size in a year will include 11,000 T2DM patients registered with English general practices linked to the HES Admitted Patient Care dataset.

For peer review only - http://bmjopen.bmj.com/site/about/guidelines.xhtml

Severity Domains

A systematic literature search for studies that developed algorithms or models to assess and quantify the severity of diabetes was conducted to identify the domains and sub-domains for T2DM severity (to be published separately). Also, expert clinical opinion from members of the research team was used to supplement the search process and identify possible omissions. Clinical members in the team include pharmacists (DMA, SSZ), general practitioners (CS, CM, CCG, HVm, and NQ), a consultant diabetologist (MKR) and a consultant cardiologist (MM), who used their expertise to create a list of relevant clinical domains for T2DM severity. The final domains to be included in the severity model will be decided during the analysis stage. Currently, the identified clinical domains that are relevant to the degree of progression of T2DM include: patient factors (diabetes duration and body mass index (BMI)), monitoring laboratory tests (glycated haemoglobin (HbA_{1c}), blood glucose levels), type of antidiabetic therapy, other prescribed medications (such as lipid-regulating medications and angiotensin-converting enzyme inhibitors (ACEIs)), comorbidities (including diabetes-related complications, depression), hospitalisation, and surgical interventions. The identified domains and sub-domains are described in Table 1. Domains will also be reviewed by a panel of 'experts by experience' (people with lived experience of T2DM) to provide patient validation of the severity scoring tool.

The clinical Read codes for the defined severity domains codes and the product codes for drug therapies will be identified using the [pcdsearch] Stata command.[22] The corresponding ICD-10 codes will be used to identify clinical domains recorded in the hospitalisation data and ONS mortality records. The relevant CPRD operational identification entities for the laboratory tests will be identified. This is an ongoing process whereby the final lists of domains and codes will be reached by consensus among the clinical members of the team. All code lists will be available on the online clinical code repository (ClinicalCodes.org).[23]

For peer review only - http://bmjopen.bmj.com/site/about/guidelines.xhtml

Study Outcomes

The adverse outcomes of interest will be the development of the first event among cardiovascular disease, (myocardial infarction, stroke), future hospitalisation (any hospitalisation, diabetes-related and cardiovascular hospitalisation) and death (diabetes-related mortality, cardiovascular mortality and all-cause mortality). Secondary outcome will be future hospitalisation due to hypoglycaemia, a relevant and potentially preventable adverse outcome. The hospitalisation and death outcomes will be identified using the linked HES and ONS mortality data, respectively. Similarly with severity domains, Read codes and ICD-10 codes will be used to identify the outcomes as appropriate.

Statistical Analyses

Diabetes Severity Algorithm

Using annual data bins and grouping diabetes patients from 1st January to 31st December in a calendar year between 2006 to 2016, the developed diabetes algorithm will grade the severity of T2DM using pre-defined (sub)domains.

We will consider three approaches to derive numerical or categorical diabetes severity scores or levels. Firstly, we will use a binary classification (severity indicator: present/absent) within each sub-domain and calculate an aggregate score. The second approach, through hierarchical stratification of end organ microvascular and macrovascular complications, will involve increasing weighting within each sub-domain, as severity increases where scores of 1, 2, or 3 on each subdomain will be assigned based on clinical input in terms of severity. Then, we will use the model estimates to weight sub-domains (according to weights calculated from t-values of the fitted regression model). The highest possible severity score will be known when the final list of included domains and sub-domains is decided. Hierarchical diagrams of the clinical severity of identified diabetes-related complications domains (Figure 1) cerebrovascular (Figure 2), and cardiovascular (Figure 3, Figure 4, Figure S1, Figure S2) domains are presented. The third approach will be

For peer review only - http://bmjopen.bmj.com/site/about/guidelines.xhtml

based on decision-trees developed to categorise severity of T2DM into four categories: high, moderate, mild, and low severity. The trees will combine severity domains and sub-domains that define each severity category. For instance, the high severity category may include the presence of two or more diabetes-related compilations, HbA_{1c} ≥10% (≥86mmol/mol), insulin use, and evidence of HF. Whereas, the low severity category may include severity domains of HbA_{1c} ≤7% (≤53mmol/mol), diabetes managed with diet/lifestyle measures or a single oral antidiabetic agents, absence of microvascular complications, none or one cardiovascular risk factor. Statistical packages that enable plotting decision trees are available.[24,25] The third approach would help to identify what combinations of complications and severity markers are most highly predictive of adverse outcomes.

Sub-domains with many measurements over time, such as HbA_{1c} levels and BMI, will be modelled as time-varying covariates (alternative models will be considered to account for non-linear changes). Within each time window the average of these measurements will be used, and we will also use multiple imputation approaches to account for missing data. Provisionally, we will consider using data records within look-back periods between 3-5 years. Different look-back windows within this period will be tested to obtain the optimal time window. Descriptive statistics of the study population and the estimated scores will be performed.

Severity Algorithm Validation

When we have developed a first version of the severity score tool, it will be validated statistically by quantifying the association of severity scores to future adverse outcomes. The primary outcome will be developing a cardiovascular event (myocardial infarction, stroke), future hospitalisation and death (diabetes-related mortality, cardiovascular mortality and all-cause mortality). A secondary adverse outcome will be future hospital admission due to hypoglycaemia, a relevant and potentially preventable adverse outcome. Cox proportional hazards (after assessing proportional hazards assumption) and/or competing-risks regressions will be used to perform survival analyses and assess the relationship between the severity score and outcomes controlling for all available patient characteristics (such as age, gender, ethnicity, and level of social deprivation). We aim to analyse and assess how severity scores differ across different levels of these variables. The

For peer review only - http://bmjopen.bmj.com/site/about/guidelines.xhtml

Cox regression analysis will form the basis of the validation for the developed severity algorithm and we will assess the model performance using relevant measures (such as area under the receiver operating characteristic curve (AUROC). Using this analysis, we will also test what how much the 'sophisticated' full model that includes all relevant severity domains will add in terms of predictive validity of the adverse outcomes over a simpler model that includes demographic data (age, gender and level of social deprivation) alone.

Longitudinal Trends of Diabetes Severity

In newly diagnosed T2DM patients during the study period we will assess the temporal trends of severity scores over time by calculating the time needed to progress along the quintiles of the severity scale starting from the date or year of diagnosis and identify the predictors to this progression across quintiles.

While the inclusion of some domains such as carotid artery stenting, the use of ACEIs or lipid-regulating drugs, kidney transplantation and laser therapy are clinically-relevant and represent markers of greater level of disease severity they also improve the prognosis (lessen the grade of severity) i.e. raising a risk of "feedback". While we will further assess their role, we may have to not include these domains in the modelling at the analysis stage. All statistical analyses will be conducted using Stata Statistical Software for Windows: *Release 15* (College Station, TX: StataCorp LP, USA).

Patient and Public Involvement and Engagement (PPIE)

The public interest in the reuse of routinely-collected electronic health data for research purposes has expanded over recent years. Our study presents a valuable opportunity to address the challenge of how to develop meaningful and productive PPIE collaborations in observational studies that make use of secondary data.

We will identify suitable PPIE partners to collaborate on this study and develop creative strategies for meaningful involvement as the project develops. Participants to provide their input that will be particularly valuable in providing feedback into the diabetes severity

For peer review only - http://bmjopen.bmj.com/site/about/guidelines.xhtml

algorithms, and deciding what information would be most useful to patients and how to be presented.

In the latter stages of the study, jointly with our PPIE partners, we will plan and deliver a patients' and carers' workshop where we will present emerging findings and seek feedback to inform further work and the dissemination strategy. This will include an exercise around weights of domains within the algorithm. PPIE work will be reported using the GRIPP (Guidance for Reporting Involvement of Patients and Public) checklist.[26]

For peer review only

Discussion

The global and country-specific prevalence of diabetes has increased substantially.[2,27-30] For T2DM, prevalence rates have doubled over the past two decades.[4,31,32] Diabetes diagnosis is associated with higher risk for morbidity and mortality in comparison to people without diabetes.[5] Despite the natural progression of most chronic diseases, including T2DM, and their important clinical implication on prognosis and medical resources utilisation, only a few studies have attempted to grade the severity of chronic conditions over time. Our proposed study aims to develop a contemporary algorithm to quantify the severity of T2DM, a highly prevalent condition, using routine EHR. The scoring tool will be based on clinically-relevant diagnoses and treatments modelled as severity domains and sub-domains. The algorithm will enable clinicians to grade T2DM patients according to the level of their disease severity, at baseline and longitudinally, as driven by the weights of the included clinical domains.

Prior Studies

Currently, there is limited data around validated severity measures that can be used in routine clinical practice managing patients with T2DM. Consequently, we are in parallel finalising a conducted systematic review for clinical-based diabetes severity models which will inform the refinement of this work. Here, we discuss three relevant diabetes severity measures that have been previously reported from countries outside the UK.

For peer review only - http://bmjopen.bmj.com/site/about/guidelines.xhtml

In the first study, Gini et al. (2016) categorised the severity of T2DM (N=300) into four levels based on insulin use and the presence of diabetic complications (Table S3).[15] A validation study in a random sample of cases was performed by interviewing their general practitioners. We however, aim to include more T2DM patients and consider more clinical data such as other coexisting conditions, non-insulin therapies, statins and ACEIs, that have been shown to be associated with reduced risk of adverse outcomes.

In the second study, a diabetes symptom checklist was created to measure the perceived symptom severity and assess the changes over time in 185 T2DM patients.[16] The checklist contained 34 items categorised by main clinical symptoms (Table S3). The patterns of comorbidities and prescribed treatments were reportedly associated with significant differences in the estimated severity scores.[16] The sample size was however relatively small and the checklist was based on patients' perception on diabetes symptoms severity.

Thirdly, Young et al. (2008), calculated a diabetes complications severity index (DCSI) in 4,229 patients with type 1 or type 2 diabetes in one US geographic region to examine its association with adverse outcomes (risk of hospitalisation and mortality). In comparison to using a simple numerical count of complications, DCSI found to be a better tool to predict adverse outcomes.[33] The authors used pharmacy (insulin use only) and laboratory data to compute the severity index and included patients from clinics with largest ethnic diversity. However, the DCSI missed additional domains such as diabetes duration, hyperlipidaemia and a wider range of diabetes-related and end-organ damage manifestations that we aim to assess in addition to hospitalisation and mortality.

In the wider literature, although several studies have investigated the possible role of other various factors on the severity of diabetes, none have provided a severity scoring tool that utilises data from various clinical domains as planned in our study that can use the wealth of information routinely-collected in electronic healthcare databases. In these studies, approaches used to define diabetes severity included: comparing T2DM severity, before and after obesity surgery;[34] examining the association between diabetes severity and either: haematological and immunological changes,[35] levels of urine citrate,[36] a biomarker for adverse outcomes;[37] grip strength;[38] or the use of complementary medicine to manage T2DM.[39]

For peer review only - http://bmjopen.bmj.com/site/about/guidelines.xhtml

Overall, the severity of T2DM has been assessed using the following domains: the complexity of antidiabetic treatment regimens[34] or HbA_{1c} levels;[36] a summary severity variable that includes vascular complications;[40] or a health status composite, number of comorbidities and patterns of treatments.[41] Other putative and less clinically-robust indicators or animal models were reportedly used to assess the severity of T2DM such as the effect of different patient education approaches on diabetes severity;[42] evaluating the effect of parental history;[43] and the role of genetic,[44,45] metabolic,[46] or inflammatory mediators.[47]

In comparison to our planned study, none of previous studies included as many routinely-collected clinically-relevant variables as in our more inclusive summary severity score algorithm or assessed the association of estimated scores with the various adverse outcomes included in our defined primary and secondary endpoints.

Potential Strengths and Limitations

Our proposed study has several potential strengths: First, it aims to present a contemporary measure to the available tools and the first UK-based study to develop an EHR-based severity scoring tool to grade patients by their T2DM severity. Second, the study uses high-quality real-world medical data routinely-collected from general practices. The use of routinely-collected data indicates that severity scores can be generated automatically with minimal effort. Third, the views of PPIE collaborators will be incorporated in the development of the severity tool. Fourth, we will access two linked datasets: hospitalisation (HES) data (to maximise data capture and reduce condition misclassification), and cause-specific mortality data to ascertain causes of death. Fifth, the sample size is expected to be large enough to drive the development and evaluation of the severity algorithm. Sixth, a statistical validation of the developed algorithm is planned and described.

As we will use data available in CPRD and HES datasets, one of the limitations we anticipate is the possibility to miss other severity indicators not recorded in used datasets. These include detailed pharmacy data such as ‘actual’ dispensing and adherence data. Also, the use of routinely-collected data is associated with missing values, being collected from

For peer review only - http://bmjopen.bmj.com/site/about/guidelines.xhtml

questionnaires, and issues around the accuracy of coding. However, we plan to use appropriate imputation methods and definite criteria to minimise the effect of coding issues. Another limitation is that our study will be restricted to patients registered with general practices in England, as CPRD currently only provides linkage to external national datasets for consented general practices in England. However, English general practices form the majority of all CPRD practices including nearly 76% of total registered patients.[17]

Importance and the Clinical Implications of the Severity Tool

The developed algorithm and severity tool may have significant implications for primary care both in terms of disease management and resource allocation. Ideally, through future work of further validation and assessment of clinical utility, the severity tool will be of practical use in primary care through its implementation in the clinical computing systems used in the UK.[48] The clinical significance of the developed severity algorithm to primary care is driven by the inclusion of highly-relevant clinical domains, such as diabetes-related complications and comorbidities, mapped to routinely-collected data.

Additionally, by assessing the longitudinal patterns of severity, the developed tool may be more clinically relevant than the currently used proxy (HbA_{1c}), and thus it could be a more reliable indicator in informing practices' remuneration for diabetes care. Categorising individuals based on their diabetes severity will be relevant for risk stratification (which may enable safer delegation of care within the clinical team), identify individualised patient risks and will help practitioners triage patients in need of a greater clinical input which informs towards stratified medicine to reduce future life-changing diabetes-related complications. Moreover, the weights of the severity scores may inform future clinical trials as the scoring tool considers a broader range of cardiovascular conditions than in most randomised clinical trials. Given the relatively low rates of cardiovascular outcomes in some trials, identifying diabetes patients who are at higher risk via our severity algorithm would help to power trials with overall longer term benefit for patients. Finally, the composite severity score may serve as an important confounding factor in future research, as for example, to match diabetes cases and controls in observational studies and clinical trials.

For peer review only - http://bmjopen.bmj.com/site/about/guidelines.xhtml

For peer review only

Conclusions

Despite the availability of rich routinely-collected electronic medical data, there are limited validated measures to quantify the severity of main chronic conditions managed in primary care utilising this important resource. We aim to develop a severity algorithm for type 2 diabetes to quantify disease severity using clinically-important domains derived from UK EHR. Our goal is that developed severity score data will provide clinicians with practical information that will influence management pathways. The findings could have important implications for service-planning and policy-making. The severity tool may provide a useful model and methodology towards the assessment of severity in other chronic medical conditions managed in primary care setting. Future research may involve further validation by testing the severity model in a separate primary care database.

Author Contributions

E.K. S.S.Z. M.M., M.K.R and H.V.m developed the study design and data analysis plan. S.S.Z. M.M., M.K.R and H.V.m agreed on provisional clinical code lists. S.S.Z. prepared the first draft of the manuscript, and E.K., M.M., M.K.R. and H.V.m critically reviewed initial versions. C.P. contributed to the planned PPIE work. D.R., C.C.G., C.M., N.P., D.M.A., C.S., N.Q., and S.W reviewed and critically edited the manuscript. All authors approved the final version of the protocol before submission. S.S.Z. is the guarantor.

Funding

This study is funded by the National Institute for Health Research School for Primary Care Research (NIHR SPCR), grant number 331. This report is independent research by the National Institute for Health Research. The views expressed in this publication are those of the authors and necessarily those of the NHS, the National Institute for Health Research or the Department of Health.

For peer review only - http://bmjopen.bmj.com/site/about/guidelines.xhtml

Competing Interests

E.K., H.V.m, M.M., D.R., C.C.G. C.P., C.M., N.P. declare no competing interests. S.S.Z. reports support by the NIHR SPCR during this study. D.M.A. has received grant funding from Abbvie and has served on advisory boards for Pfizer and GSK. M.K.R. has received educational grant support from MSD and Novo Nordisk; has modest stock ownership in GSK; and has consulted for Roche. N.Q. reports grants from the NIHR SPCR, during the conduct of the study. C.S. reports grants from NIHR SPCR, during the conduct of the study; grants from NHS CLAHRC West, grants from Avon Primary Care Research Collaborative, outside the submitted work. S.W. serves as a member of the Clinical Practice Research Datalink Independent Scientific Committee (ISAC) at the UK Medicines and Health Regulatory Agency.

Ethical Approval

The approval for the study protocol was obtained from the CPRD's Independent Scientific Advisory Committee (ISAC).

List of Abbreviations

ACEI: Angiotensin converting enzyme inhibitors; **CABG**: coronary artery bypass graft; **CHD**: coronary heart disease; **CKD**: chronic kidney disease; **CPRD**: Clinical Practice Research Datalink; **CRT**: cardiac resynchronization therapy; **DCSI**: diabetes complications severity index; **EHR**: electronic health record; **EVAR**: Endovascular aneurysm repair; **GRIPP**: Guidance for Reporting Involvement of Patients and Public; **HbA_{1c}**: glycated haemoglobin; **HES**: Hospital Episode Statistics; **HES A&E:** HES Accident & Emergency; **HF**: heart failure; **ICD**: implantable cardioverter defibrillator; **ICD-10**: the 10th revision of the International Classification of Diseases; **ISAC**: Independent Scientific Advisory Committee; **MI**: myocardial infarction; **NHS:** National Health Service; **ONS**: Office for National Statistics; **OPCS**: operating procedure codes; **PAD**: peripheral artery disease; **PCI**: percutaneous coronary intervention; **PPIE:** patient and public involvement and engagement; **PTCA**: percutaneous transluminal coronary angioplasty; **PVD**: peripheral vascular disease; **SVT**: supraventricular tachycardia; **TAVI**: transcatheter aortic valve implantation; **T2DM**: type 2 diabetes; **TIA**: transient ischemic attack; **VF**: ventricular fibrillation; **VT**: ventricular tachycardia; **WHO**: World Health Organization.

For peer review only - http://bmjopen.bmj.com/site/about/guidelines.xhtml

References

1. World Health Organization (WHO) media centre. Diabetes. [Fact sheet] 2016 [cited 2016 29 April]; Available from: http://www.who.int/mediacentre/factsheets/fs312/en/.

2. World Health Organization (WHO). Global Report on Diabetes. 2016 [cited 2017 11 February]; Available from: http://apps.who.int/iris/bitstream/10665/204871/1/9789241565257_eng.pdf.

3. Zghebi SS, Steinke DT, Carr MJ, Rutter MK, Emsley RA, Ashcroft DM. Examining Trends in Type 2 Diabetes Incidence, Prevalence and Mortality in the UK between 2004 and 2014. Diabetes, Obesity and Metabolism. 2017.

4. Sharma M, Nazareth I, Petersen I. Trends in incidence, prevalence and prescribing in type 2 diabetes mellitus between 2000 and 2013 in primary care: a retrospective cohort study. BMJ Open. 2016;6:e010210.

5. American Diabetes Association (ADA). Standards of Medical Care in Diabetes - 2015. Diabetes Care. 2015;38(Suppl 1):S1-S49.

6. Hex N, Bartlett C, Wright D, Taylor M, Varley D. Estimating the current and future costs of Type 1 and Type 2 diabetes in the UK, including direct health costs and indirect societal and productivity costs. Diabetic Medicine. 2012;29:855-62.

7. Walker BR, Colledge NR, Ralston SH, Penman ID. Davidson's Principles and Practice of Medicine. China: Elsevier 2014 [cited 2016 30 June]. Available from: http://www.elsevier-etextbooks.com/pdfreader/davidsons-principles-practice-medicine57814.

8. Walker BR, Colledge NR, Ralston SH, Penman ID. Davidson's Principles and Practice of Medicine: Elsevier; 2014 [cited 2017 11 May]. Available from: http://elsevierelibrary.co.uk/pdfreader/davidsons-principles-practice-medicine57814.

9. Nefs G, Pop VJM, Denollet J, Pouwer F. The Longitudinal Association between Depressive Symptoms and Initiation of Insulin Therapy in People with Type 2 Diabetes in Primary Care. Plos One. 2013;8(11).

10. Murrells T, Ball J, Cookson G, Maben J, Lee G, Griffiths P. Managing diabetes in primary care: how does the configuration of the workforce affect quality of care? National Nursing Research Unit, King's College London, 2013 November 2013. Report No.: Department of Health Policy Research Programme, ref. 016/0058.

11. Luk VE, Patricia S, Luc F, Liesbeth B, Jan DM. Priorities for diabetes primary care in Europe. Primary Care Diabetes. 2008;2(1):3-8.

12. Spann SJ, Nutting PA, Galliher JM, Peterson KA, Pavlik VN, Dickinson LM, et al. Management of Type 2 Diabetes in the Primary Care Setting: A Practice-Based Research Network Study. Ann Fam Med. 2006;4(1):23–31.

13. Tai T-Y, Chuang L-M, Tsai S-T, Huang B-Y, The Diabcare (Taiwan) Study Group. Treatment of Type 2 Diabetes Mellitus in a Primary Care Setting in Taiwan: Comparison with Secondary/Tertiary Care. Journal of the Formosan Medical Association = Taiwan yi zhi. 2006;105(2):105–17.

14. Mafauzy M. Diabetes control and complications in private primary healthcare in Malaysia. Med J Malaysia. 2005;60(2):212-7.

15. Gini R, Schuemie MJ, Mazzaglia G, Lapi F, Francesconi P, Pasqua A, et al. Automatic identification of type 2 diabetes, hypertension, ischaemic heart disease, heart failure and their levels of severity from Italian General Practitioners' electronic medical records: a validation study. BMJ open. 2016;6(12):e012413. Epub 2016/12/13.

16. Grootenhuis PA, Snoek FJ, Heine RJ, Bouter LM. Development of a type 2 diabetes symptom checklist: a measure of symptom severity. Diabetic medicine : a journal of the British Diabetic Association. 1994;11(3):253-61. Epub 1994/04/01.

17. Herrett E, Gallagher AM, Bhaskaran K, Forbes H, Mathur R, van Staa TP, et al. Data Resource Profile: Clinical Practice Research Datalink (CPRD). International Journal of Epidemiology. 2015;44(3):827-36.

For peer review only - http://bmjopen.bmj.com/site/about/guidelines.xhtml

18. Khan NF, Harrison SE, Rose PW. Validity of diagnostic coding within the General Practice Research Database: a systematic review. British Journal of General Practice. 2010;60(572):e128-e36.
19. Williams T, van Staa T, Puri S, Eaton S. Recent advances in the utility and use of the General Practice Research Database as an example of a UK Primary Care Data resource. Therapeutic Advances in Drug Safety. 2012;3(2):89-99.
20. de Lusignan S, Khunti K, Belsey J, Hattersley A, van Vlymen J, Gallagher H, et al. A method of identifying and correcting miscoding, misclassification and misdiagnosis in diabetes: a pilot and validation study of routinely collected data. Diabetic Medicine. 2010;27(2):203-9.
21. Wright AK, Kontopantelis E, Emsley R, Buchan I, Sattar N, Rutter MK, et al. Life Expectancy and Cause-Specific Mortality in Type 2 Diabetes: A Population-Based Cohort Study Quantifying Relationships in Ethnic Subgroups. Diabetes Care. 2017;40(3):338-45.
22. Olier I, Springate DA, Ashcroft DM, Doran T, Reeves D, Planner C, et al. Modelling Conditions and Health Care Processes in Electronic Health Records: An Application to Severe Mental Illness with the Clinical Practice Research Datalink. Plos One. 2016;11(2).
23. Springate DA, Kontopantelis E, Ashcroft DM, Olier I, Parisi R, Chamapiwa E, et al. ClinicalCodes: An Online Clinical Codes Repository to Improve the Validity and Reproducibility of Research Using Electronic Medical Records. PLoS ONE. 2014;9(6):e99825.
24. Glander S. Plotting trees from Random Forest models with ggraph. 2017 [cited 2017 16 November]; Available from: http://shiring.github.io/machine_learning/2017/03/16/rf_plot_ggraph.
25. Foster ZSL, Sharpton TJ, Grünwald NJ. Metacoder: An R package for visualization and manipulation of community taxonomic diversity data. PLOS Computational Biology. 2017;13(2):e1005404.
26. Staniszewska S, Brett J, Mockford C, Barber R. The GRIPP checklist: Strengthening the quality of patient and public involvement reporting in research. International Journal of Technology Assessment in Health Care. 2011;27(4):391-9.
27. Lipscombe LL, Hux JE. Trends in diabetes prevalence, incidence, and mortality in Ontario, Canada 1995–2005: a population-based study. Lancet. 2007;369:750-56.
28. Onat A, Hergenc G, Uyarel H, Can G, Ozhan H. Prevalence, incidence, predictors and outcome of type 2 diabetes in Turkey. Anadolu kardiyoloji dergisi : AKD = the Anatolian journal of cardiology. 2006;6(4):314-21.
29. International Diabetes Federation (IDF). IDF Diabetes Atlas - 7th Edition. 2015 [cited 2017 08 February]; Available from: http://www.diabetesatlas.org/.
30. Lin CC, Li CI, Hsiao CY, Liu CS, Yang SY, Lee CC, et al. Time trend analysis of the prevalence and incidence of diagnosed type 2 diabetes among adults in Taiwan from 2000 to 2007: a population-based study. BMC Public Health. 2013;13(318).
31. Gonzalez ELM, Johansson S, Wallander MA, Rodriguez LAG. Trends in the prevalence and incidence of diabetes in the UK: 1996-2005. J Epidemiol Community Health. 2009;63(4):332-6.
32. Evans JMM, Barnett KN, Ogston SA, Morris AD. Increasing prevalence of type 2 diabetes in a Scottish population: effect of increasing incidence or decreasing mortality? Diabetologia. 2007;50(4):729-32.
33. Young BA, Lin E, Von Korff M, Simon G, Ciechanowski P, Ludman EJ, et al. Diabetes complications severity index and risk of mortality, hospitalization, and healthcare utilization. Am J Manag Care. 2008;14(1):15-24.
34. Runkel M, Muller S, Brydniak R, Runkel N. Downgrading of type 2 diabetes mellitus (T2DM) after obesity surgery: duration and severity matter. Obesity surgery. 2015;25(3):494-9. Epub 2014/09/12.
35. Okano K, Araki M, Yamamoto M, Ishikawa T, Ichihara K, Yamada O. Exploration of hematological and immunological changes associated with the severity of type 2 diabetes mellitus in Japan. Nursing & health sciences. 2008;10(1):65-9. Epub 2008/02/09.
36. Fram EB, Moazami S, Stern JM. The Effect of Disease Severity on 24-Hour Urine Parameters in Kidney Stone Patients With Type 2 Diabetes. Urology. 2016;87:52-9. Epub 2015/11/04.

For peer review only - http://bmjopen.bmj.com/site/about/guidelines.xhtml

37. Eldor R, Klieger Y, Sade-Feldman M, Vaknin I, Varfolomeev I, Fuchs C, et al. CD247, a novel T cell-derived diagnostic and prognostic biomarker for detecting disease progression and severity in patients with type 2 diabetes. Diabetes care. 2015;38(1):113-8. Epub 2014/11/05.
38. Loprinzi PD, Loenneke JP. Evidence of a Link Between Grip Strength and Type 2 Diabetes Prevalence and Severity Among a National Sample of U.S. Adults. Journal of physical activity & health. 2016;13(5):558-61. Epub 2015/11/10.
39. Nahin RL, Byrd-Clark D, Stussman BJ, Kalyanaraman N. Disease severity is associated with the use of complementary medicine to treat or manage type-2 diabetes: data from the 2002 and 2007 National Health Interview Survey. BMC complementary and alternative medicine. 2012;12:193. Epub 2012/10/24.
40. Linzer M, Pierce C, Lincoln E, Miller DR, Payne SM, Clark JA, et al. Preliminary validation of a patient-based self-assessment measure of severity of illness in type 2 diabetes: results from the pilot phase of the Veterans Health Study. The Journal of ambulatory care management. 2005;28(2):167-76. Epub 2005/06/01.
41. Gatlin PK, Insel KC. Severity of Type 2 Diabetes, Cognitive Function, and Self-Care. Biological research for nursing. 2015;17(5):540-8. Epub 2014/11/14.
42. Windrum P, Garcia-Goni M, Coad H. The Impact of Patient-Centered versus Didactic Education Programs in Chronic Patients by Severity: The Case of Type 2 Diabetes Mellitus. Value in health : the journal of the International Society for Pharmacoeconomics and Outcomes Research. 2016;19(4):353-62. Epub 2016/06/23.
43. Svensson E, Berencsi K, Sander S, Mor A, Rungby J, Nielsen JS, et al. Association of parental history of type 2 diabetes with age, lifestyle, anthropometric factors, and clinical severity at type 2 diabetes diagnosis: results from the DD2 study. Diabetes/metabolism research and reviews. 2016;32(3):308-15. Epub 2015/09/27.
44. Powell DS, Maksoud H, Charge SB, Moffitt JH, Desai M, Da Silva Fihlo RL, et al. Apolipoprotein E genotype, islet amyloid deposition and severity of Type 2 diabetes. Diabetes research and clinical practice. 2003;60(2):105-10. Epub 2003/04/23.
45. Jiang Q, Lyu XM, Yuan Y, Wang L. Plasma miR-21 expression: an indicator for the severity of Type 2 diabetes with diabetic retinopathy. Bioscience reports. 2017;37(2). Epub 2017/01/22.
46. Campbell-Tofte J, Hansen HS, Mu H, Molgaard P. Increased lipids in non-lipogenic tissues are indicators of the severity of type 2 diabetes in mice. Prostaglandins, leukotrienes, and essential fatty acids. 2007;76(1):9-18. Epub 2006/11/14.
47. Garcia-Elorriaga G, Padilla-Reyes M, Cruz-Olivo F, Fuentes-Allen JL, del Rey-Pineda G, Gonzalez-Bonilla C. [Pro-inflammatory cytokines related to severity and mortality in type 2 diabetes patients with soft tissue infection]. Revista medica del Instituto Mexicano del Seguro Social. 2012;50(3):237-41. Epub 2012/11/28. Citocinas, diabetes e infeccion de tejidos blandos. Su relacion con la severidad y mortalidad.
48. Kontopantelis E, Buchan I, Reeves D, Checkland K, Doran T. Relationship between quality of care and choice of clinical computing system: retrospective analysis of family practice performance under the UK's quality and outcomes framework. BMJ open. 2013;3:e003190.
49. Dunning T, Martin M. Type 2 diabetes: is it serious? Journal of Diabetes Nursing. 1998;2(3):70-6.

For peer review only - http://bmjopen.bmj.com/site/about/guidelines.xhtml

Table 1 The main (sub) domains identified to quantify the severity of type 2 diabetes

	Severity domain	Severity sub-domain
**1.**	**Risk factors***	• Duration of type 2 diabetes[39]<br>• Body mass index (BMI)<br>• Hypertension<br>• Hyperlipidaemia<br>• Personal/Family history of cardiovascular disease<br>• Blood glucose levels<br>○ Glycated haemoglobin (HbA_{1c})[34]<br>○ Fasting (FBG) and random (RBG) blood glucose
**2.**	**Type/pattern of antidiabetic treatment, insulin use and other therapies**	• Antidiabetic therapy ever;[39] Therapies with cardiovascular benefits versus other; Changes in drug treatments;[49] or the number of prescribed treatments[41]<br>• Insulin use: prescription ever or within 1 year of diagnosis; Insulin initiation:[15] time to initiation<br>• Other therapies: angiotensin converting enzyme inhibitors (ACEI) and lipid-regulating therapies
**3.**	**Microvascular diabetic complications**[15,33]	• Neuropathy (Foot ulcer, Charcot foot, gangrene, amputation)<br>• Nephropathy<br>• Retinopathy (laser therapy and blindness)
**4.**	**Renal disease**	• Microalbuminuria and proteinuria<br>• Moderate-severe chronic kidney disease (CKD) stages 3 and 4[33]<br>• End-stage renal disease (ESRD): kidney transplant and dialysis
**5.**	**Cardiovascular and Cerebrovascular disease**	• Atherosclerosis[15,33]<br>• Myocardial infarction (MI) [15,33]<br>• Angina[15,33]<br>• Atrial/ventricular fibrillation (AF)/(VF)[33]<br>• Heart valve disease<br>• Heart failure (HF)[33]<br>• Peripheral vascular disease (PVD)[33]<br>• Transient ischemic attack (TIA)<br>• Ischaemic stroke, haemorrhagic stroke [15,33]
**6.**	**Cardiovascular and Cerebrovascular interventions**	• Coronary artery bypass graft (CABG)<br>• Coronary artery interventions (PCI/PTCA)<br>• Endovascular aneurysm repair (EVAR)<br>• PVD stenting and bypass procedures<br>• Heart valve interventions<br>• Use of defibrillator<br>• Carotid artery events, stenting and bypass interventions
**7.**	**Other comorbidities**	• Anxiety<br>• Depression<br>• Dementia<br>• Cognitive impairment
**8.**	**Hospital admissions**	• Any-cause hospital admissions<br>• Diabetes-attributable admission<br>• Cardiovascular disease-related admission
**9.**	**Emergency diabetes-related event**	• Hypoglycaemia<br>• Hyperosmolar hyperglycaemic state (HHS)[33]<br>• Diabetic ketoacidosis (DKA) or other coma[33]

**Other demographic data (such as age, gender and the level of deprivation) are important predictors for adverse outcomes and will be included in the later risk-prediction analysis.*

For peer review only

For peer review only - http://bmjopen.bmj.com/site/about/guidelines.xhtml

Figure 1 Severity hierarchy of microvascular diabetic complications

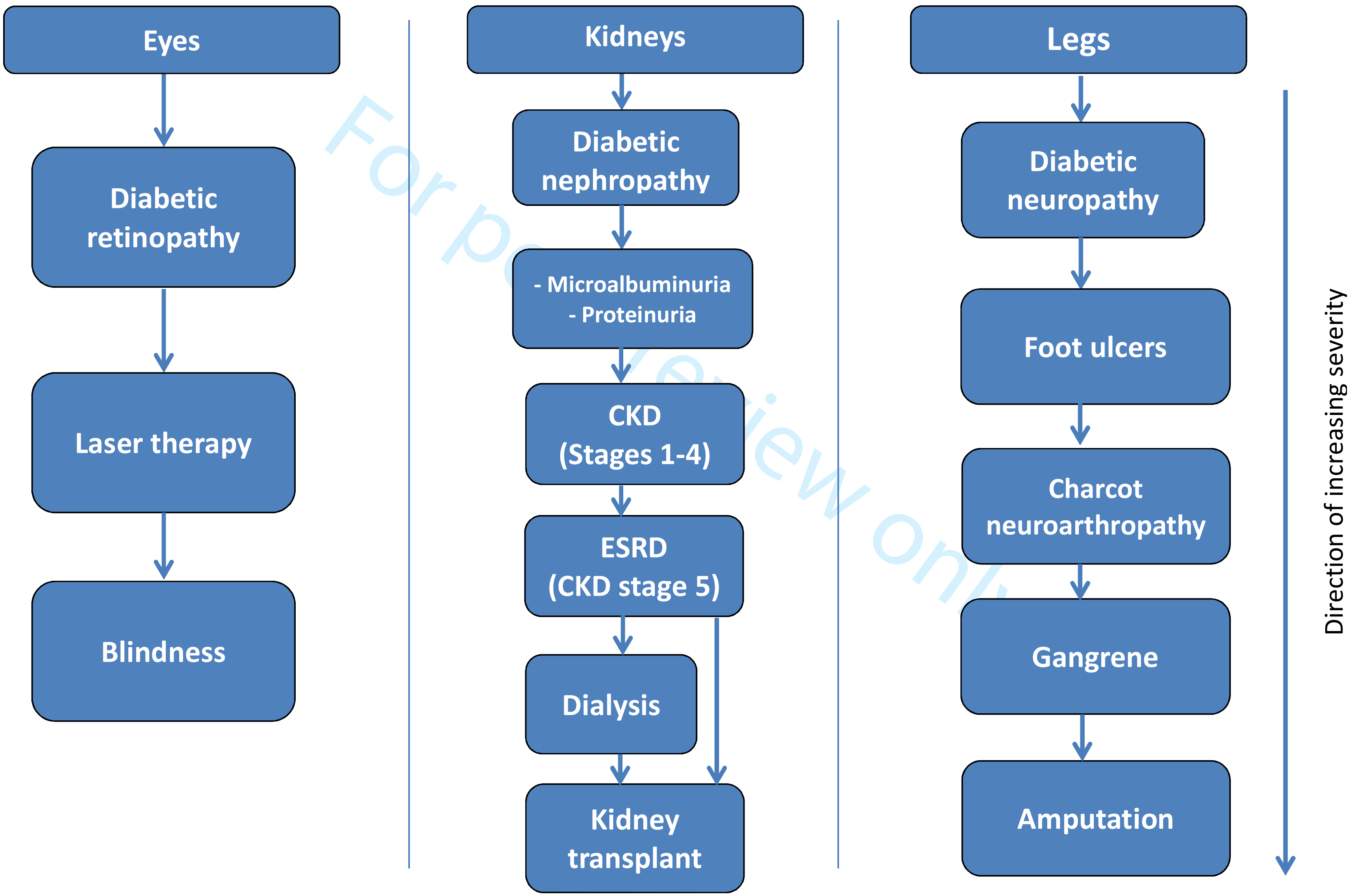

For peer review only - http://bmjopen.bmj.com/site/about/guidelines.xhtml

Figure 2 Severity hierarchy of cerebrovascular domains

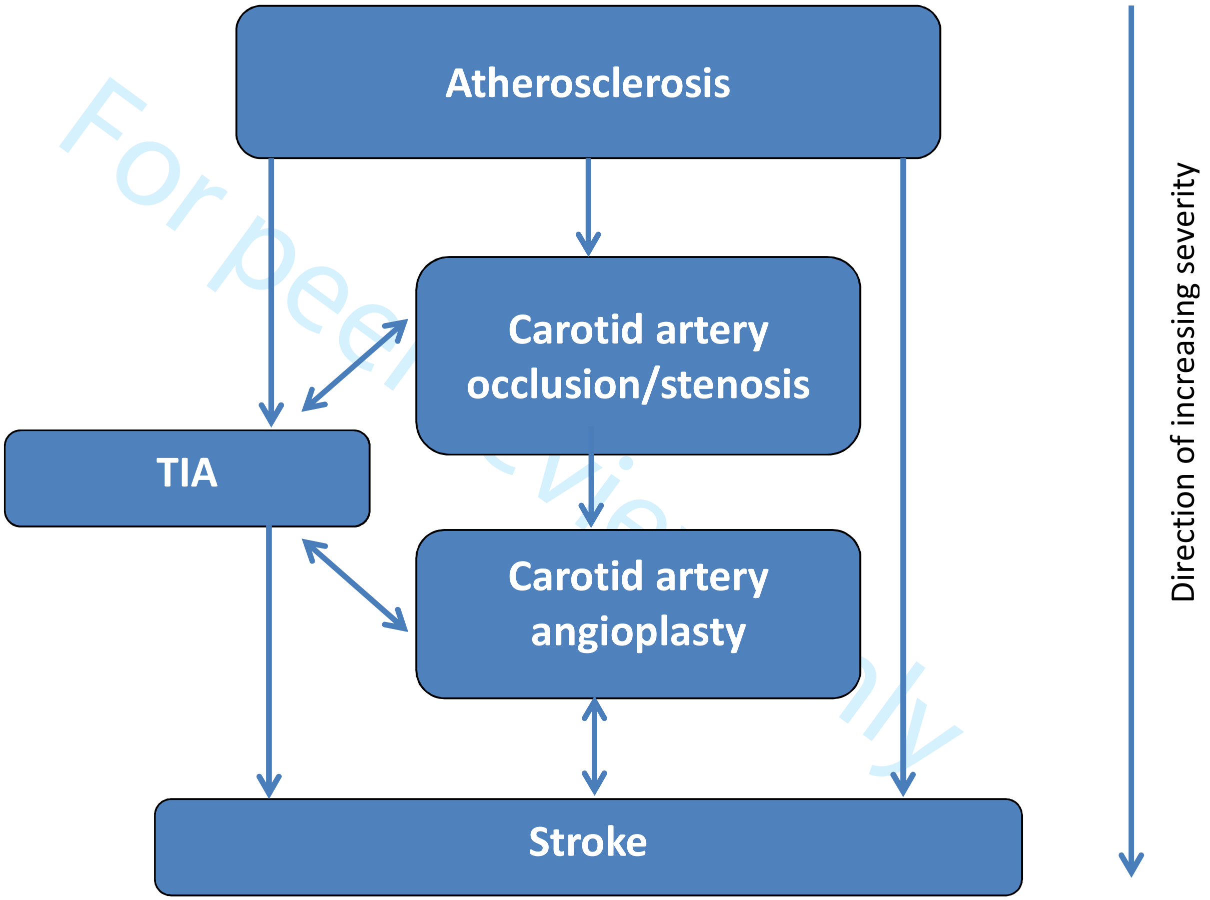

Figure 3 Severity hierarchy of coronary and vascular domains

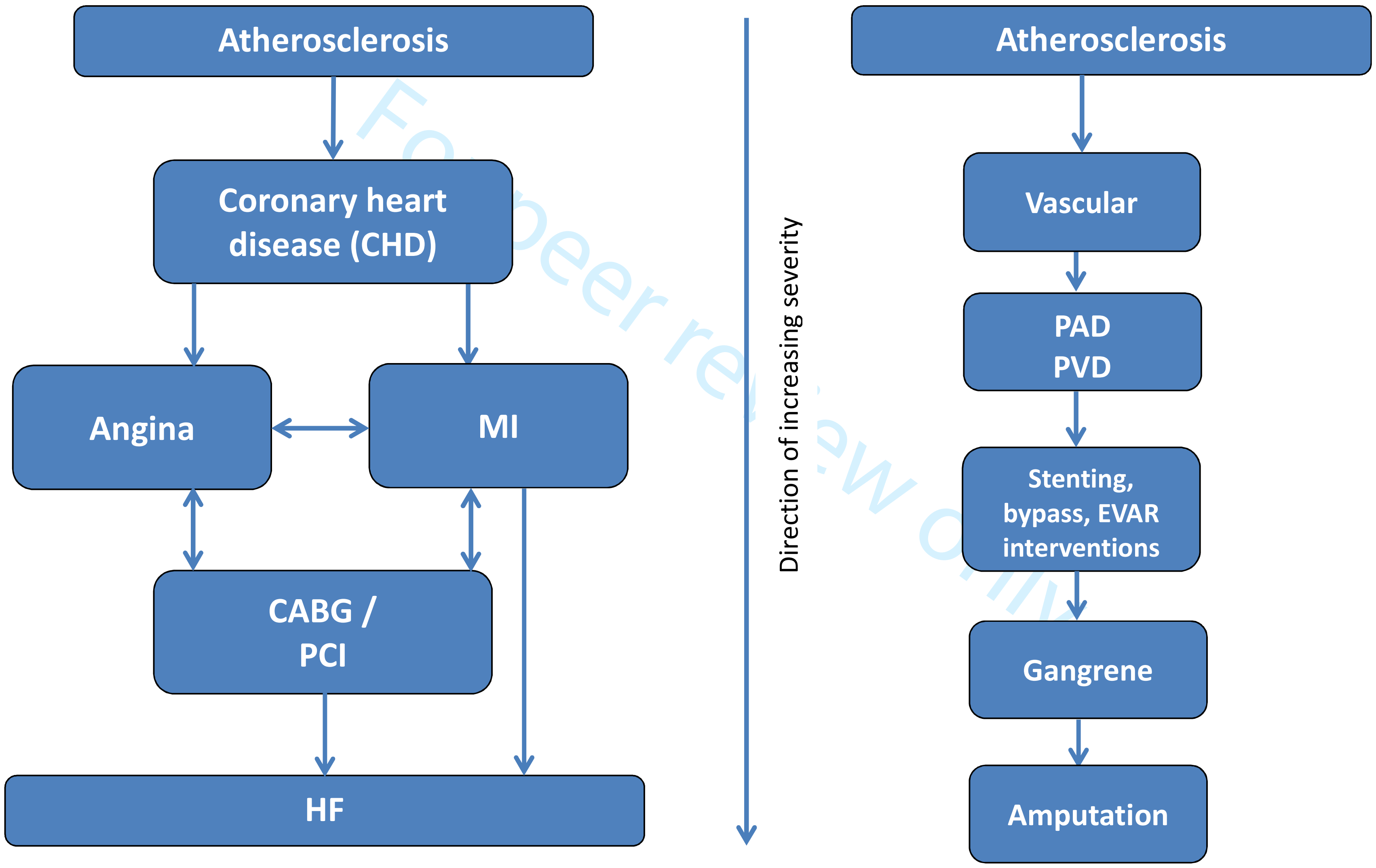

Figure 4 Severity hierarchy of cardiovascular disease domains (heart rate, rhythm and conduction)

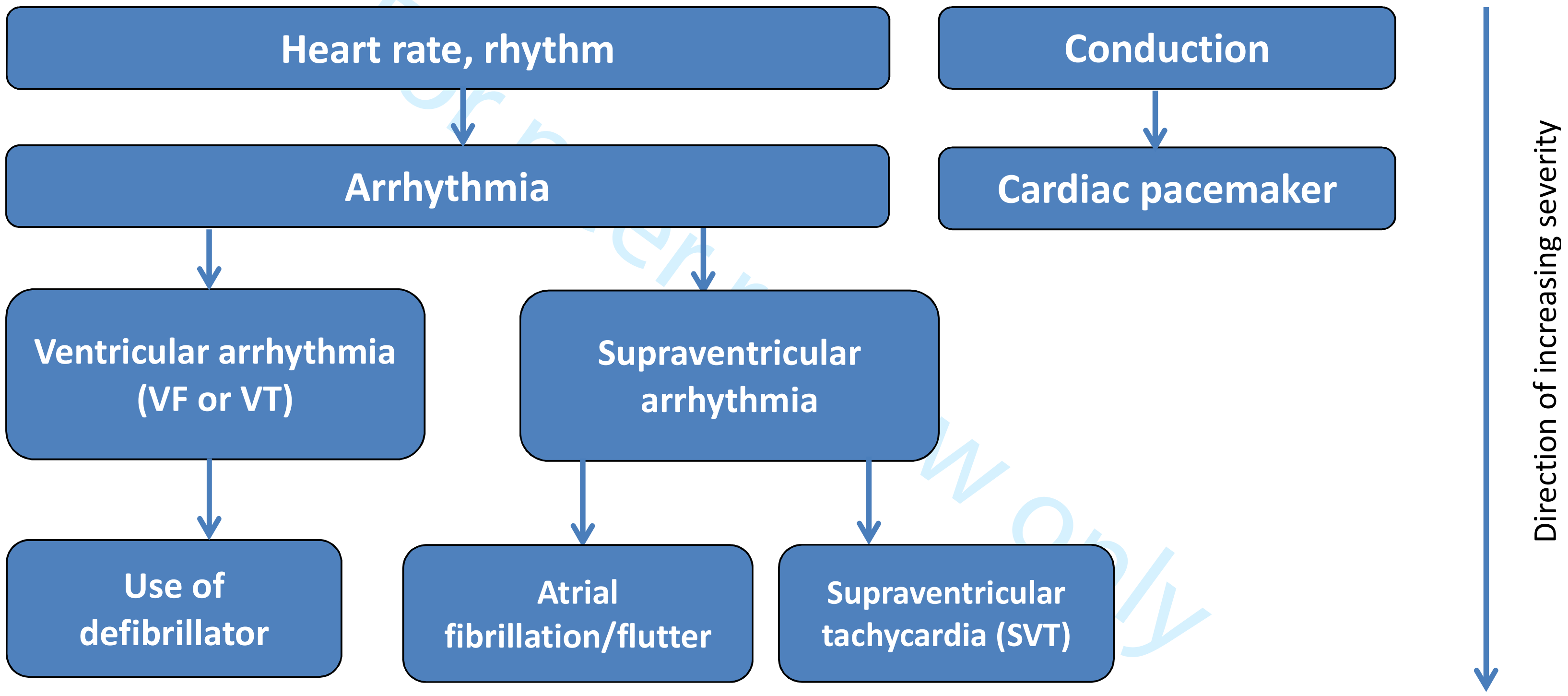

Supplementary data

Table S1 Inclusion medical codes for type 2 diabetes

	Medical code	Read term
**1.**	506	Non-insulin dependent diabetes mellitus
**2.**	758	Type 2 diabetes mellitus
**3.**	1407	Insulin treated Type 2 diabetes mellitus
**4.**	4513	Non-insulin dependent diabetes mellitus
**5.**	5884	NIDDM - Non-insulin dependent diabetes mellitus
**6.**	8403	Non-insulin dependent diabetes mellitus - poor control
**7.**	12640	Type 2 diabetes mellitus with nephropathy
**8.**	12736	Type 2 diabetes mellitus with gangrene
**9.**	17262	Non-insulin-dependent diabetes mellitus with retinopathy
**10.**	17859	Type 2 diabetes mellitus
**11.**	18143	Type II diabetes mellitus with arthropathy
**12.**	18209	Type 2 diabetes mellitus with renal complications
**13.**	18219	Type II diabetes mellitus
**14.**	18264	Insulin treated Type II diabetes mellitus
**15.**	18278	Insulin treated Type 2 diabetes mellitus
**16.**	18390	Type 2 diabetes mellitus with persistent microalbuminuria
**17.**	18425	Type 2 diabetes mellitus with polyneuropathy
**18.**	18496	Type 2 diabetes mellitus with retinopathy
**19.**	18777	Type 2 diabetes mellitus with renal complications
**20.**	22884	Type II diabetes mellitus
**21.**	24458	Type II diabetes mellitus - poor control
**22.**	24693	Non-insulin dependent diabetes mellitus with arthropathy
**23.**	24836	Type 2 diabetes mellitus with nephropathy
**24.**	25041	Dietary advice for type II diabetes
**25.**	25591	Type 2 diabetes mellitus with exudative maculopathy
**26.**	25627	Type 2 diabetes mellitus - poor control
**27.**	26054	Type 2 diabetes mellitus with persistent proteinuria
**28.**	29979	Non-insulin-dependent diabetes mellitus without complication
**29.**	32627	Type 2 diabetes mellitus with ketoacidosis
**30.**	34268	Type 2 diabetes mellitus with neurological complications
**31.**	34450	Hyperosmolar non-ketotic state in type 2 diabetes mellitus
**32.**	34912	Non-insulin dependent diabetes mellitus with ulcer
**33.**	35385	Type 2 diabetes mellitus with neuropathic arthropathy
**34.**	36633	Hyperosmolar non-ketotic state in type 2 diabetes mellitus
**35.**	37648	Insulin treated non-insulin dependent diabetes mellitus
**36.**	37806	Type 2 diabetes mellitus with peripheral angiopathy
**37.**	40401	Non-insulin dependent diabetes mellitus with gangrene
**38.**	42762	Type 2 diabetes mellitus with retinopathy
**39.**	43227	Type II diabetes mellitus with multiple complications
**40.**	43785	Non-insulin dependent diabetes mellitus with hypoglyca coma
**41.**	44779	Type 2 diabetes mellitus with diabetic cataract
**42.**	44982	Type 2 diabetes mellitus with diabetic cataract
**43.**	45467	Non-insulin dependent diabetes mellitus with polyneuropathy
**44.**	45913	Type 2 diabetes mellitus - poor control
**45.**	45919	Type 2 diabetes mellitus with neurological complications

For peer review only - http://bmjopen.bmj.com/site/about/guidelines.xhtml

**46.**	46150	Type 2 diabetes mellitus with gangrene
**47.**	46917	Type 2 diabetes mellitus with hypoglycaemic coma
**48.**	47315	Type II diabetes mellitus - poor control
**49.**	47321	Type 2 diabetes mellitus with ophthalmic complications
**50.**	47409	Type II diabetes mellitus with polyneuropathy
**51.**	47816	Type II diabetes mellitus with neuropathic arthropathy
**52.**	47954	Type 2 diabetes mellitus without complication
**53.**	48192	Type II diabetes mellitus with diabetic cataract
**54.**	49074	Type 2 diabetes mellitus with ulcer
**55.**	49655	Type II diabetes mellitus with retinopathy
**56.**	49869	Type 2 diabetes mellitus with arthropathy
**57.**	50225	Type II diabetes mellitus with renal complications
**58.**	50429	Non-insulin-dependent diabetes mellitus with ophthalm comps
**59.**	50527	Type II diabetes mellitus with polyneuropathy
**60.**	50609	Pre-existing diabetes mellitus, non-insulin-dependent
**61.**	50813	Type II diabetes mellitus with mononeuropathy
**62.**	51756	Type 2 diabetes mellitus with ketoacidotic coma
**63.**	52303	Non-insulin-dependent diabetes mellitus with renal comps
**64.**	53392	Type II diabetes mellitus without complication
**65.**	54899	Type II diabetes mellitus with peripheral angiopathy
**66.**	55075	Type II diabetes mellitus with ulcer
**67.**	55842	Non-insulin-dependent diabetes mellitus with neuro comps
**68.**	56268	Type II diabetes mellitus with hypoglycaemic coma
**69.**	57278	Type II diabetes mellitus with renal complications
**70.**	58604	Type II diabetes mellitus with retinopathy
**71.**	59253	Type 2 diabetes mellitus with arthropathy
**72.**	59365	Non-insulin dependent diabetes mellitus with nephropathy
**73.**	59725	Type II diabetes mellitus with ophthalmic complications
**74.**	60699	Type 2 diabetes mellitus with peripheral angiopathy
**75.**	60796	Type II diabetes mellitus with persistent proteinuria
**76.**	61071	Type 2 diabetes mellitus with hypoglycaemic coma
**77.**	62107	Type II diabetes mellitus with gangrene
**78.**	62146	Non-insulin-dependent diabetes mellitus with multiple comps
**79.**	62674	Type 2 diabetes mellitus with mononeuropathy
**80.**	63690	Type 2 diabetes mellitus with gastroparesis
**81.**	64571	Type II diabetes mellitus with nephropathy
**82.**	64668	Insulin treated Type II diabetes mellitus
**83.**	65267	Type 2 diabetes mellitus with multiple complications
**84.**	65704	Type 2 diabetes mellitus with ulcer
**85.**	66965	Type 2 diabetes mellitus with neuropathic arthropathy
**86.**	67905	Type II diabetes mellitus with neurological complications
**87.**	69278	Non-insulin depend diabetes mellitus with diabetic cataract
**88.**	70316	Type 2 diabetes mellitus with ophthalmic complications
**89.**	72320	Non-insulin dependent diabetes mellitus with mononeuropathy
**90.**	83532	Diabetes type 2 review
**91.**	85991	Type II diabetes mellitus with persistent microalbuminuria
**92.**	91646	Type II diabetes mellitus with ulcer
**93.**	93727	Type II diabetes mellitus with diabetic cataract
**94.**	95351	Type II diabetes mellitus with mononeuropathy
**95.**	98616	Type II diabetes mellitus with neurological complications
**96.**	98723	Type II diabetes mellitus with hypoglycaemic coma
**97.**	100964	Type II diabetes mellitus with ophthalmic complications
**98.**	101801	Type II diabetic dietary review
**99.**	102201	Type II diabetes mellitus with nephropathy

For peer review only - http://bmjopen.bmj.com/site/about/guidelines.xhtml

**100.**	102611	Type 2 diabetic dietary review
**101.**	103902	Type II diabetes mellitus with arthropathy
**102.**	104323	Type II diabetes mellitus with gangrene
**103.**	104639	Type II diabetes mellitus with peripheral angiopathy
**104.**	105784	Type 2 diabetes mellitus without complication
**105.**	106061	Type II diabetes mellitus with ketoacidotic coma
**106.**	106528	Type II diabetes mellitus with ketoacidosis
**107.**	107701	Hyperosmolar non-ketotic state in type II diabetes mellitus
**108.**	107824	Type II diabetes mellitus in remission
**109.**	108005	Type 2 diabetes mellitus with multiple complications
**110.**	109103	Type II diabetes mellitus without complication
**111.**	109197	Type II diabetes mellitus with neuropathic arthropathy
**112.**	109865	Type 2 diabetes mellitus with polyneuropathy

For peer review only

For peer review only - http://bmjopen.bmj.com/site/about/guidelines.xhtml

Table S2 Exclusion type 1 diabetes codes

	Medical code	Read term
**1.**	1038	Insulin dependent diabetes mellitus
**2.**	1549	Type 1 diabetes mellitus
**3.**	1647	Insulin dependent diabetes mellitus
**4.**	6509	Insulin dependent diabetes mellitus with retinopathy
**5.**	6791	Insulin dependent diabetes mellitus - poor control
**6.**	10418	Type 1 diabetes mellitus with nephropathy
**7.**	10692	Type 1 diabetes mellitus with ketoacidosis
**8.**	12455	Type I diabetes mellitus
**9.**	17545	Type I diabetes mellitus with diabetic cataract
**10.**	17858	Type 1 diabetes mellitus
**11.**	18230	Type 1 diabetes mellitus with neuropathic arthropathy
**12.**	18387	Type 1 diabetes mellitus with retinopathy
**13.**	18505	IDDM-Insulin dependent diabetes mellitus
**14.**	18642	Type 1 diabetes mellitus with arthropathy
**15.**	18683	Type 1 diabetes mellitus with ulcer
**16.**	21983	Type 1 diabetes mellitus with renal complications
**17.**	22871	Type 1 diabetes mellitus with exudative maculopathy
**18.**	24423	Type I diabetes mellitus
**19.**	24490	Diabetes mellitus, juvenile type, no mention of complication
**20.**	24694	Insulin dependent diabetes mellitus with mononeuropathy
**21.**	26855	Unstable insulin dependent diabetes mellitus
**22.**	30294	Type 1 diabetes mellitus with persistent microalbuminuria
**23.**	30323	Type 1 diabetes mellitus with persistent proteinuria
**24.**	31310	Insulin dependent diabetes maturity onset
**25.**	32359	Perceived control of insulin-dependent diabetes
**26.**	35288	Type 1 diabetes mellitus - poor control
**27.**	38161	Type I diabetes mellitus with retinopathy
**28.**	39070	Type 1 diabetes mellitus with hypoglycaemic coma
**29.**	39809	Insulin dependent diab mell with neuropathic arthropathy
**30.**	40023	Diabetes mellitus, juvenile type, with hyperosmolar coma
**31.**	40682	Type 1 diabetes mellitus maturity onset
**32.**	40837	Type 1 diabetes mellitus with ketoacidotic coma
**33.**	41049	Type 1 diabetes mellitus with retinopathy
**34.**	41716	Insulin dependent diabetes mellitus with polyneuropathy
**35.**	42567	Diabetes mellitus, juvenile type, with ketoacidotic coma
**36.**	42729	Type I diabetes mellitus with hypoglycaemic coma
**37.**	42831	Type 1 diabetes mellitus with neurological complications
**38.**	43921	Unstable type 1 diabetes mellitus
**39.**	44260	Insulin dependent diabetes mellitus with diabetic cataract
**40.**	44440	Insulin dependent diabetes mellitus with hypoglycaemic coma
**41.**	44443	Insulin dependent diabetes mellitus with ulcer
**42.**	45276	Insulin dependent diabetes mellitus with multiple complicat
**43.**	45914	Type 1 diabetes mellitus - poor control
**44.**	46301	Type 1 diabetes mellitus with polyneuropathy
**45.**	46850	Type I diabetes mellitus - poor control
**46.**	46963	Insulin-dependent diabetes mellitus with renal complications

For peer review only

**47.**	47582	Type 1 diabetes mellitus with renal complications
**48.**	47649	Type 1 diabetes mellitus with ophthalmic complications
**49.**	47650	Type 1 diabetes mellitus with multiple complications
**50.**	49146	Type I diabetes mellitus with neurological complications
**51.**	49276	Insulin-dependent diabetes mellitus with ophthalmic comps
**52.**	49554	Type 1 diabetes mellitus with diabetic cataract
**53.**	49949	Unstable type I diabetes mellitus
**54.**	50960	Pre-existing diabetes mellitus, insulin-dependent
**55.**	51261	Insulin dependent diabetes mellitus
**56.**	51957	Type I diabetes mellitus with ulcer
**57.**	52104	Insulin dependent diabetes mellitus with multiple complicatn
**58.**	52283	Insulin-dependent diabetes mellitus with neurological comps
**59.**	53200	Diabetes mellitus, juvenile type, with ketoacidosis
**60.**	54008	Type 1 diabetes mellitus with neuropathic arthropathy
**61.**	54600	Unstable insulin dependent diabetes mellitus
**62.**	55239	Type 1 diabetes mellitus with gastroparesis
**63.**	56448	Insulin-dependent diabetes without complication
**64.**	57621	Insulin dependent diabetes mellitus with nephropathy
**65.**	60107	Unstable type I diabetes mellitus
**66.**	60208	Type I diabetes mellitus with neuropathic arthropathy
**67.**	60499	Insulin dependent diabetes mellitus with gangrene
**68.**	61344	Type I diabetes mellitus with renal complications
**69.**	61829	Type 1 diabetes mellitus with neurological complications
**70.**	62209	Type I diabetes mellitus with ketoacidosis
**71.**	62352	Type I diabetes mellitus with arthropathy
**72.**	62613	Type I diabetes mellitus without complication
**73.**	63017	Type I diabetes mellitus maturity onset
**74.**	64446	Insulin dependent diab mell with peripheral angiopathy
**75.**	65616	Insulin dependent diabetes mellitus with arthropathy
**76.**	66145	Type I diabetes mellitus with ketoacidotic coma
**77.**	66872	Type I diabetes mellitus with nephropathy
**78.**	68105	Type 1 diabetes mellitus with mononeuropathy
**79.**	68390	Type 1 diabetes mellitus with ulcer
**80.**	68792	Diabetes mellitus, juvenile type, + unspecified complication
**81.**	69043	Dietary advice for type I diabetes
**82.**	69676	Type 1 diabetes mellitus without complication
**83.**	69748	Diabetes mellitus, juvenile type, + ophthalmic manifestation
**84.**	69993	Type 1 diabetes mellitus with gangrene
**85.**	70766	Type 1 diabetes mellitus with hypoglycaemic coma
**86.**	72702	Insulin dependent diabetes mellitus - poor control
**87.**	85660	Diabetes type 1 review
**88.**	91942	Type I diabetes mellitus with multiple complications
**89.**	91943	Type I diabetes mellitus with polyneuropathy
**90.**	93468	Type 1 diabetes mellitus with peripheral angiopathy
**91.**	93875	Insulin dependent diabetes mellitus with retinopathy
**92.**	93878	Type I diabetes mellitus with ulcer
**93.**	93922	Diabetes mellitus, juvenile type, with renal manifestation
**94.**	95343	Type I diabetes mellitus with retinopathy
**95.**	95992	Type I diabetes mellitus without complication
**96.**	96235	Type I diabetes mellitus maturity onset

For peer review only - http://bmjopen.bmj.com/site/about/guidelines.xhtml

**97.**	97446	Type 1 diabetes mellitus maturity onset
**98.**	97474	Unstable type 1 diabetes mellitus
**99.**	97849	Insulin dependent diabetes maturity onset
**100.**	97894	Type I diabetes mellitus with exudative maculopathy
**101.**	98071	Insulin-dependent diabetes mellitus with ophthalmic comps
**102.**	98392	Maturity onset diabetes in youth type 1
**103.**	98704	Insulin dependent diabetes mellitus with ulcer
**104.**	99231	Type I diabetes mellitus with mononeuropathy
**105.**	99311	Type I diabetes mellitus with ophthalmic complications
**106.**	99716	Insulin dependent diabetes mellitus with hypoglycaemic coma
**107.**	99719	Insulin-dependent diabetes without complication
**108.**	100770	Insulin dependent diabetes mellitus with diabetic cataract
**109.**	101311	Insulin dependent diabetes mellitus with polyneuropathy
**110.**	101735	Insulin-dependent diabetes mellitus with neurological comps
**111.**	102112	Type I diabetes mellitus with gangrene
**112.**	102163	Insulin dependent diabetes mellitus with nephropathy
**113.**	102620	Type I diabetes mellitus with persistent microalbuminuria
**114.**	102704	Type I diabetic dietary review
**115.**	102740	Type 1 diabetes mellitus with ophthalmic complications
**116.**	102946	Insulin-dependent diabetes mellitus with renal complications
**117.**	104453	Type 1 diabetic dietary review
**118.**	105337	Type I diabetes mellitus - poor control
**119.**	108007	Type I diabetes mellitus with multiple complications
**120.**	108360	Type I diabetes mellitus in remission
**121.**	108724	Type I diabetes mellitus with gastroparesis
**122.**	109051	Insulin dependent diabetes mellitus with gangrene
**123.**	109628	Type 1 diabetes mellitus in remission
**124.**	109837	Type I diabetes mellitus with renal complications
**125.**	109878	Diet advice for insulin-dependent diabetes
**126.**	110400	Type 1 diabetes mellitus with diabetic cataract
**127.**	111106	Type 1 diabetes mellitus without complication

For peer review only

Supplementary material to protocol "**Protocol for a cohort study to quantify and stratify the severity of type 2 diabetes in primary care in England: rationale and study design" Zghebi et al. 2017**

Figure S1 Severity hierarchy of cardiovascular disease domains (cardiac muscle- myocardium and pericardium).

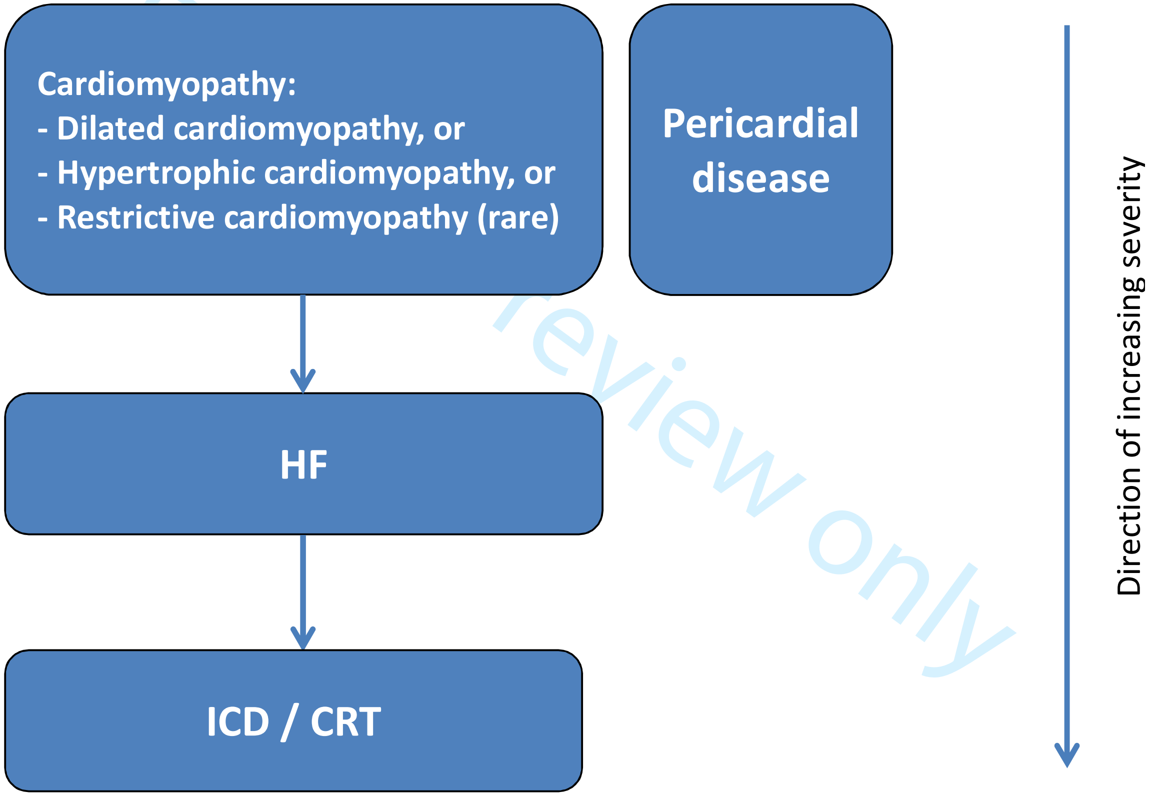

For peer review only - http://bmjopen.bmj.com/site/about/guidelines.xhtml

Supplementary material to protocol "**Protocol for a cohort study to quantify and stratify the severity of type 2 diabetes in primary care in England: rationale and study design" Zghebi et al. 2017**

Figure S2 Severity hierarchy of cardiovascular disease domains (Heart valve diseases)

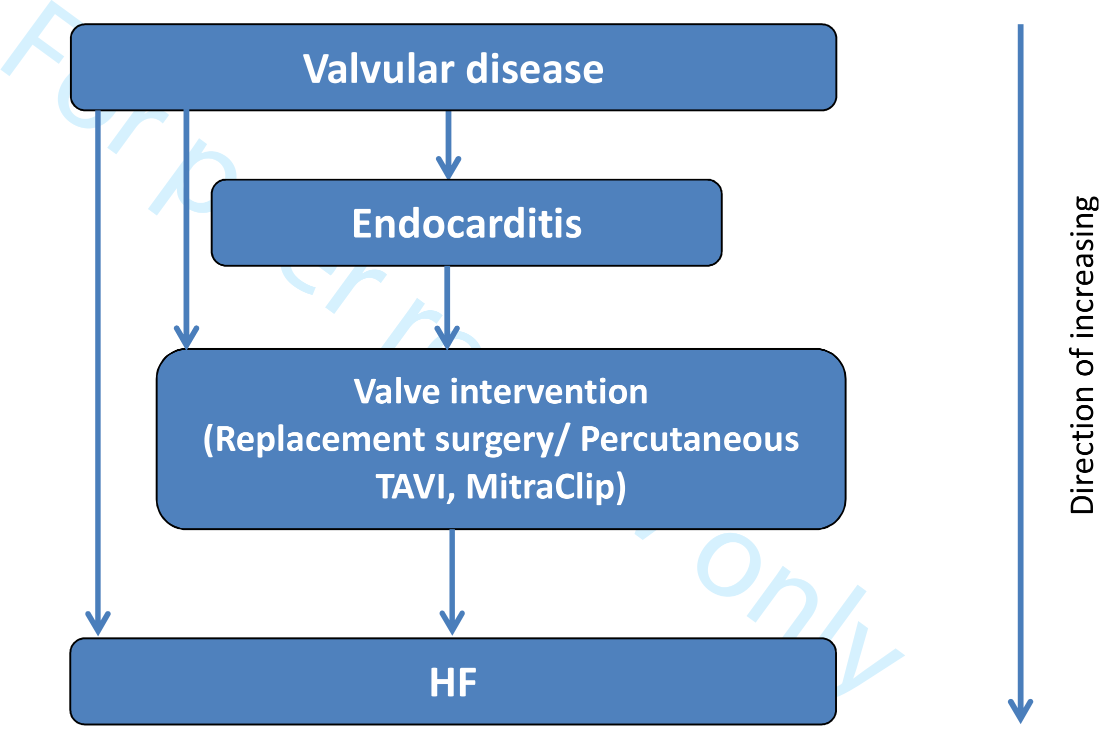

For peer review only - http://bmjopen.bmj.com/site/about/guidelines.xhtml

Supplementary material to protocol "**Protocol for a cohort study to quantify and stratify the severity of type 2 diabetes in primary care in England: rationale and study design" Zghebi et al. 2017**

Table S3 Factors used in previous studies to define severity of diabetes

Study ID	Factors used to define diabetes severity / Outcome(s)	Severity scale/ levels	Validation study
**Gini et al. (2016)[(1)]**	• Indication for insulin therapy<br>• The presence of diabetes-related complications<br><br>**Outcomes:**<br>Assessing the positive predictive value (PPV) for the presence/absence of type 2 diabetes against the GP's response to the questionnaire and measure the agreement on the severity level.	Four levels of T2DM severity:<br>• **Level 1**: Clinical definition of the disease, no indication for Insulin therapy and no complications listed in Level 3.<br>• **Level 2**: as in Level 1 except insulin is indicated.<br>• **Level 3**: as in Level 1 except with one of the following complications: (1) arterial stenosis, angina, MI, TIA, ischaemic stroke, intermittent claudication, diabetic foot ulcer, lower limb amputation, (2) retinopathy, (3) incipient or overt diabetic nephropathy/ dialysis.<br>• **Level 4**: As in Level 3 except insulin is indicated	A random sample of identified cases was validated by interviewing their self-selected general practitioners (GPs).
**Grootenhuis et al. (1994)[(2)]**	Type 2 diabetes symptom severity items categorised over six clinical dimensions:<br>• Hyperglycaemic<br>• Hypoglycaemic<br>• Psychological<br>• Cardiovascular<br>• Neuropathic<br>• Ophthalmological<br><br>**Outcomes:**<br>To measure differences in symptom severity between patients and detect changes over time within patients.	A 34-item patient-derived checklist used as a measure for type 2 diabetes symptom severity.	The face and content validity of severity items and dimension structures were based on literature and experiences of diabetologists.
**Young et al. (2008)[(3)]**	From laboratory data and diabetes-related complications:<br>• Retinopathy<br>• Nephropathy<br>• Neuropathy<br>• Cerebrovascular<br>• Cardiovascular<br>• Peripheral vascular disease<br>• Metabolic<br><br>**Outcomes:**<br>Risk of mortality, hospitalisation and healthcare utilisation.	Developed the Diabetes Complication Severity Index (DCSI) that is categorised to two or three levels (0=no abnormality; 1=some abnormality, 2=severe abnormality) depending on the severity of the complication. So if no abnormalities were present, the patient received no score, a 1 was added to the DCSI if patient had any complication classified as some abnormality and 2 was added if patient had any complication classified as severe abnormality.	The study design was a validation sample

For peer review only

For peer review only - http://bmjopen.bmj.com/site/about/guidelines.xhtml

Supplementary material to protocol "**Protocol for a cohort study to quantify and stratify the severity of type 2 diabetes in primary care in England: rationale and study design" Zghebi et al. 2017**

References

1. Gini R, Schuemie MJ, Mazzaglia G, Lapi F, Francesconi P, Pasqua A, et al. Automatic identification of type 2 diabetes, hypertension, ischaemic heart disease, heart failure and their levels of severity from Italian General Practitioners' electronic medical records: a validation study. BMJ open. 2016;6(12):e012413. Epub 2016/12/13.
2. Grootenhuis PA, Snoek FJ, Heine RJ, Bouter LM. Development of a type 2 diabetes symptom checklist: a measure of symptom severity. Diabetic medicine : a journal of the British Diabetic Association. 1994;11(3):253-61. Epub 1994/04/01.
3. Young BA, Lin E, Von Korff M, Simon G, Ciechanowski P, Ludman EJ, et al. Diabetes complications severity index and risk of mortality, hospitalization, and healthcare utilization. Am J Manag Care. 2008;14(1):15-24.

For peer review only

BMJ Open

Using electronic health records to quantify and stratify the severity of type 2 diabetes in primary care in England: rationale and cohort study design

Journal:	*BMJ Open*
Manuscript ID	bmjopen-2017-020926.R1
Article Type:	Protocol
Date Submitted by the Author:	26-Jan-2018

Complete List of Authors:	Zghebi, Salwa; University of Manchester, NIHR School for Primary Care Research, Centre for Primary Care<br>Rutter, Martin; University of Manchester Faculty of Biology, Medicine and Health, Division of Diabetes, Endocrinology and Gastroenterology; Manchester University NHS Foundation Trust, Manchester Diabetes Centre<br>Ashcroft, Darren; University of Manchester, Centre for Pharmacoepidemiology and Drug Safety,<br>Salisbury, Chris; University of Bristol, Academic Unit of Primary Health Care<br>Mallen, Christian; Keele University, Arthritis Research UK Primary Care Centre<br>Chew-Graham, Carolyn; University of Keele<br>Reeves, David; University of Manchester, Institute of Population Health; Centre for Biostatistics<br>van Marwijk, Harm; University of Brighton<br>Qureshi, Nadeem; University of Nottingham, School of Medicine<br>Weng, Stephen; University of Nottingham, NIHR School of Primary Care Research<br>Peek, N; Health e-Research Centre, Institute of Population Health, University of Manchester<br>Planner, Claire; NIHR School for Primary Care Research, Centre for Primary Care, Institute of Population Health<br>Nowakowska, Magdalena ; University of Manchester, NIHR School for Primary Care Research, Centre for Primary Care<br>Mamas, Mamas; Keele Cardiovascular Research group, Centre for Prognosis Research<br>Kontopantelis, Evangelos; University of Manchester, Faculty of Biology, Medicine and Health; NIHR School for Primary Care Research, Centre for Primary Care

**Primary Subject Heading**:	Diabetes and endocrinology
Secondary Subject Heading:	General practice / Family practice, Health services research
Keywords:	Type 2 diabetes, Electronic Health Records, PRIMARY CARE, Hospitalisation records, Cardiovascular disease, Diabetes Severity Algorithm

For peer review only

Using electronic health records to quantify and stratify the severity of type 2 diabetes in primary care in England: rationale and cohort study design

Salwa S Zghebi,[1] Martin K Rutter,[2,3] Darren M Ashcroft,[4] Chris Salisbury,[5] Christian Mallen,[6] Carolyn A. Chew-Graham,[6] David Reeves,[1] Harm Van marwijk,[7] Nadeem Qureshi,[8] Stephen Weng,[8] Niels Peek,[9] Claire Planner,[1] Magdalena Nowakowska,[1] Mamas Mamas,[10] Evangelos Kontopantelis[1,9]

1. Centre for Primary Care, School of Health Sciences, Faculty of Biology, Medicine and Health, Health Innovation Manchester, University of Manchester, Manchester, UK.

2. Division of Diabetes, Endocrinology and Gastroenterology, School of Medical Sciences, Faculty of Biology, Medicine and Health, University of Manchester, Manchester, M13 9PL, UK.

3. Manchester Diabetes Centre, 193 Hathersage Rd, Manchester University NHS Foundation Trust, Manchester Academic Health Science Centre,, Manchester, M13 0JE, UK.

4. Centre for Pharmacoepidemiology and Drug Safety, School of Health Sciences, Faculty of Biology, Medicine and Health, Health Innovation Manchester, University of Manchester, Manchester, UK.

5. Centre for Academic Primary Care, Population Health Sciences, Bristol Medical School, University of Bristol, Bristol, UK.

6. Research Institute for Primary Care and Health Sciences, Keele University, Staffordshire, UK.

7. Division of Primary Care and Public Health, Brighton and Sussex Medical School, University of Brighton, Brighton, UK.

8. School of Medicine, University of Nottingham, Nottingham, UK.

9. Division of Informatics, School of Health Sciences, Faculty of Biology, Medicine and Health, Imaging & Data Sciences (L5), Health Innovation Manchester, University of Manchester, Manchester, UK.

10. Keele Cardiovascular Research group, Centre for Prognosis Research, Institute for Primary Care and Health Sciences, Stoke-on-Trent, UK.

Corresponding author. Salwa Zghebi. Centre for Primary Care, University of Manchester, Manchester, UK. Email address: Salwa.Zghebi@manchester.ac.uk

Number of words (Abstract): 300
Number of words (full text): 3,948
Number of Tables 1
Number of Figures: 4

For peer review only - http://bmjopen.bmj.com/site/about/guidelines.xhtml

Abstract

Introduction: The increasing prevalence of type 2 diabetes (T2DM) presents a significant burden on affected individuals and health-care systems internationally. There is, however, no agreed validated measure to infer diabetes severity from electronic health records (EHRs). We aim to quantify T2DM severity and validate it using clinical adverse outcomes.

Methods and Analysis: Primary care data from the Clinical Practice Research Datalink (CPRD), linked hospitalisation and mortality records between January-2006 and December-2016 for T2DM patients in England will be used to develop a clinical algorithm to grade T2DM severity. The EHR-based algorithm will incorporate main risk factors (severity domains) for adverse outcomes to stratify T2DM cohorts by baseline and longitudinal severity scores. Provisionally, T2DM severity domains, identified through a systematic review and expert opinion are: diabetes duration, HbA_{1c}, microvascular complications, comorbidities, and co-prescribed treatments. Severity scores will be developed by two approaches: i) calculating an aggregate score of severity domains; ii) through hierarchical stratification of complications. Experts by experience will agree on domains before finalising the algorithm. Survival analysis for the association between measured severity scores and future outcomes: cardiovascular events; hospitalisation (diabetes-related, cardiovascular); and mortality (diabetes-related, cardiovascular, all-cause mortality) will be performed as a statistical validation. The proposed EHR-based approach will quantify the T2DM severity for primary care performance management and inform the methodology for measuring severity of other primary care-managed chronic conditions. We anticipate that the developed algorithm will be a practical tool for practitioners, aid clinical management decision-making, inform stratified medicine, support future clinical trials and contribute to more effective service-planning and policy-making.

Ethics and Dissemination: The study protocol was approved by the Independent Scientific Advisory Committee (ISAC) (Reference:17_168R, September-2017). Some data were presented at the NIHR School for Primary Care Research Showcase, September-2017, Oxford,UK. The study findings will be disseminated in relevant academic conferences and peer-reviewed journals.

For peer review only - http://bmjopen.bmj.com/site/about/guidelines.xhtml

Strengths and limitations of this study

- This is the first UK-based study to develop a diabetes severity scoring tool based on real-world electronic healthcare data to grade people with type 2 diabetes by their clinical severity.
- The study will include a large sample size and use high-quality medical data routinely-collected from general practices with access to linked national hospitalisation and cause-specific mortality datasets.
- The association between the computed severity scores and future adverse outcomes (cardiovascular event, hospitalisation and mortality) will be used to validate the developed severity algorithm.
- There is a possibility to miss other severity indicators not recorded in the data sources used such as pharmacy dispensing data.
- Given that the linkage scheme is only available for consented English general practices, the study cohort will be restricted to eligible patients registered within England (form nearly 58% of total CPRD general practices).

For peer review only

For peer review only - http://bmjopen.bmj.com/site/about/guidelines.xhtml

Introduction

The worldwide prevalence of diabetes mellitus is increasing with World Health Organisation (WHO) estimating that over 422 million adults had diabetes in 2016 with a growth in the global prevalence from 4.7% in 1980 to 8.5% in 2014.[(1,2)] In the UK, the prevalence of type 2 diabetes (T2DM) has nearly doubled between 2004 and 2014.[(3,4)] People with diabetes have a higher risk for morbidity and mortality when compared to individuals without diabetes,[(3,5)] and hence the increasing prevalence presents a significant burden on health care resources. Diabetes management expenses are estimated to consume up to 11% of the total health care budget in the UK and USA.[(6,7)] Diabetes is a complex metabolic condition, of an increasing severity,[(8,9)] at individually varying levels, and progressive development of vascular complications and end organ damage over time. Diabetes is mainly managed in primary care in the UK,[(10)] overall Europe,[(11)] USA,[(12)] and Asia.[(13,14)] In the UK, nearly 75% of the diabetes-associated costs relate to management of diabetic complications.[(6)]

The 'severity' of clinical conditions can be conceptualised as a progression of the underlying disease process. Increasing disease severity and development of associated complications lead to greater treatment complexity and clinical impact. The severity of chronic conditions, such as diabetes, has not been widely considered despite the clinical relevance and its likely impact on health care systems, where few studies have assessed disease severity among patients with T2DM or have quantified temporal trends or scores of severity.[(15,16)]

Severity scores could be clinically useful, particularly in T2DM, as such a new summary score could contribute above what existing risk scores offer (e.g. moving beyond risk for death) and also be highly relevant for use in six main clinical and research areas: i) identifying complex patients with a higher need for future care; ii) identifying patients at early stages of disease (benchmarking); iii) providing information that will directly inform clinical care; iv) identifying trajectories in severity over time; v) supporting research, for example, by serving as an important diabetes-specific covariate to consider in analyses, similarly to the Charlson Comorbidity Index, or as an outcome; and vi) providing data that will inform resource allocation in health systems, such as the National Health Service (NHS).

For peer review only - http://bmjopen.bmj.com/site/about/guidelines.xhtml

Here, we describe the conceptual development of a T2DM severity algorithm model, using electronic health records (EHRs) on clinical consultations and treatments. Our aim is to use routinely-collected clinical and administrative data to develop a scoring tool which would quantify and grade the severity of T2DM. This model could potentially be used by clinicians to stratify patients based on disease severity and we aim to demonstrate potential advantages over similar risk scores. To validate the algorithm, our secondary aim is to examine the association of severity grades with risk of three main adverse outcomes: cardiovascular disease, hospitalisation, and mortality. The developed severity stratification algorithm is anticipated to have direct impact on clinical practice and have wider implications on service-planning and policy-making.

Methods and Analysis

Data Source

This study will use data from the Clinical Practice Research Datalink (CPRD). The CPRD is one of the world's largest EHR with anonymised primary care records from over 650 general practices over the UK.[(17)] Nearly 76% of all CPRD registered patients are in England.[(17)] CPRD data include clinical diagnoses, prescribed therapies, biochemical test results and referrals to healthcare services. The accuracy and completeness of diagnostic coding and validity of CPRD data, in clinical research, have been reported as excellent with positive predictive values (PPVs) of over 90% for nearly 14 conditions (including diabetes and cerebrovascular disease).[(18)] PPVs were defined as the proportion of CPRD diagnoses that were validated as true cases when compared to a gold standard such as GP questionnaire or primary care records.

Two metrics for research-quality data, recommended to be used by researchers, are available for CPRD records.[(17)] For patient-level data, the flag 'acceptable' indicates that a patient's record has met certain quality standards such as registration status, valid age and gender, and record of patient events. For practice-level data, the up-to-standard (UTS) date is used as a data quality measure. The UTS date is calculated for each CPRD general practice as the latest date at which the general practice meets minimum quality criteria based on

For peer review only - http://bmjopen.bmj.com/site/about/guidelines.xhtml

two central concepts: the continuity of recorded data and the number of recorded deaths, in comparison to an expected national range.[17,19]

Nearly 75% of general practices in England (approximately 58% of all CPRD practices) have consented to the CPRD Linkage Scheme for access to a number of linked datasets and national disease registries.[17] These include hospital data (including outpatient, admissions, and Accident and Emergency (A&E) data), mortality records (held by the Office for National Statistics (ONS)), socio-economic status and the cancer registry. The linked hospitalisation records, held by the Hospital Episode Statistics (HES), provide ethnicity data, admission and discharge dates, clinical diagnoses and procedures during hospitalisation recorded using the tenth revision of the International Classification of Diseases (ICD-10) and operating procedure codes (OPCS). This study will only include patients registered with English general practices which provide up-to-standard data and which participate in the CPRD Linkage Scheme.

Study Population

The study cohort will include individuals aged 35 years or over with T2DM (with ≥1 diagnostic code as in Table S1) between 1st January 2006 and 31st December 2016. Patients with an ever record of type 1 diabetes (with ever code for type 1 diabetes as in Table S2) will be excluded unless have records of non-insulin antidiabetic therapies. We chose the study period from 2006 as the quality of CPRD data has improved substantially to adhere with the then introduced important changes to the national incentive scheme The Quality and Outcomes Framework (QOF), an incentivisation programme for all GP surgeries in the UK.[20,21]

For this cohort construction, we will consider implementing previously-validated algorithms designed to identify diabetes cases by avoiding potential misclassification in routinely-collected data such as CPRD.[22,23] Eligible patients will be followed-up until censored at the earliest instance of any of the following event dates: patient transferred out of the practice (any cause), last collection date for the practice, the study end on the 31st December 2016 or death. The main demographic and clinical characteristics of the defined diabetes cohorts

For peer review only - http://bmjopen.bmj.com/site/about/guidelines.xhtml

(such as age, gender, geographic region within England, patient-level and general practice-level social deprivation, BMI, and baseline HbA_{1c}) will be identified. Based on our previous studies, the expected sample size in a year will include 11,000 T2DM patients registered with English general practices linked to the HES Admitted Patient Care dataset.

Severity Domains

A systematic literature search for studies that developed algorithms or models to assess and quantify the severity of diabetes was conducted to identify the domains and sub-domains for T2DM severity (to be published separately). Also, expert clinical opinion from members of the research team was used to supplement the search process and identify possible omissions. Clinical members in the team include pharmacists (DMA, SSZ), general practitioners (CS, CM, CCG, HVm, and NQ), a consultant diabetologist (MKR) and a consultant cardiologist (MM), who used their expertise to create a list of relevant clinical domains for T2DM severity. The final domains to be included in the severity model will be decided during the analysis stage. Currently, the identified clinical domains that are relevant to the degree of progression of T2DM include: patient factors (diabetes duration (the period between T2DM diagnosis and the severity score estimation) and body mass index (BMI)), monitoring laboratory tests (glycated haemoglobin (HbA_{1c}) categories (threshold of 7% [53 mmol/mol]), blood glucose levels), type of antidiabetic therapy, other prescribed medications (such as lipid-regulating medications and angiotensin-converting enzyme inhibitors (ACEIs)), comorbidities (including diabetes-related complications, depression), hospitalisation, and surgical interventions. Comorbidities will be identified using appropriate code lists and contribute to the severity score according to each score computing method. The identified domains and sub-domains are described in Table 1. Domains will also be reviewed by a panel of 'experts by experience' (people with lived experience of T2DM) to provide patient validation of the severity scoring tool.

The clinical Read codes for the defined severity domains codes and the product codes for drug therapies will be identified using the [pcdsearch] Stata user command.[24] The [pcdsearch] command is a search programme developed to extract code lists from typically

For peer review only - http://bmjopen.bmj.com/site/about/guidelines.xhtml

very long lookup files associated with primary care databases using an input file containing a list of stubs for codes of interest to be searched for. For CPRD, the lookup files Medical dataset is searched for all clinical Read codes and the Product lookup file, that includes unique product codes, is searched for all treatments. The corresponding ICD-10 codes will be used to identify clinical domains recorded in the hospitalisation data and ONS mortality records. The relevant CPRD operational identification entities for the laboratory tests will be identified. This is an ongoing process whereby the final lists of domains and codes will be reached by consensus among the clinical members of the team. All code lists will be available on the online clinical code repository (ClinicalCodes.org).[25]

Study Outcomes

The adverse outcomes of interest will be the development of the first event among cardiovascular disease, (myocardial infarction, stroke), future hospitalisation (any hospitalisation, diabetes-related and cardiovascular hospitalisation) and death (diabetes-related mortality, cardiovascular mortality and all-cause mortality). Secondary outcome will be future hospitalisation due to hypoglycaemia, a relevant and potentially preventable adverse outcome. The hospitalisation and death outcomes will be identified using the linked HES and ONS mortality data, respectively. Similarly with severity domains, Read codes and ICD-10 codes (available on ClinicalCodes.org online repository) will be used to identify the outcomes as appropriate.

Statistical Analyses

Diabetes Severity Algorithm

Using annual data bins and grouping diabetes patients from 1st January to 31st December in a calendar year between 2006 to 2016, the developed diabetes algorithm will grade the severity of T2DM using pre-defined (sub)domains. Our study period (2006 onwards) was selected to ensure very high data quality in primary care, while the addition of secondary

For peer review only - http://bmjopen.bmj.com/site/about/guidelines.xhtml

care data will make our analyses even more robust, in terms of accurately classifying T2DM severity levels.

We will consider two approaches to derive numerical or categorical diabetes severity scores or levels. Firstly, we will use a binary classification (severity indicator: present/absent) within each sub-domain and calculate an aggregate score. The second approach, through hierarchical stratification of end organ microvascular and macrovascular complications, will involve increasing weighting within each sub-domain, as severity increases where scores of 1, 2, or 3 on each subdomain will be assigned based on clinical input in terms of severity. Then, we will use the model estimates to weight sub-domains (according to weights calculated from t-values of the fitted regression model). The highest possible severity score will be known when the final list of included domains and sub-domains is decided. Hierarchical diagrams of the clinical severity of identified diabetes-related complications domains (Figure 1) cerebrovascular (Figure 2), and cardiovascular (Figure 3, Figure 4, Figure S1, Figure S2) domains are presented.

Sub-domains with many measurements over time, such as HbA_{1c} levels and BMI, will be modelled as time-varying covariates (alternative models will be considered to account for non-linear changes). Within each time window the average of these measurements will be used, and we will also use multiple imputation approaches to account for missing data. Provisionally, we will consider using data records within look-back periods between 3-5 years. Different look-back windows within this period will be tested to obtain the optimal time window. Descriptive statistics of the study population and the estimated scores will be performed.

Severity Algorithm Validation

When we have developed a first version of the severity score tool, it will be validated statistically by quantifying the association of severity scores to future adverse outcomes. The primary outcome will be developing a cardiovascular event (myocardial infarction, stroke), future hospitalisation and death (diabetes-related mortality, cardiovascular mortality and all-cause mortality). A secondary adverse outcome will be future hospital

For peer review only - http://bmjopen.bmj.com/site/about/guidelines.xhtml

admission due to hypoglycaemia, a relevant and potentially preventable adverse outcome. Cox proportional hazards (after assessing proportional hazards assumption) and/or competing-risks regressions will be used to perform survival analyses and assess the relationship between the severity score and outcomes controlling for all available patient characteristics (such as age, gender, ethnicity, and level of social deprivation). We aim to analyse and assess how severity scores differ across different levels of these variables. The cohort will be randomly split into a training (90%) and validation dataset (10%), and the latter will be used to validate the performance of the severity algorithm. The Cox regression analysis will form the basis of the validation for the developed severity algorithm and we will assess the model performance using relevant measures (such as area under the receiver operating characteristic curve (AUROC). Using this analysis, we will also test what how much the 'sophisticated' full model that includes all relevant severity domains will add in terms of predictive validity of the adverse outcomes over a simpler model that includes demographic data (age, gender and level of social deprivation) alone.

Longitudinal Trends of Diabetes Severity

In newly diagnosed T2DM patients during the study period we will assess the temporal trends of severity scores over time by calculating the time needed to progress along the quintiles of the severity scale starting from the date or year of diagnosis and identify the predictors to this progression across quintiles.

While the inclusion of some domains such as carotid artery stenting, the use of ACEIs or lipid-regulating drugs, kidney transplantation and laser therapy are clinically-relevant and represent markers of greater level of disease severity they also improve the prognosis (lessen the grade of severity) i.e. raising a risk of "feedback". While we will further assess their role, we may have to not include these domains in the modelling at the analysis stage. All statistical analyses will be conducted using Stata Statistical Software for Windows: *Release 15* (College Station, TX: StataCorp LP, USA).

For peer review only - http://bmjopen.bmj.com/site/about/guidelines.xhtml

Patient and Public Involvement and Engagement (PPIE)

The public interest in the reuse of routinely-collected electronic health data for research purposes has expanded over recent years. Our study presents a valuable opportunity to address the challenge of how to develop meaningful and productive PPIE collaborations in observational studies that make use of secondary data.

We will identify suitable PPIE partners to collaborate on this study and develop creative strategies for meaningful involvement as the project develops. Participants to provide their input that will be particularly valuable in providing feedback into the diabetes severity algorithms, and deciding what information would be most useful to patients and how to be presented.

In the latter stages of the study, jointly with our PPIE partners, we will plan and deliver a patients' and carers' workshop where we will present emerging findings and seek feedback to inform further work and the dissemination strategy. This will include an exercise around weights of domains within the algorithm. PPIE work will be reported using the GRIPP (Guidance for Reporting Involvement of Patients and Public) checklist.[26]

Discussion

The global and country-specific prevalence of diabetes has increased substantially.[2,27-30] For T2DM, prevalence rates have doubled over the past two decades.[4,31,32] Diabetes diagnosis is associated with higher risk for morbidity and mortality in comparison to people without diabetes.[5] Despite the natural progression of most chronic diseases, including T2DM, and their important clinical implication on prognosis and medical resources utilisation, only a few studies have attempted to grade the severity of chronic conditions over time. Our proposed study aims to develop a contemporary algorithm to quantify the severity of T2DM, a highly prevalent condition, using routine EHR. The scoring tool will be based on clinically-relevant diagnoses and treatments modelled as severity domains and sub-domains. The algorithm will enable clinicians to grade T2DM patients according to the

For peer review only - http://bmjopen.bmj.com/site/about/guidelines.xhtml

level of their disease severity, at baseline and longitudinally, as driven by the weights of the included clinical domains.

Prior Studies

Currently, there is limited data around validated severity measures that can be used in routine clinical practice managing patients with T2DM. Consequently, we are in parallel finalising a conducted systematic review for clinical-based diabetes severity models which will inform the refinement of this work. Here, we discuss three relevant diabetes severity measures that have been previously reported from countries outside the UK.

In the first study, Gini et al. (2016) categorised the severity of T2DM (N=300) into four levels based on insulin use and the presence of diabetic complications (Table S3).[15] A validation study in a random sample of cases was performed by interviewing their general practitioners. We however, aim to include more T2DM patients and consider more clinical data such as other coexisting conditions, non-insulin therapies, statins and ACEIs, that have been shown to be associated with reduced risk of adverse outcomes.

In the second study, a diabetes symptom checklist was created to measure the perceived symptom severity and assess the changes over time in 185 T2DM patients.[16] The checklist contained 34 items categorised by main clinical symptoms (Table S3). The patterns of comorbidities and prescribed treatments were reportedly associated with significant differences in the estimated severity scores.[16] The sample size was however relatively small and the checklist was based on patients' perception on diabetes symptoms severity.

Thirdly, Young et al. (2008), calculated a diabetes complications severity index (DCSI) in 4,229 patients with type 1 or type 2 diabetes in one US geographic region to examine its association with adverse outcomes (risk of hospitalisation and mortality). In comparison to using a simple numerical count of complications, DCSI found to be a better tool to predict adverse outcomes.[33] The authors used pharmacy (insulin use only) and laboratory data to compute the severity index and included patients from clinics with largest ethnic diversity. However, the DCSI missed additional domains such as diabetes duration, hyperlipidaemia and a wider range of diabetes-related and end-organ damage manifestations that we aim to assess in addition to hospitalisation and mortality.

For peer review only - http://bmjopen.bmj.com/site/about/guidelines.xhtml

In the wider literature, although several studies have investigated the possible role of other various factors on the severity of diabetes, none have provided a severity scoring tool that utilises data from various clinical domains as planned in our study that can use the wealth of information routinely-collected in electronic healthcare databases. In these studies, approaches used to define diabetes severity included: comparing T2DM severity, before and after obesity surgery;[(34)] examining the association between diabetes severity and either: haematological and immunological changes,[(35)] levels of urine citrate,[(36)] a biomarker for adverse outcomes;[(37)] grip strength;[(38)] or the use of complementary medicine to manage T2DM.[(39)]

Overall, the severity of T2DM has been assessed using the following domains: the complexity of antidiabetic treatment regimens[(34)] or HbA_{1c} levels;[(36)] a summary severity variable that includes vascular complications;[(40)] or a health status composite, number of comorbidities and patterns of treatments.[(41)] Other putative and less clinically-robust indicators or animal models were reportedly used to assess the severity of T2DM such as the effect of different patient education approaches on diabetes severity;[(42)] evaluating the effect of parental history;[(43)] and the role of genetic,[(44,45)] metabolic,[(46)] or inflammatory mediators.[(47)]

In comparison to our planned study, none of previous studies included as many routinely-collected clinically-relevant variables as in our more inclusive summary severity score algorithm or assessed the association of estimated scores with the various adverse outcomes included in our defined primary and secondary endpoints.

Potential Strengths and Limitations

Our proposed study has several potential strengths: First, it aims to present a contemporary measure to the available tools and the first UK-based study to develop an EHR-based severity scoring tool to grade patients by their T2DM severity. Second, the study uses high-quality real-world medical data routinely-collected from general practices. The use of routinely-collected data indicates that severity scores can be generated automatically with minimal effort. Third, the views of PPIE collaborators will be incorporated in the

For peer review only - http://bmjopen.bmj.com/site/about/guidelines.xhtml

development of the severity tool. Fourth, we will access two linked datasets: hospitalisation (HES) data (to maximise data capture and reduce condition misclassification), and cause-specific mortality data to ascertain causes of death. Fifth, the sample size is expected to be large enough to drive the development and evaluation of the severity algorithm. Sixth, a statistical validation of the developed algorithm is planned and described.

As we will use data available in CPRD and HES datasets, one of the limitations we anticipate is the possibility to miss other severity indicators not recorded in used datasets. These include detailed pharmacy data such as 'actual' dispensing and adherence data. Also, the use of routinely-collected data is associated with missing values, being collected from questionnaires, and issues around the accuracy of coding. However, we plan to use appropriate imputation methods and definite criteria to minimise the effect of coding issues. In addition, underestimating T2DM severity levels for patients not regularly attending a general practice or not being reliably captured in the database due to very high mobility (e.g. homeless patients), is a possibility and should be acknowledged. Another limitation is that our study will be restricted to patients registered with general practices in England, as CPRD currently only provides linkage to external national datasets for consented general practices in England. However, English general practices form the majority of all CPRD practices including nearly 76% of total registered patients.[17] Finally, due to project time constrains, there is no possibility to validate the developed diabetes scores using questionnaires (as reported previously [15]) or replicating the algorithm in a separate dataset. However, we have planned a statistical validation of the developed algorithm.

Importance and the Clinical Implications of the Severity Tool

The developed algorithm and severity tool may have significant implications for primary care both in terms of disease management and resource allocation. Ideally, through future work of further validation and assessment of clinical utility, the severity tool will be of practical use in primary care through its implementation in the clinical computing systems used in the UK.[48] The clinical significance of the developed severity algorithm to primary

For peer review only - http://bmjopen.bmj.com/site/about/guidelines.xhtml

care is driven by the inclusion of highly-relevant clinical domains, such as diabetes-related complications and comorbidities, mapped to routinely-collected data.

Additionally, by assessing the longitudinal patterns of severity, the developed tool may be more clinically relevant than the currently used proxy (HbA_{1c}), and thus it could be a more reliable indicator in informing practices' remuneration for diabetes care. Categorising individuals based on their diabetes severity will be relevant for risk stratification (which may enable safer delegation of care within the clinical team), identify individualised patient risks and will help practitioners triage patients in need of a greater clinical input which informs towards stratified medicine to reduce future life-changing diabetes-related complications. Moreover, the weights of the severity scores may inform future clinical trials as the scoring tool considers a broader range of cardiovascular conditions than in most randomised clinical trials. Given the relatively low rates of cardiovascular outcomes in some trials, identifying diabetes patients who are at higher risk via our severity algorithm would help to power trials with overall longer term benefit for patients. Finally, the composite severity score may serve as an important confounding factor in future research, as for example, to match diabetes cases and controls in observational studies and clinical trials.

Conclusions

Despite the availability of rich routinely-collected electronic medical data, there are limited validated measures to quantify the severity of main chronic conditions managed in primary care utilising this important resource. We aim to develop a severity algorithm for type 2 diabetes to quantify disease severity using clinically-important domains derived from UK EHR. Our goal is that developed severity score data will provide clinicians with practical information that will influence management pathways. The findings could have important implications for service-planning and policy-making. The severity tool may provide a useful model and methodology towards the assessment of severity in other chronic medical conditions managed in primary care setting. Future research may involve further validation by testing the severity model in a separate primary care database.

For peer review only - http://bmjopen.bmj.com/site/about/guidelines.xhtml

Author Contributions

E.K. S.S.Z. M.M., M.K.R and H.V.m developed the study design and data analysis plan. S.S.Z. M.M., M.K.R and H.V.m agreed on provisional clinical code lists. S.S.Z. prepared the first draft of the manuscript, and E.K., M.M., M.K.R. and H.V.m critically reviewed initial versions. C.P. contributed to the planned PPIE work. D.R., C.C.G., C.M., N.P., D.M.A., C.S., N.Q., M.N. and S.W reviewed and critically edited the manuscript. All authors approved the final version of the protocol before submission. S.S.Z. is the guarantor.

Funding

This study is funded by the National Institute for Health Research School for Primary Care Research (NIHR SPCR), grant number 331. This report is independent research by the National Institute for Health Research. The views expressed in this publication are those of the authors and necessarily those of the NHS, the National Institute for Health Research or the Department of Health.

Competing Interests

E.K., H.V.m, M.M., D.R., C.C.G. C.P., C.M., N.P., M.N. declare no competing interests. S.S.Z. reports support by the NIHR SPCR during this study. D.M.A. has received grant funding from Abbvie and has served on advisory boards for Pfizer and GSK. M.K.R. has received educational grant support from MSD and Novo Nordisk; has modest stock ownership in GSK; and has consulted for Roche. N.Q. reports grants from the NIHR SPCR, during the conduct of the study. C.S. reports grants from NIHR SPCR, during the conduct of the study; grants from NHS CLAHRC West, grants from Avon Primary Care Research Collaborative, outside the submitted work. S.W. serves as a member of the Clinical Practice Research Datalink Independent Scientific Committee (ISAC) at the UK Medicines and Health Regulatory Agency.

For peer review only

For peer review only - http://bmjopen.bmj.com/site/about/guidelines.xhtml

Ethical Approval

The approval for the study protocol was obtained from the CPRD's Independent Scientific Advisory Committee (ISAC).

List of Abbreviations

ACEI: Angiotensin converting enzyme inhibitors; **CABG**: coronary artery bypass graft; **CHD**: coronary heart disease; **CKD**: chronic kidney disease; **CPRD**: Clinical Practice Research Datalink; **CRT**: cardiac resynchronization therapy; **DCSI**: diabetes complications severity index; **EHR**: electronic health record; **EVAR**: Endovascular aneurysm repair; **GRIPP**: Guidance for Reporting Involvement of Patients and Public; **HbA_{1c}**: glycated haemoglobin; **HES**: Hospital Episode Statistics; **HES A&E:** HES Accident & Emergency; **HF**: heart failure; **ICD**: implantable cardioverter defibrillator; **ICD-10**: the 10th revision of the International Classification of Diseases; **ISAC**: Independent Scientific Advisory Committee; **MI**: myocardial infarction; **NHS:** National Health Service; **ONS**: Office for National Statistics; **OPCS**: operating procedure codes; **PAD**: peripheral artery disease; **PCI**: percutaneous coronary intervention; **PPIE:** patient and public involvement and engagement; **PTCA**: percutaneous transluminal coronary angioplasty; **PVD**: peripheral vascular disease; **QOF**: Quality and Outcomes Framework; **SVT**: supraventricular tachycardia; **TAVI**: transcatheter aortic valve implantation; **T2DM**: type 2 diabetes; **TIA**: transient ischemic attack; **VF**: ventricular fibrillation; **VT**: ventricular tachycardia; **WHO**: World Health Organization.

For peer review only

References

1. World Health Organization (WHO) media centre. Diabetes. [Fact sheet] 2016 [cited 2016 29 April]; Available from: http://www.who.int/mediacentre/factsheets/fs312/en/.

2. World Health Organization (WHO). Global Report on Diabetes. 2016 [cited 2017 11 February]; Available from: http://apps.who.int/iris/bitstream/10665/204871/1/9789241565257_eng.pdf.

3. Zghebi SS, Steinke DT, Carr MJ, Rutter MK, Emsley RA, Ashcroft DM. Examining Trends in Type 2 Diabetes Incidence, Prevalence and Mortality in the UK between 2004 and 2014. Diabetes, Obesity and Metabolism. 2017.

4. Sharma M, Nazareth I, Petersen I. Trends in incidence, prevalence and prescribing in type 2 diabetes mellitus between 2000 and 2013 in primary care: a retrospective cohort study. BMJ Open. 2016;6:e010210.

5. American Diabetes Association (ADA). Standards of Medical Care in Diabetes - 2015. Diabetes Care. 2015;38(Suppl 1):S1-S49.

6. Hex N, Bartlett C, Wright D, Taylor M, Varley D. Estimating the current and future costs of Type 1 and Type 2 diabetes in the UK, including direct health costs and indirect societal and productivity costs. Diabetic Medicine. 2012;29:855-62.

7. Walker BR, Colledge NR, Ralston SH, Penman ID. Davidson's Principles and Practice of Medicine. China: Elsevier 2014 [cited 2016 30 June]. Available from: http://www.elsevier-etextbooks.com/pdfreader/davidsons-principles-practice-medicine57814.

8. Walker BR, Colledge NR, Ralston SH, Penman ID. Davidson's Principles and Practice of Medicine: Elsevier; 2014 [cited 2017 11 May]. Available from: http://elsevierelibrary.co.uk/pdfreader/davidsons-principles-practice-medicine57814.

9. Nefs G, Pop VJM, Denollet J, Pouwer F. The Longitudinal Association between Depressive Symptoms and Initiation of Insulin Therapy in People with Type 2 Diabetes in Primary Care. Plos One. 2013;8(11).

10. Murrells T, Ball J, Cookson G, Maben J, Lee G, Griffiths P. Managing diabetes in primary care: how does the configuration of the workforce affect quality of care? National Nursing Research Unit, King's College London, 2013 November 2013. Report No.: Department of Health Policy Research Programme, ref. 016/0058.

11. Luk VE, Patricia S, Luc F, Liesbeth B, Jan DM. Priorities for diabetes primary care in Europe. Primary Care Diabetes. 2008;2(1):3-8.

12. Spann SJ, Nutting PA, Galliher JM, Peterson KA, Pavlik VN, Dickinson LM, et al. Management of Type 2 Diabetes in the Primary Care Setting: A Practice-Based Research Network Study. Ann Fam Med. 2006;4(1):23–31.

13. Tai T-Y, Chuang L-M, Tsai S-T, Huang B-Y, The Diabcare (Taiwan) Study Group. Treatment of Type 2 Diabetes Mellitus in a Primary Care Setting in Taiwan: Comparison with Secondary/Tertiary Care. Journal of the Formosan Medical Association = Taiwan yi zhi. 2006;105(2):105–17.

14. Mafauzy M. Diabetes control and complications in private primary healthcare in Malaysia. Med J Malaysia. 2005;60(2):212-7.

15. Gini R, Schuemie MJ, Mazzaglia G, Lapi F, Francesconi P, Pasqua A, et al. Automatic identification of type 2 diabetes, hypertension, ischaemic heart disease, heart failure and their levels of severity from Italian General Practitioners' electronic medical records: a validation study. BMJ open. 2016;6(12):e012413. Epub 2016/12/13.

16. Grootenhuis PA, Snoek FJ, Heine RJ, Bouter LM. Development of a type 2 diabetes symptom checklist: a measure of symptom severity. Diabetic medicine : a journal of the British Diabetic Association. 1994;11(3):253-61. Epub 1994/04/01.

17. Herrett E, Gallagher AM, Bhaskaran K, Forbes H, Mathur R, van Staa TP, et al. Data Resource Profile: Clinical Practice Research Datalink (CPRD). International Journal of Epidemiology. 2015;44(3):827-36.

For peer review only - http://bmjopen.bmj.com/site/about/guidelines.xhtml

18. Khan NF, Harrison SE, Rose PW. Validity of diagnostic coding within the General Practice Research Database: a systematic review. British Journal of General Practice. 2010;60(572):e128-e36.
19. Williams T, van Staa T, Puri S, Eaton S. Recent advances in the utility and use of the General Practice Research Database as an example of a UK Primary Care Data resource. Therapeutic Advances in Drug Safety. 2012;3(2):89-99.
20. Lester H, Campbell S. Developing Quality and Outcomes Framework (QOF) indicators and the concept of 'QOFability'. Quality in Primary Care. 2010;18:103-9.
21. Calvert M, Shankar A, McManus RJ, Lester H, Freemantle N. Effect of the quality and outcomes framework on diabetes care in the United Kingdom: retrospective cohort study. BMJ. 2009;338:b1870.
22. de Lusignan S, Khunti K, Belsey J, Hattersley A, van Vlymen J, Gallagher H, et al. A method of identifying and correcting miscoding, misclassification and misdiagnosis in diabetes: a pilot and validation study of routinely collected data. Diabetic Medicine. 2010;27(2):203-9.
23. Wright AK, Kontopantelis E, Emsley R, Buchan I, Sattar N, Rutter MK, et al. Life Expectancy and Cause-Specific Mortality in Type 2 Diabetes: A Population-Based Cohort Study Quantifying Relationships in Ethnic Subgroups. Diabetes Care. 2017;40(3):338-45.
24. Olier I, Springate DA, Ashcroft DM, Doran T, Reeves D, Planner C, et al. Modelling Conditions and Health Care Processes in Electronic Health Records: An Application to Severe Mental Illness with the Clinical Practice Research Datalink. Plos One. 2016;11(2).
25. Springate DA, Kontopantelis E, Ashcroft DM, Olier I, Parisi R, Chamapiwa E, et al. ClinicalCodes: An Online Clinical Codes Repository to Improve the Validity and Reproducibility of Research Using Electronic Medical Records. PLoS ONE. 2014;9(6):e99825.
26. Staniszewska S, Brett J, Mockford C, Barber R. The GRIPP checklist: Strengthening the quality of patient and public involvement reporting in research. International Journal of Technology Assessment in Health Care. 2011;27(4):391-9.
27. Lipscombe LL, Hux JE. Trends in diabetes prevalence, incidence, and mortality in Ontario, Canada 1995–2005: a population-based study. Lancet. 2007;369:750-56.
28. Onat A, Hergenc G, Uyarel H, Can G, Ozhan H. Prevalence, incidence, predictors and outcome of type 2 diabetes in Turkey. Anadolu kardiyoloji dergisi : AKD = the Anatolian journal of cardiology. 2006;6(4):314-21.
29. International Diabetes Federation (IDF). IDF Diabetes Atlas - 7th Edition. 2015 [cited 2017 08 February]; Available from: http://www.diabetesatlas.org/.
30. Lin CC, Li CI, Hsiao CY, Liu CS, Yang SY, Lee CC, et al. Time trend analysis of the prevalence and incidence of diagnosed type 2 diabetes among adults in Taiwan from 2000 to 2007: a population-based study. BMC Public Health. 2013;13(318).
31. Gonzalez ELM, Johansson S, Wallander MA, Rodriguez LAG. Trends in the prevalence and incidence of diabetes in the UK: 1996-2005. J Epidemiol Community Health. 2009;63(4):332-6.
32. Evans JMM, Barnett KN, Ogston SA, Morris AD. Increasing prevalence of type 2 diabetes in a Scottish population: effect of increasing incidence or decreasing mortality? Diabetologia. 2007;50(4):729-32.
33. Young BA, Lin E, Von Korff M, Simon G, Ciechanowski P, Ludman EJ, et al. Diabetes complications severity index and risk of mortality, hospitalization, and healthcare utilization. Am J Manag Care. 2008;14(1):15-24.
34. Runkel M, Muller S, Brydniak R, Runkel N. Downgrading of type 2 diabetes mellitus (T2DM) after obesity surgery: duration and severity matter. Obesity surgery. 2015;25(3):494-9. Epub 2014/09/12.
35. Okano K, Araki M, Yamamoto M, Ishikawa T, Ichihara K, Yamada O. Exploration of hematological and immunological changes associated with the severity of type 2 diabetes mellitus in Japan. Nursing & health sciences. 2008;10(1):65-9. Epub 2008/02/09.
36. Fram EB, Moazami S, Stern JM. The Effect of Disease Severity on 24-Hour Urine Parameters in Kidney Stone Patients With Type 2 Diabetes. Urology. 2016;87:52-9. Epub 2015/11/04.

For peer review only - http://bmjopen.bmj.com/site/about/guidelines.xhtml

37. Eldor R, Klieger Y, Sade-Feldman M, Vaknin I, Varfolomeev I, Fuchs C, et al. CD247, a novel T cell-derived diagnostic and prognostic biomarker for detecting disease progression and severity in patients with type 2 diabetes. Diabetes care. 2015;38(1):113-8. Epub 2014/11/05.
38. Loprinzi PD, Loenneke JP. Evidence of a Link Between Grip Strength and Type 2 Diabetes Prevalence and Severity Among a National Sample of U.S. Adults. Journal of physical activity & health. 2016;13(5):558-61. Epub 2015/11/10.
39. Nahin RL, Byrd-Clark D, Stussman BJ, Kalyanaraman N. Disease severity is associated with the use of complementary medicine to treat or manage type-2 diabetes: data from the 2002 and 2007 National Health Interview Survey. BMC complementary and alternative medicine. 2012;12:193. Epub 2012/10/24.
40. Linzer M, Pierce C, Lincoln E, Miller DR, Payne SM, Clark JA, et al. Preliminary validation of a patient-based self-assessment measure of severity of illness in type 2 diabetes: results from the pilot phase of the Veterans Health Study. The Journal of ambulatory care management. 2005;28(2):167-76. Epub 2005/06/01.
41. Gatlin PK, Insel KC. Severity of Type 2 Diabetes, Cognitive Function, and Self-Care. Biological research for nursing. 2015;17(5):540-8. Epub 2014/11/14.
42. Windrum P, Garcia-Goni M, Coad H. The Impact of Patient-Centered versus Didactic Education Programs in Chronic Patients by Severity: The Case of Type 2 Diabetes Mellitus. Value in health : the journal of the International Society for Pharmacoeconomics and Outcomes Research. 2016;19(4):353-62. Epub 2016/06/23.
43. Svensson E, Berencsi K, Sander S, Mor A, Rungby J, Nielsen JS, et al. Association of parental history of type 2 diabetes with age, lifestyle, anthropometric factors, and clinical severity at type 2 diabetes diagnosis: results from the DD2 study. Diabetes/metabolism research and reviews. 2016;32(3):308-15. Epub 2015/09/27.
44. Powell DS, Maksoud H, Charge SB, Moffitt JH, Desai M, Da Silva Fihlo RL, et al. Apolipoprotein E genotype, islet amyloid deposition and severity of Type 2 diabetes. Diabetes research and clinical practice. 2003;60(2):105-10. Epub 2003/04/23.
45. Jiang Q, Lyu XM, Yuan Y, Wang L. Plasma miR-21 expression: an indicator for the severity of Type 2 diabetes with diabetic retinopathy. Bioscience reports. 2017;37(2). Epub 2017/01/22.
46. Campbell-Tofte J, Hansen HS, Mu H, Molgaard P. Increased lipids in non-lipogenic tissues are indicators of the severity of type 2 diabetes in mice. Prostaglandins, leukotrienes, and essential fatty acids. 2007;76(1):9-18. Epub 2006/11/14.
47. Garcia-Elorriaga G, Padilla-Reyes M, Cruz-Olivo F, Fuentes-Allen JL, del Rey-Pineda G, Gonzalez-Bonilla C. [Pro-inflammatory cytokines related to severity and mortality in type 2 diabetes patients with soft tissue infection]. Revista medica del Instituto Mexicano del Seguro Social. 2012;50(3):237-41. Epub 2012/11/28. Citocinas, diabetes e infeccion de tejidos blandos. Su relacion con la severidad y mortalidad.
48. Kontopantelis E, Buchan I, Reeves D, Checkland K, Doran T. Relationship between quality of care and choice of clinical computing system: retrospective analysis of family practice performance under the UK's quality and outcomes framework. BMJ open. 2013;3:e003190.
49. Dunning T, Martin M. Type 2 diabetes: is it serious? Journal of Diabetes Nursing. 1998;2(3):70-6.

For peer review only - http://bmjopen.bmj.com/site/about/guidelines.xhtml

Table 1 The main (sub) domains identified to quantify the severity of type 2 diabetes

	Severity domain	Severity sub-domain
**1.**	**Risk factors***	• Duration of type 2 diabetes[39]<br>• Body mass index (BMI)<br>• Hypertension<br>• Hyperlipidaemia<br>• Personal/Family history of cardiovascular disease<br>• Blood glucose levels<br>○ Glycated haemoglobin (HbA_{1c})[34]<br>○ Fasting (FBG) and random (RBG) blood glucose
**2.**	**Type/pattern of antidiabetic treatment, insulin use and other therapies**	• Antidiabetic therapy ever;[39] Therapies with cardiovascular benefits versus other; Changes in drug treatments;[49] or the number of prescribed treatments[41]<br>• Insulin use: prescription ever or within 1 year of diagnosis; Insulin initiation:[15] time to initiation<br>• Other therapies: angiotensin converting enzyme inhibitors (ACEI) and lipid-regulating therapies
**3.**	**Microvascular diabetic complications**[15,33]	• Neuropathy (Foot ulcer, Charcot foot, gangrene, amputation)<br>• Nephropathy<br>• Retinopathy (laser therapy and blindness)
**4.**	**Renal disease**	• Microalbuminuria and proteinuria<br>• Moderate-severe chronic kidney disease (CKD) stages 3 and 4[33]<br>• End-stage renal disease (ESRD): kidney transplant and dialysis
**5.**	**Cardiovascular and Cerebrovascular disease**	• Atherosclerosis[15,33]<br>• Myocardial infarction (MI) [15,33]<br>• Angina[15,33]<br>• Atrial/ventricular fibrillation (AF)/(VF)[33]<br>• Heart valve disease<br>• Heart failure (HF)[33]<br>• Peripheral vascular disease (PVD)[33]<br>• Transient ischemic attack (TIA)<br>• Ischaemic stroke, haemorrhagic stroke [15,33]
**6.**	**Cardiovascular and Cerebrovascular interventions**	• Coronary artery bypass graft (CABG)<br>• Coronary artery interventions (PCI/PTCA)<br>• Endovascular aneurysm repair (EVAR)<br>• PVD stenting and bypass procedures<br>• Heart valve interventions<br>• Use of defibrillator<br>• Carotid artery events, stenting and bypass interventions
**7.**	**Other comorbidities**	• Anxiety<br>• Depression<br>• Dementia<br>• Cognitive impairment
**8.**	**Hospital admissions**	• Any-cause hospital admissions<br>• Diabetes-attributable admission<br>• Cardiovascular disease-related admission
**9.**	**Emergency diabetes-related event**	• Hypoglycaemia<br>• Hyperosmolar hyperglycaemic state (HHS)[33]<br>• Diabetic ketoacidosis (DKA) or other coma[33]

**Other demographic data (such as age, gender and the level of deprivation) are important predictors for adverse outcomes and will be included in the later risk-prediction analysis.*

For peer review only - http://bmjopen.bmj.com/site/about/guidelines.xhtml

Figure 1 Severity hierarchy of microvascular diabetic complications

Figure 2 Severity hierarchy of cerebrovascular domains

Figure 3 Severity hierarchy of coronary and vascular domains

Figure 4 Severity hierarchy of cardiovascular disease domains (heart rate, rhythm and conduction)

For peer review only

For peer review only - http://bmjopen.bmj.com/site/about/guidelines.xhtml

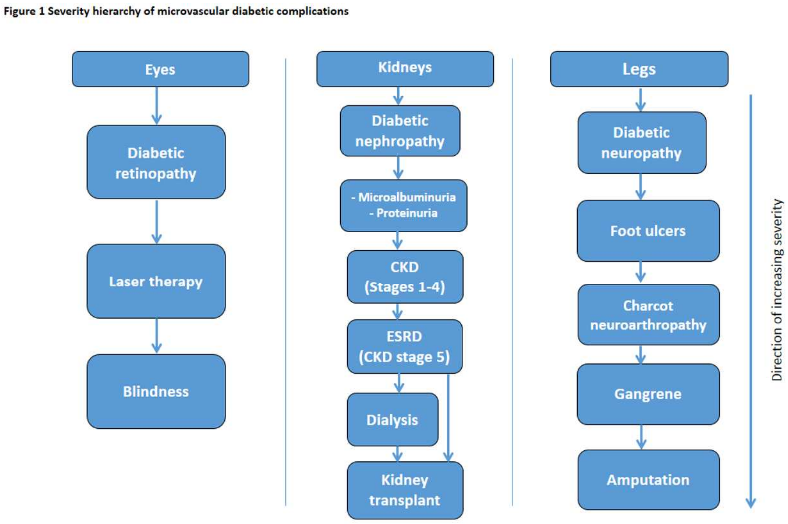

Figure 1 Severity hierarchy of microvascular diabetic complications

338x190mm (300 x 300 DPI)

Figure 2 Severity hierarchy of cerebrovascular domains

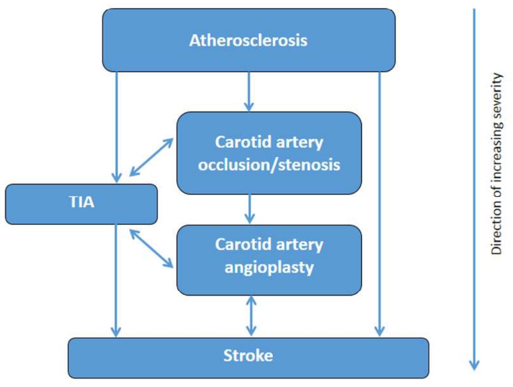

Figure 2 Severity hierarchy of cerebrovascular domains

338x190mm (300 x 300 DPI)

review only

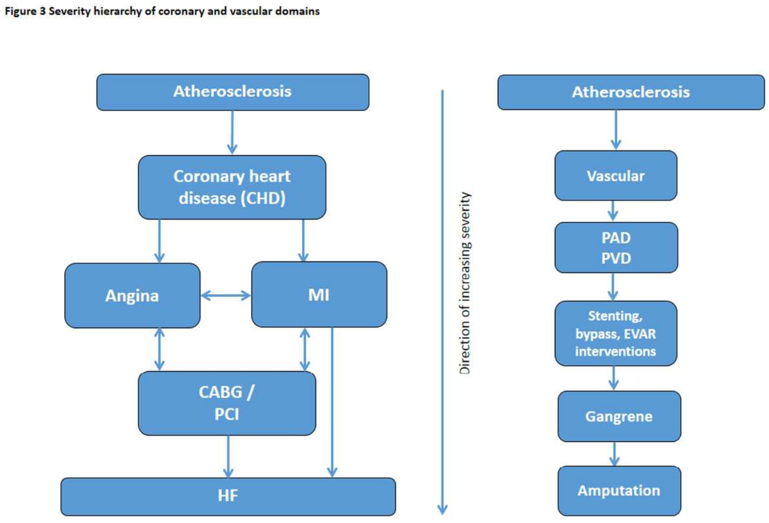

Figure 3 Severity hierarchy of coronary and vascular domains

338x190mm (300 x 300 DPI)

Figure 4 Severity hierarchy of cardiovascular disease domains (heart rate, rhythm and conduction)

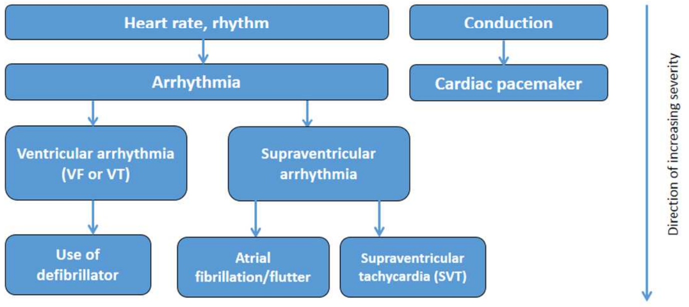

Figure 4 Severity hierarchy of cardiovascular disease domains (heart rate, rhythm and conduction)

338x190mm (300 x 300 DPI)

Supplementary data

Table S1 Inclusion medical codes for type 2 diabetes

	Medical code	Read term
**1.**	506	Non-insulin dependent diabetes mellitus
**2.**	758	Type 2 diabetes mellitus
**3.**	1407	Insulin treated Type 2 diabetes mellitus
**4.**	4513	Non-insulin dependent diabetes mellitus
**5.**	5884	NIDDM - Non-insulin dependent diabetes mellitus
**6.**	8403	Non-insulin dependent diabetes mellitus - poor control
**7.**	12640	Type 2 diabetes mellitus with nephropathy
**8.**	12736	Type 2 diabetes mellitus with gangrene
**9.**	17262	Non-insulin-dependent diabetes mellitus with retinopathy
**10.**	17859	Type 2 diabetes mellitus
**11.**	18143	Type II diabetes mellitus with arthropathy
**12.**	18209	Type 2 diabetes mellitus with renal complications
**13.**	18219	Type II diabetes mellitus
**14.**	18264	Insulin treated Type II diabetes mellitus
**15.**	18278	Insulin treated Type 2 diabetes mellitus
**16.**	18390	Type 2 diabetes mellitus with persistent microalbuminuria
**17.**	18425	Type 2 diabetes mellitus with polyneuropathy
**18.**	18496	Type 2 diabetes mellitus with retinopathy
**19.**	18777	Type 2 diabetes mellitus with renal complications
**20.**	22884	Type II diabetes mellitus
**21.**	24458	Type II diabetes mellitus - poor control
**22.**	24693	Non-insulin dependent diabetes mellitus with arthropathy
**23.**	24836	Type 2 diabetes mellitus with nephropathy
**24.**	25041	Dietary advice for type II diabetes
**25.**	25591	Type 2 diabetes mellitus with exudative maculopathy
**26.**	25627	Type 2 diabetes mellitus - poor control
**27.**	26054	Type 2 diabetes mellitus with persistent proteinuria
**28.**	29979	Non-insulin-dependent diabetes mellitus without complication
**29.**	32627	Type 2 diabetes mellitus with ketoacidosis
**30.**	34268	Type 2 diabetes mellitus with neurological complications
**31.**	34450	Hyperosmolar non-ketotic state in type 2 diabetes mellitus
**32.**	34912	Non-insulin dependent diabetes mellitus with ulcer
**33.**	35385	Type 2 diabetes mellitus with neuropathic arthropathy
**34.**	36633	Hyperosmolar non-ketotic state in type 2 diabetes mellitus
**35.**	37648	Insulin treated non-insulin dependent diabetes mellitus
**36.**	37806	Type 2 diabetes mellitus with peripheral angiopathy
**37.**	40401	Non-insulin dependent diabetes mellitus with gangrene
**38.**	42762	Type 2 diabetes mellitus with retinopathy
**39.**	43227	Type II diabetes mellitus with multiple complications
**40.**	43785	Non-insulin dependent diabetes mellitus with hypoglyca coma
**41.**	44779	Type 2 diabetes mellitus with diabetic cataract
**42.**	44982	Type 2 diabetes mellitus with diabetic cataract
**43.**	45467	Non-insulin dependent diabetes mellitus with polyneuropathy
**44.**	45913	Type 2 diabetes mellitus - poor control
**45.**	45919	Type 2 diabetes mellitus with neurological complications

For peer review only - http://bmjopen.bmj.com/site/about/guidelines.xhtml

**46.**	46150	Type 2 diabetes mellitus with gangrene
**47.**	46917	Type 2 diabetes mellitus with hypoglycaemic coma
**48.**	47315	Type II diabetes mellitus - poor control
**49.**	47321	Type 2 diabetes mellitus with ophthalmic complications
**50.**	47409	Type II diabetes mellitus with polyneuropathy
**51.**	47816	Type II diabetes mellitus with neuropathic arthropathy
**52.**	47954	Type 2 diabetes mellitus without complication
**53.**	48192	Type II diabetes mellitus with diabetic cataract
**54.**	49074	Type 2 diabetes mellitus with ulcer
**55.**	49655	Type II diabetes mellitus with retinopathy
**56.**	49869	Type 2 diabetes mellitus with arthropathy
**57.**	50225	Type II diabetes mellitus with renal complications
**58.**	50429	Non-insulin-dependent diabetes mellitus with ophthalm comps
**59.**	50527	Type II diabetes mellitus with polyneuropathy
**60.**	50609	Pre-existing diabetes mellitus, non-insulin-dependent
**61.**	50813	Type II diabetes mellitus with mononeuropathy
**62.**	51756	Type 2 diabetes mellitus with ketoacidotic coma
**63.**	52303	Non-insulin-dependent diabetes mellitus with renal comps
**64.**	53392	Type II diabetes mellitus without complication
**65.**	54899	Type II diabetes mellitus with peripheral angiopathy
**66.**	55075	Type II diabetes mellitus with ulcer
**67.**	55842	Non-insulin-dependent diabetes mellitus with neuro comps
**68.**	56268	Type II diabetes mellitus with hypoglycaemic coma
**69.**	57278	Type II diabetes mellitus with renal complications
**70.**	58604	Type II diabetes mellitus with retinopathy
**71.**	59253	Type 2 diabetes mellitus with arthropathy
**72.**	59365	Non-insulin dependent diabetes mellitus with nephropathy
**73.**	59725	Type II diabetes mellitus with ophthalmic complications
**74.**	60699	Type 2 diabetes mellitus with peripheral angiopathy
**75.**	60796	Type II diabetes mellitus with persistent proteinuria
**76.**	61071	Type 2 diabetes mellitus with hypoglycaemic coma
**77.**	62107	Type II diabetes mellitus with gangrene
**78.**	62146	Non-insulin-dependent diabetes mellitus with multiple comps
**79.**	62674	Type 2 diabetes mellitus with mononeuropathy
**80.**	63690	Type 2 diabetes mellitus with gastroparesis
**81.**	64571	Type II diabetes mellitus with nephropathy
**82.**	64668	Insulin treated Type II diabetes mellitus
**83.**	65267	Type 2 diabetes mellitus with multiple complications
**84.**	65704	Type 2 diabetes mellitus with ulcer
**85.**	66965	Type 2 diabetes mellitus with neuropathic arthropathy
**86.**	67905	Type II diabetes mellitus with neurological complications
**87.**	69278	Non-insulin depend diabetes mellitus with diabetic cataract
**88.**	70316	Type 2 diabetes mellitus with ophthalmic complications
**89.**	72320	Non-insulin dependent diabetes mellitus with mononeuropathy
**90.**	83532	Diabetes type 2 review
**91.**	85991	Type II diabetes mellitus with persistent microalbuminuria
**92.**	91646	Type II diabetes mellitus with ulcer
**93.**	93727	Type II diabetes mellitus with diabetic cataract
**94.**	95351	Type II diabetes mellitus with mononeuropathy
**95.**	98616	Type II diabetes mellitus with neurological complications
**96.**	98723	Type II diabetes mellitus with hypoglycaemic coma
**97.**	100964	Type II diabetes mellitus with ophthalmic complications
**98.**	101801	Type II diabetic dietary review
**99.**	102201	Type II diabetes mellitus with nephropathy

For peer review only - http://bmjopen.bmj.com/site/about/guidelines.xhtml

**100.**	102611	Type 2 diabetic dietary review
**101.**	103902	Type II diabetes mellitus with arthropathy
**102.**	104323	Type II diabetes mellitus with gangrene
**103.**	104639	Type II diabetes mellitus with peripheral angiopathy
**104.**	105784	Type 2 diabetes mellitus without complication
**105.**	106061	Type II diabetes mellitus with ketoacidotic coma
**106.**	106528	Type II diabetes mellitus with ketoacidosis
**107.**	107701	Hyperosmolar non-ketotic state in type II diabetes mellitus
**108.**	107824	Type II diabetes mellitus in remission
**109.**	108005	Type 2 diabetes mellitus with multiple complications
**110.**	109103	Type II diabetes mellitus without complication
**111.**	109197	Type II diabetes mellitus with neuropathic arthropathy
**112.**	109865	Type 2 diabetes mellitus with polyneuropathy

For peer review only

For peer review only - http://bmjopen.bmj.com/site/about/guidelines.xhtml

Table S2 Exclusion type 1 diabetes codes

	Medical code	Read term
**1.**	1038	Insulin dependent diabetes mellitus
**2.**	1549	Type 1 diabetes mellitus
**3.**	1647	Insulin dependent diabetes mellitus
**4.**	6509	Insulin dependent diabetes mellitus with retinopathy
**5.**	6791	Insulin dependent diabetes mellitus - poor control
**6.**	10418	Type 1 diabetes mellitus with nephropathy
**7.**	10692	Type 1 diabetes mellitus with ketoacidosis
**8.**	12455	Type I diabetes mellitus
**9.**	17545	Type I diabetes mellitus with diabetic cataract
**10.**	17858	Type 1 diabetes mellitus
**11.**	18230	Type 1 diabetes mellitus with neuropathic arthropathy
**12.**	18387	Type 1 diabetes mellitus with retinopathy
**13.**	18505	IDDM-Insulin dependent diabetes mellitus
**14.**	18642	Type 1 diabetes mellitus with arthropathy
**15.**	18683	Type 1 diabetes mellitus with ulcer
**16.**	21983	Type 1 diabetes mellitus with renal complications
**17.**	22871	Type 1 diabetes mellitus with exudative maculopathy
**18.**	24423	Type I diabetes mellitus
**19.**	24490	Diabetes mellitus, juvenile type, no mention of complication
**20.**	24694	Insulin dependent diabetes mellitus with mononeuropathy
**21.**	26855	Unstable insulin dependent diabetes mellitus
**22.**	30294	Type 1 diabetes mellitus with persistent microalbuminuria
**23.**	30323	Type 1 diabetes mellitus with persistent proteinuria
**24.**	31310	Insulin dependent diabetes maturity onset
**25.**	32359	Perceived control of insulin-dependent diabetes
**26.**	35288	Type 1 diabetes mellitus - poor control
**27.**	38161	Type I diabetes mellitus with retinopathy
**28.**	39070	Type 1 diabetes mellitus with hypoglycaemic coma
**29.**	39809	Insulin dependent diab mell with neuropathic arthropathy
**30.**	40023	Diabetes mellitus, juvenile type, with hyperosmolar coma
**31.**	40682	Type 1 diabetes mellitus maturity onset
**32.**	40837	Type 1 diabetes mellitus with ketoacidotic coma
**33.**	41049	Type 1 diabetes mellitus with retinopathy
**34.**	41716	Insulin dependent diabetes mellitus with polyneuropathy
**35.**	42567	Diabetes mellitus, juvenile type, with ketoacidotic coma
**36.**	42729	Type I diabetes mellitus with hypoglycaemic coma
**37.**	42831	Type 1 diabetes mellitus with neurological complications
**38.**	43921	Unstable type 1 diabetes mellitus
**39.**	44260	Insulin dependent diabetes mellitus with diabetic cataract
**40.**	44440	Insulin dependent diabetes mellitus with hypoglycaemic coma
**41.**	44443	Insulin dependent diabetes mellitus with ulcer
**42.**	45276	Insulin dependent diabetes mellitus with multiple complicat
**43.**	45914	Type 1 diabetes mellitus - poor control
**44.**	46301	Type 1 diabetes mellitus with polyneuropathy
**45.**	46850	Type I diabetes mellitus - poor control
**46.**	46963	Insulin-dependent diabetes mellitus with renal complications

For peer review only - http://bmjopen.bmj.com/site/about/guidelines.xhtml

**47.**	47582	Type 1 diabetes mellitus with renal complications
**48.**	47649	Type 1 diabetes mellitus with ophthalmic complications
**49.**	47650	Type 1 diabetes mellitus with multiple complications
**50.**	49146	Type I diabetes mellitus with neurological complications
**51.**	49276	Insulin-dependent diabetes mellitus with ophthalmic comps
**52.**	49554	Type 1 diabetes mellitus with diabetic cataract
**53.**	49949	Unstable type I diabetes mellitus
**54.**	50960	Pre-existing diabetes mellitus, insulin-dependent
**55.**	51261	Insulin dependent diabetes mellitus
**56.**	51957	Type I diabetes mellitus with ulcer
**57.**	52104	Insulin dependent diabetes mellitus with multiple complicatn
**58.**	52283	Insulin-dependent diabetes mellitus with neurological comps
**59.**	53200	Diabetes mellitus, juvenile type, with ketoacidosis
**60.**	54008	Type 1 diabetes mellitus with neuropathic arthropathy
**61.**	54600	Unstable insulin dependent diabetes mellitus
**62.**	55239	Type 1 diabetes mellitus with gastroparesis
**63.**	56448	Insulin-dependent diabetes without complication
**64.**	57621	Insulin dependent diabetes mellitus with nephropathy
**65.**	60107	Unstable type I diabetes mellitus
**66.**	60208	Type I diabetes mellitus with neuropathic arthropathy
**67.**	60499	Insulin dependent diabetes mellitus with gangrene
**68.**	61344	Type I diabetes mellitus with renal complications
**69.**	61829	Type 1 diabetes mellitus with neurological complications
**70.**	62209	Type I diabetes mellitus with ketoacidosis
**71.**	62352	Type I diabetes mellitus with arthropathy
**72.**	62613	Type I diabetes mellitus without complication
**73.**	63017	Type I diabetes mellitus maturity onset
**74.**	64446	Insulin dependent diab mell with peripheral angiopathy
**75.**	65616	Insulin dependent diabetes mellitus with arthropathy
**76.**	66145	Type I diabetes mellitus with ketoacidotic coma
**77.**	66872	Type I diabetes mellitus with nephropathy
**78.**	68105	Type 1 diabetes mellitus with mononeuropathy
**79.**	68390	Type 1 diabetes mellitus with ulcer
**80.**	68792	Diabetes mellitus, juvenile type, + unspecified complication
**81.**	69043	Dietary advice for type I diabetes
**82.**	69676	Type 1 diabetes mellitus without complication
**83.**	69748	Diabetes mellitus, juvenile type, + ophthalmic manifestation
**84.**	69993	Type 1 diabetes mellitus with gangrene
**85.**	70766	Type 1 diabetes mellitus with hypoglycaemic coma
**86.**	72702	Insulin dependent diabetes mellitus - poor control
**87.**	85660	Diabetes type 1 review
**88.**	91942	Type I diabetes mellitus with multiple complications
**89.**	91943	Type I diabetes mellitus with polyneuropathy
**90.**	93468	Type 1 diabetes mellitus with peripheral angiopathy
**91.**	93875	Insulin dependent diabetes mellitus with retinopathy
**92.**	93878	Type I diabetes mellitus with ulcer
**93.**	93922	Diabetes mellitus, juvenile type, with renal manifestation
**94.**	95343	Type I diabetes mellitus with retinopathy
**95.**	95992	Type I diabetes mellitus without complication
**96.**	96235	Type I diabetes mellitus maturity onset

For peer review only - http://bmjopen.bmj.com/site/about/guidelines.xhtml

**97.**	97446	Type 1 diabetes mellitus maturity onset
**98.**	97474	Unstable type 1 diabetes mellitus
**99.**	97849	Insulin dependent diabetes maturity onset
**100.**	97894	Type I diabetes mellitus with exudative maculopathy
**101.**	98071	Insulin-dependent diabetes mellitus with ophthalmic comps
**102.**	98392	Maturity onset diabetes in youth type 1
**103.**	98704	Insulin dependent diabetes mellitus with ulcer
**104.**	99231	Type I diabetes mellitus with mononeuropathy
**105.**	99311	Type I diabetes mellitus with ophthalmic complications
**106.**	99716	Insulin dependent diabetes mellitus with hypoglycaemic coma
**107.**	99719	Insulin-dependent diabetes without complication
**108.**	100770	Insulin dependent diabetes mellitus with diabetic cataract
**109.**	101311	Insulin dependent diabetes mellitus with polyneuropathy
**110.**	101735	Insulin-dependent diabetes mellitus with neurological comps
**111.**	102112	Type I diabetes mellitus with gangrene
**112.**	102163	Insulin dependent diabetes mellitus with nephropathy
**113.**	102620	Type I diabetes mellitus with persistent microalbuminuria
**114.**	102704	Type I diabetic dietary review
**115.**	102740	Type 1 diabetes mellitus with ophthalmic complications
**116.**	102946	Insulin-dependent diabetes mellitus with renal complications
**117.**	104453	Type 1 diabetic dietary review
**118.**	105337	Type I diabetes mellitus - poor control
**119.**	108007	Type I diabetes mellitus with multiple complications
**120.**	108360	Type I diabetes mellitus in remission
**121.**	108724	Type I diabetes mellitus with gastroparesis
**122.**	109051	Insulin dependent diabetes mellitus with gangrene
**123.**	109628	Type 1 diabetes mellitus in remission
**124.**	109837	Type I diabetes mellitus with renal complications
**125.**	109878	Diet advice for insulin-dependent diabetes
**126.**	110400	Type 1 diabetes mellitus with diabetic cataract
**127.**	111106	Type 1 diabetes mellitus without complication

For peer review only

Supplementary material to paper "**Using electronic health records to quantify and stratify the severity of type 2 diabetes in primary care in England: rationale and cohort study design" Zghebi et al. 2017**

Figure S1 Severity hierarchy of cardiovascular disease domains (cardiac muscle- myocardium and pericardium).

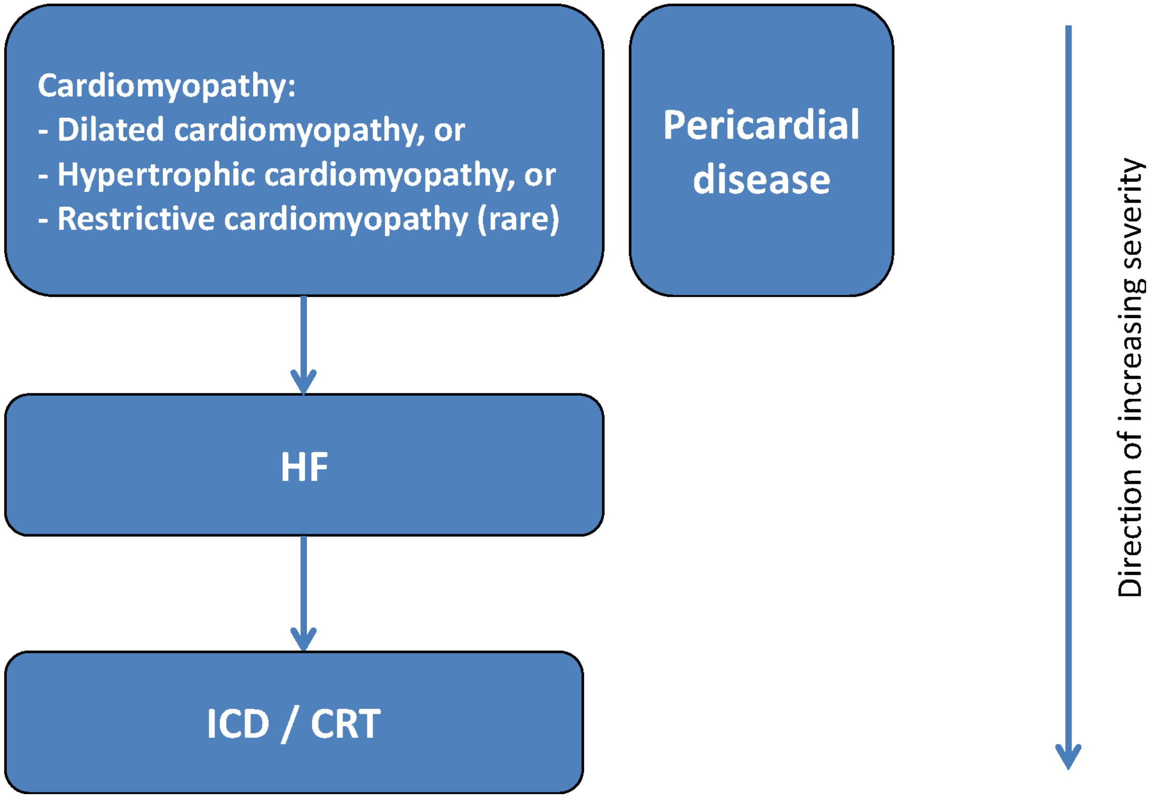

For peer review only - http://bmjopen.bmj.com/site/about/guidelines.xhtml

Supplementary material to paper "**Using electronic health records to quantify and stratify the severity of type 2 diabetes in primary care in England: rationale and cohort study design" Zghebi et al. 2017**

Figure S2 Severity hierarchy of cardiovascular disease domains (Heart valve diseases)

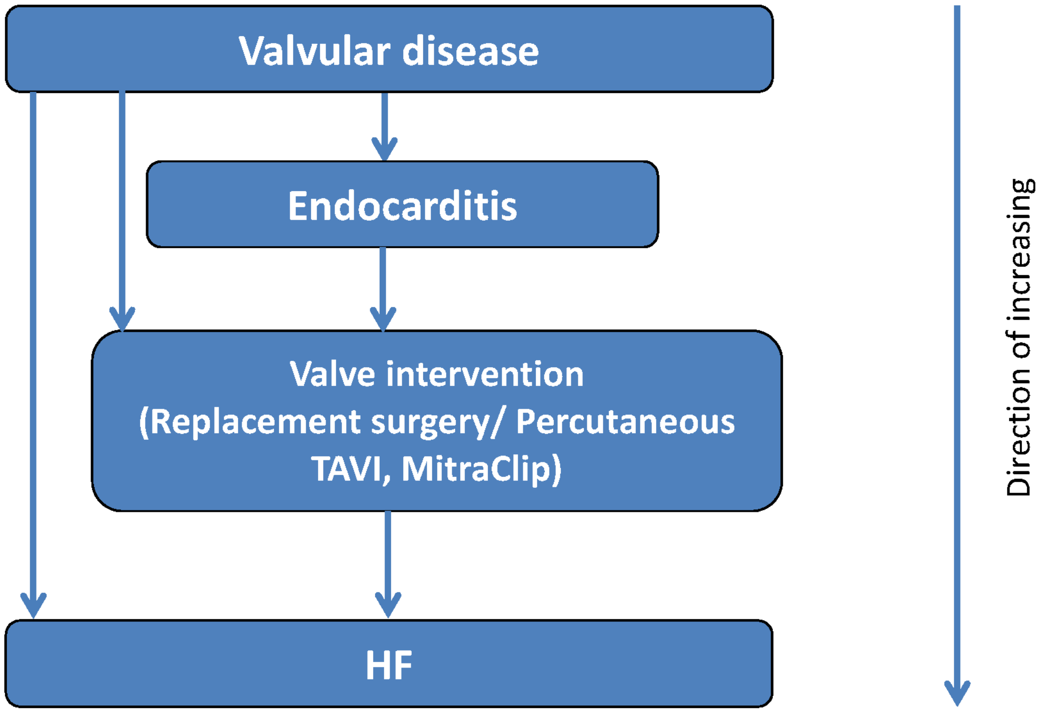

Supplementary material to paper "**Using electronic health records to quantify and stratify the severity of type 2 diabetes in primary care in England: rationale and cohort study design" Zghebi et al. 2017**

Table S3 Factors used in previous studies to define severity of diabetes

Study ID	Factors used to define diabetes severity / Outcome(s)	Severity scale/ levels	Validation study
**Gini et al. (2016)**[(1)]	• Indication for insulin therapy<br>• The presence of diabetes-related complications<br><br>**Outcomes:**<br>Assessing the positive predictive value (PPV) for the presence/absence of type 2 diabetes against the GP's response to the questionnaire and measure the agreement on the severity level.	Four levels of T2DM severity:<br>• **Level 1**: Clinical definition of the disease, no indication for Insulin therapy and no complications listed in Level 3.<br>• **Level 2**: as in Level 1 except insulin is indicated.<br>• **Level 3**: as in Level 1 except with one of the following complications: (1) arterial stenosis, angina, MI, TIA, ischaemic stroke, intermittent claudication, diabetic foot ulcer, lower limb amputation, (2) retinopathy, (3) incipient or overt diabetic nephropathy/ dialysis.<br>• **Level 4**: As in Level 3 except insulin is indicated	A random sample of identified cases was validated by interviewing their self-selected general practitioners (GPs).
**Grootenhuis et al. (1994)**[(2)]	Type 2 diabetes symptom severity items categorised over six clinical dimensions:<br>• Hyperglycaemic<br>• Hypoglycaemic<br>• Psychological<br>• Cardiovascular<br>• Neuropathic<br>• Ophthalmological<br><br>**Outcomes:**<br>To measure differences in symptom severity between patients and detect changes over time within patients.	A 34-item patient-derived checklist used as a measure for type 2 diabetes symptom severity.	The face and content validity of severity items and dimension structures were based on literature and experiences of diabetologists.
**Young et al. (2008)**[(3)]	From laboratory data and diabetes-related complications:<br>• Retinopathy<br>• Nephropathy<br>• Neuropathy<br>• Cerebrovascular<br>• Cardiovascular<br>• Peripheral vascular disease<br>• Metabolic<br><br>**Outcomes:**<br>Risk of mortality, hospitalisation and healthcare utilisation.	Developed the Diabetes Complication Severity Index (DCSI) that is categorised to two or three levels (0=no abnormality; 1=some abnormality, 2=severe abnormality) depending on the severity of the complication. So if no abnormalities were present, the patient received no score, a 1 was added to the DCSI if patient had any complication classified as some abnormality and 2 was added if patient had any complication classified as severe abnormality.	The study design was a validation sample

For peer review only - http://bmjopen.bmj.com/site/about/guidelines.xhtml

Supplementary material to paper "**Using electronic health records to quantify and stratify the severity of type 2 diabetes in primary care in England: rationale and cohort study design" Zghebi et al. 2017**

References

1. Gini R, Schuemie MJ, Mazzaglia G, Lapi F, Francesconi P, Pasqua A, et al. Automatic identification of type 2 diabetes, hypertension, ischaemic heart disease, heart failure and their levels of severity from Italian General Practitioners' electronic medical records: a validation study. BMJ open. 2016;6(12):e012413. Epub 2016/12/13.
2. Grootenhuis PA, Snoek FJ, Heine RJ, Bouter LM. Development of a type 2 diabetes symptom checklist: a measure of symptom severity. Diabetic medicine : a journal of the British Diabetic Association. 1994;11(3):253-61. Epub 1994/04/01.
3. Young BA, Lin E, Von Korff M, Simon G, Ciechanowski P, Ludman EJ, et al. Diabetes complications severity index and risk of mortality, hospitalization, and healthcare utilization. Am J Manag Care. 2008;14(1):15-24.

For peer review only

For peer review only - http://bmjopen.bmj.com/site/about/guidelines.xhtml

BMJ Open

Using electronic health records to quantify and stratify the severity of type 2 diabetes in primary care in England: rationale and cohort study design

Journal:	*BMJ Open*
Manuscript ID	bmjopen-2017-020926.R2
Article Type:	Protocol
Date Submitted by the Author:	06-Mar-2018
Complete List of Authors:	Zghebi, Salwa; University of Manchester, NIHR School for Primary Care Research, Centre for Primary Care<br>Rutter, Martin; University of Manchester Faculty of Biology, Medicine and Health, Division of Diabetes, Endocrinology and Gastroenterology; Manchester University NHS Foundation Trust, Manchester Diabetes Centre<br>Ashcroft, Darren; University of Manchester, Centre for Pharmacoepidemiology and Drug Safety,<br>Salisbury, Chris; University of Bristol, Academic Unit of Primary Health Care<br>Mallen, Christian; Keele University, Arthritis Research UK Primary Care Centre<br>Chew-Graham, Carolyn; University of Keele<br>Reeves, David; University of Manchester, Institute of Population Health; Centre for Biostatistics<br>van Marwijk, Harm; University of Brighton<br>Qureshi, Nadeem; University of Nottingham, School of Medicine<br>Weng, Stephen; University of Nottingham, NIHR School of Primary Care Research<br>Peek, N; Health e-Research Centre, Institute of Population Health, University of Manchester<br>Planner, Claire; NIHR School for Primary Care Research, Centre for Primary Care, Institute of Population Health<br>Nowakowska, Magdalena ; University of Manchester, NIHR School for Primary Care Research, Centre for Primary Care<br>Mamas, Mamas; Keele Cardiovascular Research group, Centre for Prognosis Research<br>Kontopantelis, Evangelos; University of Manchester, Faculty of Biology, Medicine and Health; NIHR School for Primary Care Research, Centre for Primary Care
**Primary Subject Heading**:	Diabetes and endocrinology
Secondary Subject Heading:	General practice / Family practice, Health services research
Keywords:	Type 2 diabetes, Electronic Health Records, PRIMARY CARE, Hospitalisation records, Cardiovascular disease, Diabetes Severity Algorithm

For peer review only

Using electronic health records to quantify and stratify the severity of type 2 diabetes in primary care in England: rationale and cohort study design

Salwa S Zghebi,[1,2] Martin K Rutter,[3,4] Darren M Ashcroft,[5] Chris Salisbury,[6] Christian Mallen,[7] Carolyn A. Chew-Graham,[7] David Reeves,[1] Harm Van marwijk,[8] Nadeem Qureshi,[9] Stephen Weng,[9] Niels Peek,[10] Claire Planner,[1] Magdalena Nowakowska,[1,2] Mamas Mamas,[11] Evangelos Kontopantelis[1,2]

1. Division of Population Health, Health Services Research and Primary Care, School of Health Sciences, Faculty of Biology, Medicine and Health, Health Innovation Manchester, University of Manchester, Manchester, UK.

2. NIHR School for Primary Care Research, University of Manchester, Manchester, UK.

3. Division of Diabetes, Endocrinology and Gastroenterology, School of Medical Sciences, Faculty of Biology, Medicine and Health, University of Manchester, Manchester, M13 9PL, UK.

4. Manchester Diabetes Centre, 193 Hathersage Rd, Manchester University NHS Foundation Trust, Manchester Academic Health Science Centre, Manchester, M13 0JE, UK.

5. Division of Pharmacy and Optometry, School of Health Sciences, Faculty of Biology, Medicine and Health, Health Innovation Manchester, University of Manchester, Manchester, UK.

6. Centre for Academic Primary Care, Population Health Sciences, Bristol Medical School, University of Bristol, Bristol, UK.

7. Research Institute for Primary Care and Health Sciences, Keele University, Staffordshire, UK.

8. Division of Primary Care and Public Health, Brighton and Sussex Medical School, University of Brighton, Brighton, UK.

9. School of Medicine, University of Nottingham, Nottingham, UK.

10. Division of Informatics, Imaging & Data Sciences (L5), School of Health Sciences, Faculty of Biology, Medicine and Health, Health Innovation Manchester, University of Manchester, Manchester, UK.

11. Keele Cardiovascular Research group, Centre for Prognosis Research, Institute for Primary Care and Health Sciences, Stoke-on-Trent, UK.

Corresponding author. Salwa S Zghebi. Division of Population Health, Health Services Research and Primary Care, University of Manchester, Manchester, UK. Email address: Salwa.Zghebi@manchester.ac.uk

Number of words (Abstract): 300

For peer review only - http://bmjopen.bmj.com/site/about/guidelines.xhtml

Number of words (full text): 4,108
Number of Tables 1
Number of Figures: 4

For peer review only

For peer review only - http://bmjopen.bmj.com/site/about/guidelines.xhtml

Abstract

Introduction: The increasing prevalence of type 2 diabetes (T2DM) presents a significant burden on affected individuals and health-care systems internationally. There is, however, no agreed validated measure to infer diabetes severity from electronic health records (EHRs). We aim to quantify T2DM severity and validate it using clinical adverse outcomes.

Methods and Analysis: Primary care data from the Clinical Practice Research Datalink (CPRD), linked hospitalisation and mortality records between April-2007 and March-2017 for T2DM patients in England will be used to develop a clinical algorithm to grade T2DM severity. The EHR-based algorithm will incorporate main risk factors (severity domains) for adverse outcomes to stratify T2DM cohorts by baseline and longitudinal severity scores. Provisionally, T2DM severity domains, identified through a systematic review and expert opinion are: diabetes duration, HbA_{1c}, microvascular complications, comorbidities, and co-prescribed treatments. Severity scores will be developed by two approaches: i) calculating an aggregate score of severity domains; ii) through hierarchical stratification of complications. Regression models estimates will be used to calculate domains weights. Survival analysis for the association between weighted severity scores and future outcomes: cardiovascular events; hospitalisation (diabetes-related, cardiovascular); and mortality (diabetes-related, cardiovascular, all-cause mortality) will be performed as a statistical validation. The proposed EHR-based approach will quantify the T2DM severity for primary care performance management and inform the methodology for measuring severity of other primary care-managed chronic conditions. We anticipate that the developed algorithm will be a practical tool for practitioners, aid clinical management decision-making, inform stratified medicine, support future clinical trials and contribute to more effective service-planning and policy-making.

Ethics and Dissemination: The study protocol was approved by the Independent Scientific Advisory Committee (ISAC). Some data were presented at the NIHR SPCR Showcase, September-2017, Oxford,UK; the Diabetes UK Professional Conference March-2018, London,UK. The study findings will be disseminated in relevant academic conferences and peer-reviewed journals.

For peer review only - http://bmjopen.bmj.com/site/about/guidelines.xhtml

Strengths and limitations of this study

- This is the first UK-based study to develop a diabetes severity scoring tool based on real-world electronic healthcare data to grade people with type 2 diabetes by their clinical severity.
- The study will include a large sample size and use high-quality medical data routinely-collected from general practices with access to linked national hospitalisation and cause-specific mortality datasets.
- The association between the computed severity scores and future adverse outcomes (cardiovascular event, hospitalisation and mortality) will be used to validate the developed severity algorithm.
- There is a possibility to miss other severity indicators not recorded in the data sources used such as pharmacy dispensing data.
- Given that the linkage scheme is only available for consented English general practices, the study cohort will be restricted to eligible patients registered within England (form nearly 58% of total CPRD general practices).

For peer review only

Introduction

The worldwide prevalence of diabetes mellitus is increasing with World Health Organisation (WHO) estimating that over 422 million adults had diabetes in 2016 with a growth in the global prevalence from 4.7% in 1980 to 8.5% in 2014.[1,2] In the UK, the prevalence of type 2 diabetes (T2DM) has nearly doubled between 2004 and 2014.[3,4] People with diabetes have a higher risk for morbidity and mortality when compared to individuals without diabetes,[3,5] and hence the increasing prevalence presents a significant burden on health care resources. Diabetes management expenses are estimated to consume up to 11% of the total health care budget in the UK and USA.[6,7] Diabetes is a complex metabolic condition, of an increasing severity,[8,9] at individually varying levels, and progressive development of vascular complications and end organ damage over time. Diabetes is mainly managed in primary care in the UK,[10] overall Europe,[11] USA,[12] and Asia.[13,14] In the UK, nearly 75% of the diabetes-associated costs relate to management of diabetic complications.[6]

The 'severity' of clinical conditions can be conceptualised as a progression of the underlying disease process. Increasing disease severity and development of associated complications lead to greater treatment complexity and clinical impact. The severity of chronic conditions, such as diabetes, has not been widely considered despite the clinical relevance and its likely impact on health care systems, where few studies have assessed disease severity among patients with T2DM or have quantified temporal trends or scores of severity.[15,16]

Severity scores could be clinically useful, particularly in T2DM, as such a new summary score could contribute above what existing risk scores offer (e.g. moving beyond risk for death) and also be highly relevant for use in six main clinical and research areas: i) identifying complex patients with a higher need for future care; ii) identifying patients at early stages of disease (benchmarking); iii) providing information that will directly inform clinical care; iv) identifying trajectories in severity over time; v) supporting research, for example, by serving as an important diabetes-specific covariate to consider in analyses, similarly to the Charlson Comorbidity Index, or as an outcome; and vi) providing data that will inform resource allocation in health systems, such as the National Health Service (NHS).

For peer review only - http://bmjopen.bmj.com/site/about/guidelines.xhtml

Here, we describe the conceptual development of a T2DM severity algorithm model, using electronic health records (EHRs) on clinical consultations and treatments. Our aim is to use routinely-collected clinical and administrative data to develop a scoring tool which would quantify and grade the severity of T2DM. This model could potentially be used by clinicians to stratify patients based on disease severity and we aim to demonstrate potential advantages over similar risk scores. To validate the algorithm, our secondary aim is to examine the association of severity grades with risk of three main adverse outcomes: cardiovascular disease, hospitalisation, and mortality. The developed severity stratification algorithm is anticipated to have direct impact on clinical practice and have wider implications on service-planning and policy-making.

Methods and Analysis

Data Source

This study will use data from the Clinical Practice Research Datalink (CPRD). The CPRD is one of the world's largest EHR with anonymised primary care records from over 650 general practices over the UK.[(17)] Nearly 76% of all CPRD registered patients are in England.[(17)] CPRD data include clinical diagnoses, prescribed therapies, biochemical test results and referrals to healthcare services. The accuracy and completeness of diagnostic coding and validity of CPRD data, in clinical research, have been reported as excellent with positive predictive values (PPVs) of over 90% for nearly 14 conditions (including diabetes and cerebrovascular disease).[(18)] PPVs were defined as the proportion of CPRD diagnoses that were validated as true cases when compared to a gold standard such as GP questionnaire or primary care records.

Two metrics for research-quality data, recommended to be used by researchers, are available for CPRD records.[(17)] For patient-level data, the flag 'acceptable' indicates that a patient's record has met certain quality standards such as registration status, valid age and gender, and record of patient events. For practice-level data, the up-to-standard (UTS) date is used as a data quality measure. The UTS date is calculated for each CPRD general practice as the latest date at which the general practice meets minimum quality criteria based on

For peer review only - http://bmjopen.bmj.com/site/about/guidelines.xhtml

two central concepts: the continuity of recorded data and the number of recorded deaths, in comparison to an expected national range.[17,19]

Nearly 75% of general practices in England (approximately 58% of all CPRD practices) have consented to the CPRD Linkage Scheme for access to a number of linked datasets and national disease registries.[17] These include hospital data (including outpatient, admissions, and Accident and Emergency (A&E) data), mortality records (held by the Office for National Statistics (ONS)), socio-economic status and the cancer registry. The linked hospitalisation records, held by the Hospital Episode Statistics (HES), provide ethnicity data, admission and discharge dates, clinical diagnoses and procedures during hospitalisation recorded using the tenth revision of the International Classification of Diseases (ICD-10) and operating procedure codes (OPCS). This study will only include patients registered with English general practices which provide up-to-standard data and which participate in the CPRD Linkage Scheme.

Study Population

The study cohort will include individuals aged 35 years or over with T2DM (with ≥1 diagnostic code as in Table S1) between 1st April 2007 and 31st March 2017. Patients with an ever record of type 1 diabetes (with ever code for type 1 diabetes as in Table S2) will be excluded unless have records of non-insulin antidiabetic therapies. We chose the study period after 2006 as the quality of CPRD data has improved substantially to adhere with the then introduced important changes to the national incentive scheme The Quality and Outcomes Framework (QOF), an incentivisation programme for all GP surgeries in the UK.[20,21] QOF exception reporting process allows general practices to exclude patients from indicators or a clinical domain based on discretionary exception codes. However, evidence on the use of exception codes has shown that they are being used appropriately by practices and overall exception rates for diabetes patients are low.[22]

For this cohort construction, we will consider implementing previously-validated algorithms designed to identify diabetes cases by avoiding potential misclassification in routinely-collected data such as CPRD.[23,24] Eligible patients will be followed-up until censored at the

For peer review only - http://bmjopen.bmj.com/site/about/guidelines.xhtml

earliest instance of any of the following event dates: patient transferred out of the practice (any cause), last collection date for the practice, the study end on the 31st March 2017 or death. The main demographic and clinical characteristics of the defined diabetes cohorts (such as age, gender, geographic region within England, patient-level and general practice-level social deprivation, BMI, and baseline HbA_{1c}) will be identified. Based on our previous studies, the expected sample size in a year will include 11,000 T2DM patients registered with English general practices linked to the HES Admitted Patient Care dataset, HES Outpatient and HES A&E datasets. A random 90% of the identified diabetes cohort (training dataset) will be used to develop the severity tool, with the remaining 10% of the cohort used as a validation dataset, as described below.

Severity Domains

A systematic literature search for studies that developed algorithms or models to assess and quantify the severity of diabetes was conducted to identify the domains and sub-domains for T2DM severity (to be published separately). Also, expert clinical opinion from members of the research team was used to supplement the search process and identify possible omissions. Clinical members in the team include pharmacists (DMA, SSZ), general practitioners (CS, CM, CCG, HVm, and NQ), a consultant diabetologist (MKR) and a consultant cardiologist (MM), who used their expertise to create a list of relevant clinical domains for T2DM severity. The final domains to be included in the severity model will be decided during the analysis stage. Currently, the identified clinical domains that are relevant to the degree of progression of T2DM include: patient factors (diabetes duration (the period between T2DM diagnosis and the severity score estimation) and body mass index (BMI)), monitoring laboratory tests (glycated haemoglobin (HbA_{1c}) categories (threshold of 7% [53 mmol/mol]), blood glucose levels), type of antidiabetic therapy, other prescribed medications (such as lipid-regulating medications and angiotensin-converting enzyme inhibitors (ACEIs)), comorbidities (including diabetes-related complications, depression), hospitalisation, and surgical interventions. Comorbidities will be identified using appropriate code lists and contribute to the severity score according to each score computing method. The identified domains and sub-domains are described in Table 1. Domains will also be

For peer review only - http://bmjopen.bmj.com/site/about/guidelines.xhtml

reviewed by a panel of 'experts by experience' (people with lived experience of T2DM) to provide patient validation of the severity scoring tool.

The clinical Read codes for the defined severity domains codes and the product codes for drug therapies will be identified using the [pcdsearch] Stata user command.[25] The [pcdsearch] command is a search programme developed to extract code lists from typically very long lookup files associated with primary care databases using an input file containing a list of stubs for codes of interest to be searched for. For CPRD, the lookup files Medical dataset is searched for all clinical Read codes and the Product lookup file, that includes unique product codes, is searched for all treatments. The corresponding ICD-10 codes will be used to identify clinical domains recorded in the hospitalisation data and ONS mortality records. The relevant CPRD operational identification entities for the laboratory tests will be identified. This is an ongoing process whereby the final lists of domains and codes will be reached by consensus among the clinical members of the team. All code lists will be available on the online clinical code repository (ClinicalCodes.org).[26]

Study Outcomes

The adverse outcomes of interest will be the development of the first event among cardiovascular disease, (myocardial infarction, stroke), future hospitalisation (any hospitalisation, diabetes-related and cardiovascular hospitalisation) and death (diabetes-related mortality, cardiovascular mortality and all-cause mortality). Secondary outcome will be future hospitalisation due to hypoglycaemia, a relevant and potentially preventable adverse outcome. The hospitalisation and death outcomes will be identified using the linked HES and ONS mortality data, respectively. Similarly with severity domains, Read codes and ICD-10 codes (available on ClinicalCodes.org online repository) will be used to identify the outcomes as appropriate.

For peer review only - http://bmjopen.bmj.com/site/about/guidelines.xhtml

Statistical Analyses

Diabetes Severity Algorithm

Using annual data bins and grouping diabetes patients in the training dataset (include random 90% of the total diabetes cohort) from 1st April to 31st March between 2007 to 2017, the developed diabetes algorithm will grade the severity of T2DM using pre-defined (sub)domains. Our study period (after 2006) was selected to ensure very high data quality in primary care, while the addition of secondary care data will make our analyses even more robust, in terms of accurately classifying T2DM severity levels.

We will consider two approaches to derive numerical or categorical diabetes severity scores or levels. Firstly, we will use a binary classification (severity indicator: present/absent) within each sub-domain and calculate an aggregate score. The second approach, through hierarchical stratification of end organ microvascular and macrovascular complications, will involve increasing weighting within each sub-domain, as severity increases where scores of 1, 2, or 3 on each subdomain will be assigned based on clinical input in terms of severity. Then, regression model will be fitted from which the weights of its estimates will be used to calculate the weights for severity sub-domains. The highest possible severity score will be known when the final list of included domains and sub-domains is decided. Hierarchical diagrams of the clinical severity of identified diabetes-related complications domains (Figure 1) cerebrovascular (Figure 2), and cardiovascular (Figure 3, Figure 4, Figure S1, Figure S2) domains are presented.

Sub-domains with many measurements over time, such as HbA_{1c} levels and BMI, will be modelled as time-varying covariates (alternative models will be considered to account for non-linear changes). Within each time window the average of these measurements will be used, and we will also use multiple imputation approaches to account for missing data. Provisionally, we will consider using data records within look-back periods between 3-5 years. Different look-back windows within this period will be tested to obtain the optimal time window. Descriptive statistics of the study population and the estimated scores will be performed.

For peer review only - http://bmjopen.bmj.com/site/about/guidelines.xhtml

Severity Algorithm Validation

When we have developed a first version of the severity score tool, it will be validated statistically by quantifying the association of severity scores to future adverse outcomes. The primary outcome will be developing a cardiovascular event (myocardial infarction, stroke), future hospitalisation and death (diabetes-related mortality, cardiovascular mortality and all-cause mortality). A secondary adverse outcome will be future hospital admission due to hypoglycaemia, a relevant and potentially preventable adverse outcome. Cox proportional hazards (after assessing proportional hazards assumption) and/or competing-risks regressions will be used to perform survival analyses and assess the relationship between the severity score and outcomes controlling for all available patient characteristics (such as age, gender, ethnicity, and level of social deprivation). We aim to analyse and assess how severity scores differ across different levels of these variables. As stated earlier, a random 10% of the study cohort (validation dataset) will be used to validate the performance of the severity algorithm that was developed in the training dataset (90% of the diabetes cohort). Using the calculated subdomains weights (derived by the regression model estimates), Cox regression analysis will be used to validate the developed severity algorithm and we will assess the model performance using relevant measures (such as area under the receiver operating characteristic curve (AUROC). Using this analysis, we will also test what how much the 'sophisticated' full model that includes all relevant severity domains will add in terms of predictive validity of the adverse outcomes over a simpler model that includes demographic data (age, gender and level of social deprivation) alone.

Longitudinal Trends of Diabetes Severity

In newly diagnosed T2DM patients during the study period we will assess the temporal trends of severity scores over time by calculating the time needed to progress along the quintiles of the severity scale starting from the date or year of diagnosis and identify the predictors to this progression across quintiles.

For peer review only - http://bmjopen.bmj.com/site/about/guidelines.xhtml

While the inclusion of some domains such as carotid artery stenting, the use of ACEIs or lipid-regulating drugs, kidney transplantation and laser therapy are clinically-relevant and represent markers of greater level of disease severity they also improve the prognosis (lessen the grade of severity) i.e. raising a risk of "feedback". While we will further assess their role, we may have to not include these domains in the modelling at the analysis stage. All statistical analyses will be conducted using Stata Statistical Software for Windows: *Release 15* (College Station, TX: StataCorp LP, USA).

Patient and Public Involvement and Engagement (PPIE)

The public interest in the reuse of routinely-collected electronic health data for research purposes has expanded over recent years. Our study presents a valuable opportunity to address the challenge of how to develop meaningful and productive PPIE collaborations in observational studies that make use of secondary data.

We will identify suitable PPIE partners to collaborate on this study and develop creative strategies for meaningful involvement as the project develops. Participants to provide their input that will be particularly valuable in providing feedback into the diabetes severity algorithms, and deciding what information would be most useful to patients and how to be presented.

In the latter stages of the study, jointly with our PPIE partners, we will plan and deliver a patients' and carers' workshop where we will present emerging findings and seek feedback to inform further work and the dissemination strategy. This will include an exercise around weights of domains within the algorithm. PPIE work will be reported using the GRIPP (Guidance for Reporting Involvement of Patients and Public) checklist.[27]

For peer review only

For peer review only - http://bmjopen.bmj.com/site/about/guidelines.xhtml

Discussion

The global and country-specific prevalence of diabetes has increased substantially.[2,28-31] For T2DM, prevalence rates have doubled over the past two decades.[4,32,33] Diabetes diagnosis is associated with higher risk for morbidity and mortality in comparison to people without diabetes.[5] Despite the natural progression of most chronic diseases, including T2DM, and their important clinical implication on prognosis and medical resources utilisation, only a few studies have attempted to grade the severity of chronic conditions over time. Our proposed study aims to develop a contemporary algorithm to quantify the severity of T2DM, a highly prevalent condition, using routine EHR. The scoring tool will be based on clinically-relevant diagnoses and treatments modelled as severity domains and sub-domains. The algorithm will enable clinicians to grade T2DM patients according to the level of their disease severity, at baseline and longitudinally, as driven by the weights of the included clinical domains.

Prior Studies

Currently, there is limited data around validated severity measures that can be used in routine clinical practice managing patients with T2DM. Consequently, we are in parallel finalising a conducted systematic review for clinical-based diabetes severity models which will inform the refinement of this work. Here, we discuss three relevant diabetes severity measures that have been previously reported from countries outside the UK.

In the first study, Gini et al. (2016) categorised the severity of T2DM (N=300) into four levels based on insulin use and the presence of diabetic complications (Table S3).[15] A validation study in a random sample of cases was performed by interviewing their general practitioners. We however, aim to include more T2DM patients and consider more clinical data such as other coexisting conditions, non-insulin therapies, statins and ACEIs, that have been shown to be associated with reduced risk of adverse outcomes.

In the second study, a diabetes symptom checklist was created to measure the perceived symptom severity and assess the changes over time in 185 T2DM patients.[16] The checklist contained 34 items categorised by main clinical symptoms (Table S3). The patterns of

For peer review only - http://bmjopen.bmj.com/site/about/guidelines.xhtml

comorbidities and prescribed treatments were reportedly associated with significant differences in the estimated severity scores.[16] The sample size was however relatively small and the checklist was based on patients' perception on diabetes symptoms severity.

Thirdly, Young et al. (2008), calculated a diabetes complications severity index (DCSI) in 4,229 patients with type 1 or type 2 diabetes in one US geographic region to examine its association with adverse outcomes (risk of hospitalisation and mortality). In comparison to using a simple numerical count of complications, DCSI found to be a better tool to predict adverse outcomes.[34] The authors used pharmacy (insulin use only) and laboratory data to compute the severity index and included patients from clinics with largest ethnic diversity. However, the DCSI missed additional domains such as diabetes duration, hyperlipidaemia and a wider range of diabetes-related and end-organ damage manifestations that we aim to assess in addition to hospitalisation and mortality.

In the wider literature, although several studies have investigated the possible role of other various factors on the severity of diabetes, none have provided a severity scoring tool that utilises data from various clinical domains as planned in our study that can use the wealth of information routinely-collected in electronic healthcare databases. In these studies, approaches used to define diabetes severity included: comparing T2DM severity, before and after obesity surgery;[35] examining the association between diabetes severity and either: haematological and immunological changes,[36] levels of urine citrate,[37] a biomarker for adverse outcomes;[38] grip strength;[39] or the use of complementary medicine to manage T2DM.[40]

Overall, the severity of T2DM has been assessed using the following domains: the complexity of antidiabetic treatment regimens[35] or HbA_{1c} levels;[37] a summary severity variable that includes vascular complications;[41] or a health status composite, number of comorbidities and patterns of treatments.[42] Other putative and less clinically-robust indicators or animal models were reportedly used to assess the severity of T2DM such as the effect of different patient education approaches on diabetes severity;[43] evaluating the effect of parental history;[44] and the role of genetic,[45,46] metabolic,[47] or inflammatory mediators.[48]

For peer review only - http://bmjopen.bmj.com/site/about/guidelines.xhtml

In comparison to our planned study, none of previous studies included as many routinely-collected clinically-relevant variables as in our more inclusive summary severity score algorithm or assessed the association of estimated scores with the various adverse outcomes included in our defined primary and secondary endpoints.

Potential Strengths and Limitations

Our proposed study has several potential strengths: First, it aims to present a contemporary measure to the available tools and the first UK-based study to develop an EHR-based severity scoring tool to grade patients by their T2DM severity. Second, the study uses high-quality real-world medical data routinely-collected from general practices. The use of routinely-collected data indicates that severity scores can be generated automatically with minimal effort. Third, the views of PPIE collaborators will be incorporated in the development of the severity tool. Fourth, we will access two linked datasets: hospitalisation (HES) data (to maximise data capture and reduce condition misclassification), and cause-specific mortality data to ascertain causes of death. Fifth, the sample size is expected to be large enough to drive the development and evaluation of the severity algorithm. Sixth, a statistical validation of the developed algorithm is planned and described.

As we will use data available in CPRD and HES datasets, one of the limitations we anticipate is the possibility to miss other severity indicators not recorded in used datasets. These include detailed pharmacy data such as 'actual' dispensing and adherence data. Also, the use of routinely-collected data is associated with missing values, being collected from questionnaires, and issues around the accuracy of coding. However, we plan to use appropriate imputation methods and definite criteria to minimise the effect of coding issues. A possible limitation that should be acknowledged is underestimated and poorly represented T2DM severity levels for patients not regularly attending a general practice, people missing appointments or not being reliably captured in the database due to very high mobility status (e.g. homelessness). This limitation aligns with QOF exception reporting that allows practices to exclude patients from indicators or a clinical domain based on discretionary exception codes. However, the use of QOF exception coding was considered

For peer review only

For peer review only - http://bmjopen.bmj.com/site/about/guidelines.xhtml

appropriate and its levels were very low, especially for informed dissent.[22] Another limitation is that our study will be restricted to patients registered with general practices in England, as CPRD currently only provides linkage to external national datasets for consented general practices in England. However, English general practices form the majority of all CPRD practices including nearly 76% of total registered patients.[17] Finally, due to project time constrains, there is no possibility to validate the developed diabetes scores using questionnaires (as reported previously [15]) or replicating the algorithm in a separate dataset. However, we have planned a statistical validation of the developed algorithm.

Importance and the Clinical Implications of the Severity Tool

The developed algorithm and severity tool may have significant implications for primary care both in terms of disease management and resource allocation. Ideally, through future work of further validation and assessment of clinical utility, the severity tool will be of practical use in primary care through its implementation in the clinical computing systems used in the UK.[49] The clinical significance of the developed severity algorithm to primary care is driven by the inclusion of highly-relevant clinical domains, such as diabetes-related complications and comorbidities, mapped to routinely-collected data.

Additionally, by assessing the longitudinal patterns of severity, the developed tool may be more clinically relevant than the currently used proxy (HbA_{1c}), and thus it could be a more reliable indicator in informing practices' remuneration for diabetes care. Categorising individuals based on their diabetes severity will be relevant for risk stratification (which may enable safer delegation of care within the clinical team), identify individualised patient risks and will help practitioners triage patients in need of a greater clinical input which informs towards stratified medicine to reduce future life-changing diabetes-related complications. Moreover, the weights of the severity scores may inform future clinical trials as the scoring tool considers a broader range of cardiovascular conditions than in most randomised clinical trials. Given the relatively low rates of cardiovascular outcomes in some trials, identifying diabetes patients who are at higher risk via our severity algorithm would help to power trials with overall longer term benefit for patients. Finally, the composite severity score may

For peer review only - http://bmjopen.bmj.com/site/about/guidelines.xhtml

For peer review only

serve as an important confounding factor in future research, as for example, to match diabetes cases and controls in observational studies and clinical trials.

Conclusions

Despite the availability of rich routinely-collected electronic medical data, there are limited validated measures to quantify the severity of main chronic conditions managed in primary care utilising this important resource. We aim to develop a severity algorithm for type 2 diabetes to quantify disease severity using clinically-important domains derived from UK EHR. Our goal is that developed severity score data will provide clinicians with practical information that will influence management pathways. The findings could have important implications for service-planning and policy-making. The severity tool may provide a useful model and methodology towards the assessment of severity in other chronic medical conditions managed in primary care setting. Future research may involve further validation by testing the severity model in a separate primary care database.

Author Contributions

E.K. S.S.Z. M.M., M.K.R and H.V.m developed the study design and data analysis plan. S.S.Z. M.M., M.K.R and H.V.m agreed on provisional clinical code lists. S.S.Z. prepared the first draft of the manuscript, and E.K., M.M., M.K.R. and H.V.m critically reviewed initial versions. C.P. contributed to the planned PPIE work. D.R., C.C.G., C.M., N.P., D.M.A., C.S., N.Q., M.N. and S.W reviewed and critically edited the manuscript. All authors approved the final version of the protocol before submission. S.S.Z. is the guarantor.

Funding

This study is funded by the National Institute for Health Research School for Primary Care Research (NIHR SPCR), grant number 331. This report is independent research by the National Institute for Health Research. The views expressed in this publication are those of the authors and necessarily those of the NHS, the National Institute for Health Research or the Department of Health.

For peer review only - http://bmjopen.bmj.com/site/about/guidelines.xhtml

Competing Interests

E.K., H.V.m, M.M., D.R., C.C.G. C.P., C.M., N.P., M.N. declare no competing interests. S.S.Z. reports support by the NIHR SPCR during this study. D.M.A. has received grant funding from Abbvie and has served on advisory boards for Pfizer and GSK. M.K.R. has received educational grant support from MSD and Novo Nordisk; has modest stock ownership in GSK; and has consulted for Roche. N.Q. reports grants from the NIHR SPCR, during the conduct of the study. C.S. reports grants from NIHR SPCR, during the conduct of the study; grants from NHS CLAHRC West, grants from Avon Primary Care Research Collaborative, outside the submitted work. S.W. serves as a member of the Clinical Practice Research Datalink Independent Scientific Committee (ISAC) at the UK Medicines and Health Regulatory Agency.

Ethical Approval

The approval for the study protocol was obtained from the CPRD's Independent Scientific Advisory Committee (ISAC) (Reference: 17_168RAR2, February 2018).

List of Abbreviations

ACEI: Angiotensin converting enzyme inhibitors; **CABG**: coronary artery bypass graft; **CHD**: coronary heart disease; **CKD**: chronic kidney disease; **CPRD**: Clinical Practice Research Datalink; **CRT**: cardiac resynchronization therapy; **DCSI**: diabetes complications severity index; **EHR**: electronic health record; **EVAR**: Endovascular aneurysm repair; **GRIPP**: Guidance for Reporting Involvement of Patients and Public; **HbA_{1c}**: glycated haemoglobin; **HES**: Hospital Episode Statistics; **HES A&E:** HES Accident & Emergency; **HF**: heart failure; **ICD**: implantable cardioverter defibrillator; **ICD-10**: the 10th revision of the International Classification of Diseases; **ISAC**: Independent Scientific Advisory Committee; **MI**: myocardial infarction; **NHS:** National Health Service; **ONS**: Office for National Statistics; **OPCS**: operating procedure codes; **PAD**: peripheral artery disease; **PCI**: percutaneous coronary intervention; **PPIE:** patient and public involvement and engagement; **PTCA**: percutaneous transluminal coronary angioplasty; **PVD**: peripheral vascular disease; **QOF**: Quality and Outcomes Framework; **SVT**: supraventricular tachycardia; **TAVI**: transcatheter aortic valve

For peer review only - http://bmjopen.bmj.com/site/about/guidelines.xhtml

implantation; **T2DM**: type 2 diabetes; **TIA**: transient ischemic attack; **VF**: ventricular fibrillation; **VT**: ventricular tachycardia; **WHO**: World Health Organization.

For peer review only

For peer review only - http://bmjopen.bmj.com/site/about/guidelines.xhtml

References

1. World Health Organization (WHO) media centre. Diabetes. [Fact sheet] 2016 [cited 2016 29 April]; Available from: http://www.who.int/mediacentre/factsheets/fs312/en/.
2. World Health Organization (WHO). Global Report on Diabetes. 2016 [cited 2017 11 February]; Available from: http://apps.who.int/iris/bitstream/10665/204871/1/9789241565257_eng.pdf.
3. Zghebi SS, Steinke DT, Carr MJ, Rutter MK, Emsley RA, Ashcroft DM. Examining Trends in Type 2 Diabetes Incidence, Prevalence and Mortality in the UK between 2004 and 2014. Diabetes, Obesity and Metabolism. 2017.
4. Sharma M, Nazareth I, Petersen I. Trends in incidence, prevalence and prescribing in type 2 diabetes mellitus between 2000 and 2013 in primary care: a retrospective cohort study. BMJ Open. 2016;6:e010210.
5. American Diabetes Association (ADA). Standards of Medical Care in Diabetes - 2015. Diabetes Care. 2015;38(Suppl 1):S1-S49.
6. Hex N, Bartlett C, Wright D, Taylor M, Varley D. Estimating the current and future costs of Type 1 and Type 2 diabetes in the UK, including direct health costs and indirect societal and productivity costs. Diabetic Medicine. 2012;29:855-62.
7. Walker BR, Colledge NR, Ralston SH, Penman ID. Davidson's Principles and Practice of Medicine. China: Elsevier 2014 [cited 2016 30 June]. Available from: http://www.elsevier-etextbooks.com/pdfreader/davidsons-principles-practice-medicine57814.
8. Walker BR, Colledge NR, Ralston SH, Penman ID. Davidson's Principles and Practice of Medicine: Elsevier; 2014 [cited 2017 11 May]. Available from: http://elsevierelibrary.co.uk/pdfreader/davidsons-principles-practice-medicine57814.
9. Nefs G, Pop VJM, Denollet J, Pouwer F. The Longitudinal Association between Depressive Symptoms and Initiation of Insulin Therapy in People with Type 2 Diabetes in Primary Care. Plos One. 2013;8(11).
10. Murrells T, Ball J, Cookson G, Maben J, Lee G, Griffiths P. Managing diabetes in primary care: how does the configuration of the workforce affect quality of care? National Nursing Research Unit, King's College London, 2013 November 2013. Report No.: Department of Health Policy Research Programme, ref. 016/0058.
11. Luk VE, Patricia S, Luc F, Liesbeth B, Jan DM. Priorities for diabetes primary care in Europe. Primary Care Diabetes. 2008;2(1):3-8.
12. Spann SJ, Nutting PA, Galliher JM, Peterson KA, Pavlik VN, Dickinson LM, et al. Management of Type 2 Diabetes in the Primary Care Setting: A Practice-Based Research Network Study. Ann Fam Med. 2006;4(1):23–31.
13. Tai T-Y, Chuang L-M, Tsai S-T, Huang B-Y, The Diabcare (Taiwan) Study Group. Treatment of Type 2 Diabetes Mellitus in a Primary Care Setting in Taiwan: Comparison with Secondary/Tertiary Care. Journal of the Formosan Medical Association = Taiwan yi zhi. 2006;105(2):105–17.
14. Mafauzy M. Diabetes control and complications in private primary healthcare in Malaysia. Med J Malaysia. 2005;60(2):212-7.
15. Gini R, Schuemie MJ, Mazzaglia G, Lapi F, Francesconi P, Pasqua A, et al. Automatic identification of type 2 diabetes, hypertension, ischaemic heart disease, heart failure and their levels of severity from Italian General Practitioners' electronic medical records: a validation study. BMJ open. 2016;6(12):e012413. Epub 2016/12/13.
16. Grootenhuis PA, Snoek FJ, Heine RJ, Bouter LM. Development of a type 2 diabetes symptom checklist: a measure of symptom severity. Diabetic medicine : a journal of the British Diabetic Association. 1994;11(3):253-61. Epub 1994/04/01.
17. Herrett E, Gallagher AM, Bhaskaran K, Forbes H, Mathur R, van Staa TP, et al. Data Resource Profile: Clinical Practice Research Datalink (CPRD). International Journal of Epidemiology. 2015;44(3):827-36.

For peer review only - http://bmjopen.bmj.com/site/about/guidelines.xhtml

18. Khan NF, Harrison SE, Rose PW. Validity of diagnostic coding within the General Practice Research Database: a systematic review. British Journal of General Practice. 2010;60(572):e128-e36.
19. Williams T, van Staa T, Puri S, Eaton S. Recent advances in the utility and use of the General Practice Research Database as an example of a UK Primary Care Data resource. Therapeutic Advances in Drug Safety. 2012;3(2):89-99.
20. Lester H, Campbell S. Developing Quality and Outcomes Framework (QOF) indicators and the concept of 'QOFability'. Quality in Primary Care. 2010;18:103-9.
21. Calvert M, Shankar A, McManus RJ, Lester H, Freemantle N. Effect of the quality and outcomes framework on diabetes care in the United Kingdom: retrospective cohort study. BMJ. 2009;338:b1870.
22. Doran T, Kontopantelis E, Fullwood C, Lester H, Valderas JM, Campbell S. Exempting dissenting patients from pay for performance schemes: retrospective analysis of exception reporting in the UK Quality and Outcomes Framework. BMJ. 2012;344:e2405.
23. de Lusignan S, Khunti K, Belsey J, Hattersley A, van Vlymen J, Gallagher H, et al. A method of identifying and correcting miscoding, misclassification and misdiagnosis in diabetes: a pilot and validation study of routinely collected data. Diabetic Medicine. 2010;27(2):203-9.
24. Wright AK, Kontopantelis E, Emsley R, Buchan I, Sattar N, Rutter MK, et al. Life Expectancy and Cause-Specific Mortality in Type 2 Diabetes: A Population-Based Cohort Study Quantifying Relationships in Ethnic Subgroups. Diabetes Care. 2017;40(3):338-45.
25. Olier I, Springate DA, Ashcroft DM, Doran T, Reeves D, Planner C, et al. Modelling Conditions and Health Care Processes in Electronic Health Records: An Application to Severe Mental Illness with the Clinical Practice Research Datalink. Plos One. 2016;11(2):e0146715.
26. Springate DA, Kontopantelis E, Ashcroft DM, Olier I, Parisi R, Chamapiwa E, et al. ClinicalCodes: An Online Clinical Codes Repository to Improve the Validity and Reproducibility of Research Using Electronic Medical Records. PLoS ONE. 2014;9(6):e99825.
27. Staniszewska S, Brett J, Mockford C, Barber R. The GRIPP checklist: Strengthening the quality of patient and public involvement reporting in research. International Journal of Technology Assessment in Health Care. 2011;27(4):391-9.
28. Lipscombe LL, Hux JE. Trends in diabetes prevalence, incidence, and mortality in Ontario, Canada 1995–2005: a population-based study. Lancet. 2007;369:750-56.
29. Onat A, Hergenc G, Uyarel H, Can G, Ozhan H. Prevalence, incidence, predictors and outcome of type 2 diabetes in Turkey. Anadolu kardiyoloji dergisi : AKD = the Anatolian journal of cardiology. 2006;6(4):314-21.
30. International Diabetes Federation (IDF). IDF Diabetes Atlas - 7th Edition. 2015 [cited 2017 08 February]; Available from: http://www.diabetesatlas.org/.
31. Lin CC, Li CI, Hsiao CY, Liu CS, Yang SY, Lee CC, et al. Time trend analysis of the prevalence and incidence of diagnosed type 2 diabetes among adults in Taiwan from 2000 to 2007: a population-based study. BMC Public Health. 2013;13(318).
32. Gonzalez ELM, Johansson S, Wallander MA, Rodriguez LAG. Trends in the prevalence and incidence of diabetes in the UK: 1996-2005. J Epidemiol Community Health. 2009;63(4):332-6.
33. Evans JMM, Barnett KN, Ogston SA, Morris AD. Increasing prevalence of type 2 diabetes in a Scottish population: effect of increasing incidence or decreasing mortality? Diabetologia. 2007;50(4):729-32.
34. Young BA, Lin E, Von Korff M, Simon G, Ciechanowski P, Ludman EJ, et al. Diabetes complications severity index and risk of mortality, hospitalization, and healthcare utilization. Am J Manag Care. 2008;14(1):15-24.
35. Runkel M, Muller S, Brydniak R, Runkel N. Downgrading of type 2 diabetes mellitus (T2DM) after obesity surgery: duration and severity matter. Obesity surgery. 2015;25(3):494-9. Epub 2014/09/12.

For peer review only - http://bmjopen.bmj.com/site/about/guidelines.xhtml

36. Okano K, Araki M, Yamamoto M, Ishikawa T, Ichihara K, Yamada O. Exploration of hematological and immunological changes associated with the severity of type 2 diabetes mellitus in Japan. Nursing & health sciences. 2008;10(1):65-9. Epub 2008/02/09.
37. Fram EB, Moazami S, Stern JM. The Effect of Disease Severity on 24-Hour Urine Parameters in Kidney Stone Patients With Type 2 Diabetes. Urology. 2016;87:52-9. Epub 2015/11/04.
38. Eldor R, Klieger Y, Sade-Feldman M, Vaknin I, Varfolomeev I, Fuchs C, et al. CD247, a novel T cell-derived diagnostic and prognostic biomarker for detecting disease progression and severity in patients with type 2 diabetes. Diabetes care. 2015;38(1):113-8. Epub 2014/11/05.
39. Loprinzi PD, Loenneke JP. Evidence of a Link Between Grip Strength and Type 2 Diabetes Prevalence and Severity Among a National Sample of U.S. Adults. Journal of physical activity & health. 2016;13(5):558-61. Epub 2015/11/10.
40. Nahin RL, Byrd-Clark D, Stussman BJ, Kalyanaraman N. Disease severity is associated with the use of complementary medicine to treat or manage type-2 diabetes: data from the 2002 and 2007 National Health Interview Survey. BMC complementary and alternative medicine. 2012;12:193. Epub 2012/10/24.
41. Linzer M, Pierce C, Lincoln E, Miller DR, Payne SM, Clark JA, et al. Preliminary validation of a patient-based self-assessment measure of severity of illness in type 2 diabetes: results from the pilot phase of the Veterans Health Study. The Journal of ambulatory care management. 2005;28(2):167-76. Epub 2005/06/01.
42. Gatlin PK, Insel KC. Severity of Type 2 Diabetes, Cognitive Function, and Self-Care. Biological research for nursing. 2015;17(5):540-8. Epub 2014/11/14.
43. Windrum P, Garcia-Goni M, Coad H. The Impact of Patient-Centered versus Didactic Education Programs in Chronic Patients by Severity: The Case of Type 2 Diabetes Mellitus. Value in health : the journal of the International Society for Pharmacoeconomics and Outcomes Research. 2016;19(4):353-62. Epub 2016/06/23.
44. Svensson E, Berencsi K, Sander S, Mor A, Rungby J, Nielsen JS, et al. Association of parental history of type 2 diabetes with age, lifestyle, anthropometric factors, and clinical severity at type 2 diabetes diagnosis: results from the DD2 study. Diabetes/metabolism research and reviews. 2016;32(3):308-15. Epub 2015/09/27.
45. Powell DS, Maksoud H, Charge SB, Moffitt JH, Desai M, Da Silva Fihlo RL, et al. Apolipoprotein E genotype, islet amyloid deposition and severity of Type 2 diabetes. Diabetes research and clinical practice. 2003;60(2):105-10. Epub 2003/04/23.
46. Jiang Q, Lyu XM, Yuan Y, Wang L. Plasma miR-21 expression: an indicator for the severity of Type 2 diabetes with diabetic retinopathy. Bioscience reports. 2017;37(2). Epub 2017/01/22.
47. Campbell-Tofte J, Hansen HS, Mu H, Molgaard P. Increased lipids in non-lipogenic tissues are indicators of the severity of type 2 diabetes in mice. Prostaglandins, leukotrienes, and essential fatty acids. 2007;76(1):9-18. Epub 2006/11/14.
48. Garcia-Elorriaga G, Padilla-Reyes M, Cruz-Olivo F, Fuentes-Allen JL, del Rey-Pineda G, Gonzalez-Bonilla C. [Pro-inflammatory cytokines related to severity and mortality in type 2 diabetes patients with soft tissue infection]. Revista medica del Instituto Mexicano del Seguro Social. 2012;50(3):237-41. Epub 2012/11/28. Citocinas, diabetes e infeccion de tejidos blandos. Su relacion con la severidad y mortalidad.
49. Kontopantelis E, Buchan I, Reeves D, Checkland K, Doran T. Relationship between quality of care and choice of clinical computing system: retrospective analysis of family practice performance under the UK's quality and outcomes framework. BMJ open. 2013;3:e003190.
50. Dunning T, Martin M. Type 2 diabetes: is it serious? Journal of Diabetes Nursing. 1998;2(3):70-6.

For peer review only - http://bmjopen.bmj.com/site/about/guidelines.xhtml

Table 1 The main (sub) domains identified to quantify the severity of type 2 diabetes

	Severity domain	Severity sub-domain
1.	**Risk factors***	• Duration of type 2 diabetes[40]<br>• Body mass index (BMI)<br>• Hypertension<br>• Hyperlipidaemia<br>• Personal/Family history of cardiovascular disease<br>• Blood glucose levels<br>○ Glycated haemoglobin (HbA_{1c})[35]<br>○ Fasting (FBG) and random (RBG) blood glucose
2.	**Type/pattern of antidiabetic treatment, insulin use and other therapies**	• Antidiabetic therapy ever;[40] Therapies with cardiovascular benefits versus other; Changes in drug treatments;[50] or the number of prescribed treatments[42]<br>• Insulin use: prescription ever or within 1 year of diagnosis; Insulin initiation:[15] time to initiation<br>• Other therapies: angiotensin converting enzyme inhibitors (ACEI) and lipid-regulating therapies
3.	**Microvascular diabetic complications**[15,34]	• Neuropathy (Foot ulcer, Charcot foot, gangrene, amputation)<br>• Nephropathy<br>• Retinopathy (laser therapy and blindness)
4.	**Renal disease**	• Microalbuminuria and proteinuria<br>• Moderate-severe chronic kidney disease (CKD) stages 3 and 4[34]<br>• End-stage renal disease (ESRD): kidney transplant and dialysis
5.	**Cardiovascular and Cerebrovascular disease**	• Atherosclerosis[15,34]<br>• Myocardial infarction (MI) [15,34]<br>• Angina[15,34]<br>• Atrial/ventricular fibrillation (AF)/(VF)[34]<br>• Heart valve disease<br>• Heart failure (HF)[34]<br>• Peripheral vascular disease (PVD)[34]<br>• Transient ischemic attack (TIA)<br>• Ischaemic stroke, haemorrhagic stroke [15,34]
6.	**Cardiovascular and Cerebrovascular interventions**	• Coronary artery bypass graft (CABG)<br>• Coronary artery interventions (PCI/PTCA)<br>• Endovascular aneurysm repair (EVAR)<br>• PVD stenting and bypass procedures<br>• Heart valve interventions<br>• Use of defibrillator<br>• Carotid artery events, stenting and bypass interventions
7.	**Other comorbidities**	• Anxiety<br>• Depression<br>• Dementia<br>• Cognitive impairment
8.	**Hospital admissions**	• Any-cause hospital admissions<br>• Diabetes-attributable admission<br>• Cardiovascular disease-related admission
9.	**Emergency diabetes-related event**	• Hypoglycaemia<br>• Hyperosmolar hyperglycaemic state (HHS)[34]<br>• Diabetic ketoacidosis (DKA) or other coma[34]

**Other demographic data (such as age, gender and the level of deprivation) are important predictors for adverse outcomes and will be included in the later risk-prediction analysis.*

For peer review only - http://bmjopen.bmj.com/site/about/guidelines.xhtml

Figure 1 Severity hierarchy of microvascular diabetic complications

Figure 2 Severity hierarchy of cerebrovascular domains

Figure 3 Severity hierarchy of coronary and vascular domains

Figure 4 Severity hierarchy of cardiovascular disease domains (heart rate, rhythm and conduction)

For peer review only

For peer review only - http://bmjopen.bmj.com/site/about/guidelines.xhtml

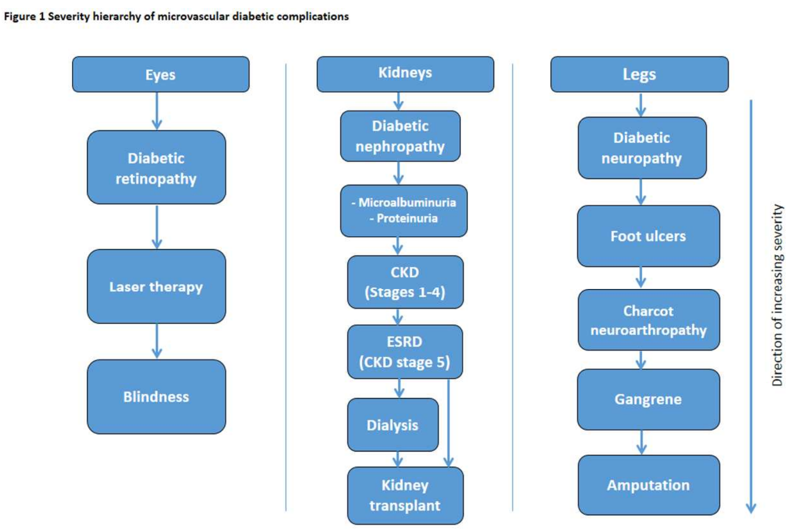

Figure 1 Severity hierarchy of microvascular diabetic complications

338x190mm (300 x 300 DPI)

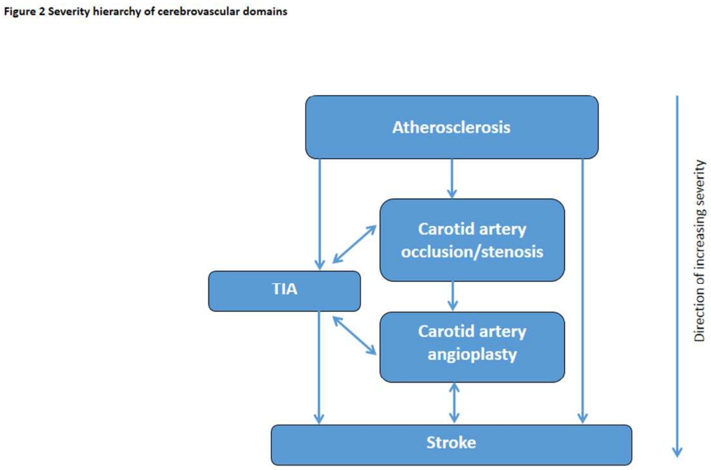

Figure 2 Severity hierarchy of cerebrovascular domains

338x190mm (300 x 300 DPI)

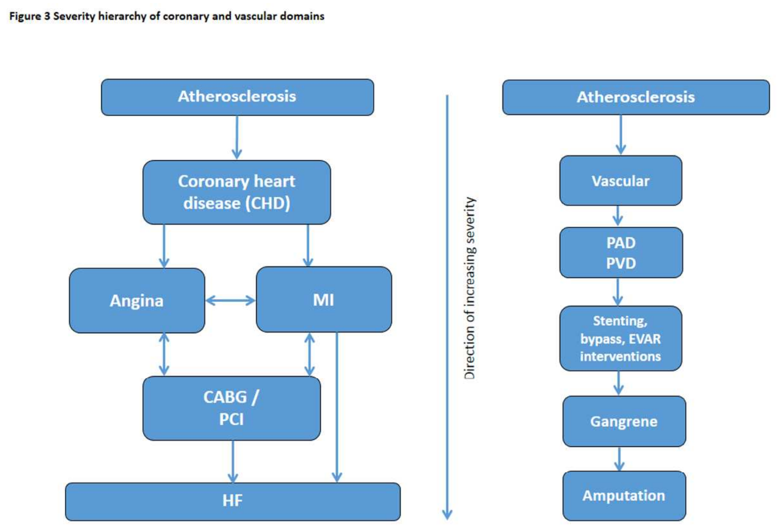

Figure 3 Severity hierarchy of coronary and vascular domains

338x190mm (300 x 300 DPI)

review only

Figure 4 Severity hierarchy of cardiovascular disease domains (heart rate, rhythm and conduction)

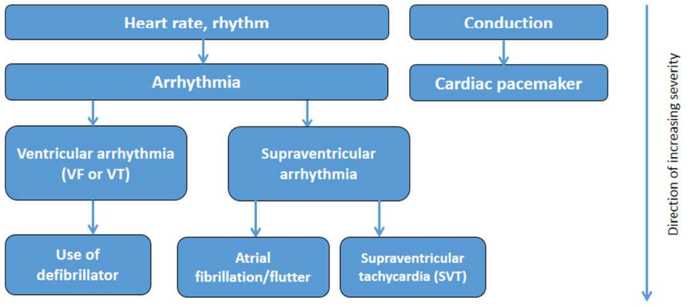

Figure 4 Severity hierarchy of cardiovascular disease domains (heart rate, rhythm and conduction)

338x190mm (300 x 300 DPI)

Supplementary material to paper "**Using electronic health records to quantify and stratify the severity of type 2 diabetes in primary care in England: rationale and cohort study design**" Zghebi et al. 2017

Supplementary data

Table S1 Inclusion medical codes for type 2 diabetes

	Medical code	Read term
**1.**	506	Non-insulin dependent diabetes mellitus
**2.**	758	Type 2 diabetes mellitus
**3.**	1407	Insulin treated Type 2 diabetes mellitus
**4.**	4513	Non-insulin dependent diabetes mellitus
**5.**	5884	NIDDM - Non-insulin dependent diabetes mellitus
**6.**	8403	Non-insulin dependent diabetes mellitus - poor control
**7.**	12640	Type 2 diabetes mellitus with nephropathy
**8.**	12736	Type 2 diabetes mellitus with gangrene
**9.**	17262	Non-insulin-dependent diabetes mellitus with retinopathy
**10.**	17859	Type 2 diabetes mellitus
**11.**	18143	Type II diabetes mellitus with arthropathy
**12.**	18209	Type 2 diabetes mellitus with renal complications
**13.**	18219	Type II diabetes mellitus
**14.**	18264	Insulin treated Type II diabetes mellitus
**15.**	18278	Insulin treated Type 2 diabetes mellitus
**16.**	18390	Type 2 diabetes mellitus with persistent microalbuminuria
**17.**	18425	Type 2 diabetes mellitus with polyneuropathy
**18.**	18496	Type 2 diabetes mellitus with retinopathy
**19.**	18777	Type 2 diabetes mellitus with renal complications
**20.**	22884	Type II diabetes mellitus
**21.**	24458	Type II diabetes mellitus - poor control
**22.**	24693	Non-insulin dependent diabetes mellitus with arthropathy
**23.**	24836	Type 2 diabetes mellitus with nephropathy
**24.**	25041	Dietary advice for type II diabetes
**25.**	25591	Type 2 diabetes mellitus with exudative maculopathy
**26.**	25627	Type 2 diabetes mellitus - poor control
**27.**	26054	Type 2 diabetes mellitus with persistent proteinuria
**28.**	29979	Non-insulin-dependent diabetes mellitus without complication
**29.**	32627	Type 2 diabetes mellitus with ketoacidosis
**30.**	34268	Type 2 diabetes mellitus with neurological complications
**31.**	34450	Hyperosmolar non-ketotic state in type 2 diabetes mellitus
**32.**	34912	Non-insulin dependent diabetes mellitus with ulcer
**33.**	35385	Type 2 diabetes mellitus with neuropathic arthropathy
**34.**	36633	Hyperosmolar non-ketotic state in type 2 diabetes mellitus
**35.**	37648	Insulin treated non-insulin dependent diabetes mellitus
**36.**	37806	Type 2 diabetes mellitus with peripheral angiopathy
**37.**	40401	Non-insulin dependent diabetes mellitus with gangrene
**38.**	42762	Type 2 diabetes mellitus with retinopathy
**39.**	43227	Type II diabetes mellitus with multiple complications
**40.**	43785	Non-insulin dependent diabetes mellitus with hypoglyca coma
**41.**	44779	Type 2 diabetes mellitus with diabetic cataract
**42.**	44982	Type 2 diabetes mellitus with diabetic cataract
**43.**	45467	Non-insulin dependent diabetes mellitus with polyneuropathy
**44.**	45913	Type 2 diabetes mellitus - poor control
**45.**	45919	Type 2 diabetes mellitus with neurological complications

For peer review only - http://bmjopen.bmj.com/site/about/guidelines.xhtml

Supplementary material to paper "**Using electronic health records to quantify and stratify the severity of type 2 diabetes in primary care in England: rationale and cohort study design**" Zghebi et al. 2017

**46.**	46150	Type 2 diabetes mellitus with gangrene
**47.**	46917	Type 2 diabetes mellitus with hypoglycaemic coma
**48.**	47315	Type II diabetes mellitus - poor control
**49.**	47321	Type 2 diabetes mellitus with ophthalmic complications
**50.**	47409	Type II diabetes mellitus with polyneuropathy
**51.**	47816	Type II diabetes mellitus with neuropathic arthropathy
**52.**	47954	Type 2 diabetes mellitus without complication
**53.**	48192	Type II diabetes mellitus with diabetic cataract
**54.**	49074	Type 2 diabetes mellitus with ulcer
**55.**	49655	Type II diabetes mellitus with retinopathy
**56.**	49869	Type 2 diabetes mellitus with arthropathy
**57.**	50225	Type II diabetes mellitus with renal complications
**58.**	50429	Non-insulin-dependent diabetes mellitus with ophthalm comps
**59.**	50527	Type II diabetes mellitus with polyneuropathy
**60.**	50609	Pre-existing diabetes mellitus, non-insulin-dependent
**61.**	50813	Type II diabetes mellitus with mononeuropathy
**62.**	51756	Type 2 diabetes mellitus with ketoacidotic coma
**63.**	52303	Non-insulin-dependent diabetes mellitus with renal comps
**64.**	53392	Type II diabetes mellitus without complication
**65.**	54899	Type II diabetes mellitus with peripheral angiopathy
**66.**	55075	Type II diabetes mellitus with ulcer
**67.**	55842	Non-insulin-dependent diabetes mellitus with neuro comps
**68.**	56268	Type II diabetes mellitus with hypoglycaemic coma
**69.**	57278	Type II diabetes mellitus with renal complications
**70.**	58604	Type II diabetes mellitus with retinopathy
**71.**	59253	Type 2 diabetes mellitus with arthropathy
**72.**	59365	Non-insulin dependent diabetes mellitus with nephropathy
**73.**	59725	Type II diabetes mellitus with ophthalmic complications
**74.**	60699	Type 2 diabetes mellitus with peripheral angiopathy
**75.**	60796	Type II diabetes mellitus with persistent proteinuria
**76.**	61071	Type 2 diabetes mellitus with hypoglycaemic coma
**77.**	62107	Type II diabetes mellitus with gangrene
**78.**	62146	Non-insulin-dependent diabetes mellitus with multiple comps
**79.**	62674	Type 2 diabetes mellitus with mononeuropathy
**80.**	63690	Type 2 diabetes mellitus with gastroparesis
**81.**	64571	Type II diabetes mellitus with nephropathy
**82.**	64668	Insulin treated Type II diabetes mellitus
**83.**	65267	Type 2 diabetes mellitus with multiple complications
**84.**	65704	Type 2 diabetes mellitus with ulcer
**85.**	66965	Type 2 diabetes mellitus with neuropathic arthropathy
**86.**	67905	Type II diabetes mellitus with neurological complications
**87.**	69278	Non-insulin depend diabetes mellitus with diabetic cataract
**88.**	70316	Type 2 diabetes mellitus with ophthalmic complications
**89.**	72320	Non-insulin dependent diabetes mellitus with mononeuropathy
**90.**	83532	Diabetes type 2 review
**91.**	85991	Type II diabetes mellitus with persistent microalbuminuria
**92.**	91646	Type II diabetes mellitus with ulcer
**93.**	93727	Type II diabetes mellitus with diabetic cataract
**94.**	95351	Type II diabetes mellitus with mononeuropathy
**95.**	98616	Type II diabetes mellitus with neurological complications
**96.**	98723	Type II diabetes mellitus with hypoglycaemic coma
**97.**	100964	Type II diabetes mellitus with ophthalmic complications
**98.**	101801	Type II diabetic dietary review
**99.**	102201	Type II diabetes mellitus with nephropathy

For peer review only - http://bmjopen.bmj.com/site/about/guidelines.xhtml

Supplementary material to paper "**Using electronic health records to quantify and stratify the severity of type 2 diabetes in primary care in England: rationale and cohort study design**" Zghebi et al. 2017

**100.**	102611	Type 2 diabetic dietary review
**101.**	103902	Type II diabetes mellitus with arthropathy
**102.**	104323	Type II diabetes mellitus with gangrene
**103.**	104639	Type II diabetes mellitus with peripheral angiopathy
**104.**	105784	Type 2 diabetes mellitus without complication
**105.**	106061	Type II diabetes mellitus with ketoacidotic coma
**106.**	106528	Type II diabetes mellitus with ketoacidosis
**107.**	107701	Hyperosmolar non-ketotic state in type II diabetes mellitus
**108.**	107824	Type II diabetes mellitus in remission
**109.**	108005	Type 2 diabetes mellitus with multiple complications
**110.**	109103	Type II diabetes mellitus without complication
**111.**	109197	Type II diabetes mellitus with neuropathic arthropathy
**112.**	109865	Type 2 diabetes mellitus with polyneuropathy

For peer review only

Supplementary material to paper "**Using electronic health records to quantify and stratify the severity of type 2 diabetes in primary care in England: rationale and cohort study design**" **Zghebi et al. 2017**

Table S2 Exclusion type 1 diabetes codes

	Medical code	Read term
**1.**	1038	Insulin dependent diabetes mellitus
**2.**	1549	Type 1 diabetes mellitus
**3.**	1647	Insulin dependent diabetes mellitus
**4.**	6509	Insulin dependent diabetes mellitus with retinopathy
**5.**	6791	Insulin dependent diabetes mellitus - poor control
**6.**	10418	Type 1 diabetes mellitus with nephropathy
**7.**	10692	Type 1 diabetes mellitus with ketoacidosis
**8.**	12455	Type I diabetes mellitus
**9.**	17545	Type I diabetes mellitus with diabetic cataract
**10.**	17858	Type 1 diabetes mellitus
**11.**	18230	Type 1 diabetes mellitus with neuropathic arthropathy
**12.**	18387	Type 1 diabetes mellitus with retinopathy
**13.**	18505	IDDM-Insulin dependent diabetes mellitus
**14.**	18642	Type 1 diabetes mellitus with arthropathy
**15.**	18683	Type 1 diabetes mellitus with ulcer
**16.**	21983	Type 1 diabetes mellitus with renal complications
**17.**	22871	Type 1 diabetes mellitus with exudative maculopathy
**18.**	24423	Type I diabetes mellitus
**19.**	24490	Diabetes mellitus, juvenile type, no mention of complication
**20.**	24694	Insulin dependent diabetes mellitus with mononeuropathy
**21.**	26855	Unstable insulin dependent diabetes mellitus
**22.**	30294	Type 1 diabetes mellitus with persistent microalbuminuria
**23.**	30323	Type 1 diabetes mellitus with persistent proteinuria
**24.**	31310	Insulin dependent diabetes maturity onset
**25.**	32359	Perceived control of insulin-dependent diabetes
**26.**	35288	Type 1 diabetes mellitus - poor control
**27.**	38161	Type I diabetes mellitus with retinopathy
**28.**	39070	Type 1 diabetes mellitus with hypoglycaemic coma
**29.**	39809	Insulin dependent diab mell with neuropathic arthropathy
**30.**	40023	Diabetes mellitus, juvenile type, with hyperosmolar coma
**31.**	40682	Type 1 diabetes mellitus maturity onset
**32.**	40837	Type 1 diabetes mellitus with ketoacidotic coma
**33.**	41049	Type 1 diabetes mellitus with retinopathy
**34.**	41716	Insulin dependent diabetes mellitus with polyneuropathy
**35.**	42567	Diabetes mellitus, juvenile type, with ketoacidotic coma
**36.**	42729	Type I diabetes mellitus with hypoglycaemic coma
**37.**	42831	Type 1 diabetes mellitus with neurological complications
**38.**	43921	Unstable type 1 diabetes mellitus
**39.**	44260	Insulin dependent diabetes mellitus with diabetic cataract
**40.**	44440	Insulin dependent diabetes mellitus with hypoglycaemic coma
**41.**	44443	Insulin dependent diabetes mellitus with ulcer
**42.**	45276	Insulin dependent diabetes mellitus with multiple complicat
**43.**	45914	Type 1 diabetes mellitus - poor control
**44.**	46301	Type 1 diabetes mellitus with polyneuropathy
**45.**	46850	Type I diabetes mellitus - poor control
**46.**	46963	Insulin-dependent diabetes mellitus with renal complications

For peer review only - http://bmjopen.bmj.com/site/about/guidelines.xhtml

**47.**	47582	Type 1 diabetes mellitus with renal complications
**48.**	47649	Type 1 diabetes mellitus with ophthalmic complications
**49.**	47650	Type 1 diabetes mellitus with multiple complications
**50.**	49146	Type I diabetes mellitus with neurological complications
**51.**	49276	Insulin-dependent diabetes mellitus with ophthalmic comps
**52.**	49554	Type 1 diabetes mellitus with diabetic cataract
**53.**	49949	Unstable type I diabetes mellitus
**54.**	50960	Pre-existing diabetes mellitus, insulin-dependent
**55.**	51261	Insulin dependent diabetes mellitus
**56.**	51957	Type I diabetes mellitus with ulcer
**57.**	52104	Insulin dependent diabetes mellitus with multiple complicatn
**58.**	52283	Insulin-dependent diabetes mellitus with neurological comps
**59.**	53200	Diabetes mellitus, juvenile type, with ketoacidosis
**60.**	54008	Type 1 diabetes mellitus with neuropathic arthropathy
**61.**	54600	Unstable insulin dependent diabetes mellitus
**62.**	55239	Type 1 diabetes mellitus with gastroparesis
**63.**	56448	Insulin-dependent diabetes without complication
**64.**	57621	Insulin dependent diabetes mellitus with nephropathy
**65.**	60107	Unstable type I diabetes mellitus
**66.**	60208	Type I diabetes mellitus with neuropathic arthropathy
**67.**	60499	Insulin dependent diabetes mellitus with gangrene
**68.**	61344	Type I diabetes mellitus with renal complications
**69.**	61829	Type 1 diabetes mellitus with neurological complications
**70.**	62209	Type I diabetes mellitus with ketoacidosis
**71.**	62352	Type I diabetes mellitus with arthropathy
**72.**	62613	Type I diabetes mellitus without complication
**73.**	63017	Type I diabetes mellitus maturity onset
**74.**	64446	Insulin dependent diab mell with peripheral angiopathy
**75.**	65616	Insulin dependent diabetes mellitus with arthropathy
**76.**	66145	Type I diabetes mellitus with ketoacidotic coma
**77.**	66872	Type I diabetes mellitus with nephropathy
**78.**	68105	Type 1 diabetes mellitus with mononeuropathy
**79.**	68390	Type 1 diabetes mellitus with ulcer
**80.**	68792	Diabetes mellitus, juvenile type, + unspecified complication
**81.**	69043	Dietary advice for type I diabetes
**82.**	69676	Type 1 diabetes mellitus without complication
**83.**	69748	Diabetes mellitus, juvenile type, + ophthalmic manifestation
**84.**	69993	Type 1 diabetes mellitus with gangrene
**85.**	70766	Type 1 diabetes mellitus with hypoglycaemic coma
**86.**	72702	Insulin dependent diabetes mellitus - poor control
**87.**	85660	Diabetes type 1 review
**88.**	91942	Type I diabetes mellitus with multiple complications
**89.**	91943	Type I diabetes mellitus with polyneuropathy
**90.**	93468	Type 1 diabetes mellitus with peripheral angiopathy
**91.**	93875	Insulin dependent diabetes mellitus with retinopathy
**92.**	93878	Type I diabetes mellitus with ulcer
**93.**	93922	Diabetes mellitus, juvenile type, with renal manifestation
**94.**	95343	Type I diabetes mellitus with retinopathy
**95.**	95992	Type I diabetes mellitus without complication
**96.**	96235	Type I diabetes mellitus maturity onset

For peer review only - http://bmjopen.bmj.com/site/about/guidelines.xhtml

**97.**	97446	Type 1 diabetes mellitus maturity onset
**98.**	97474	Unstable type 1 diabetes mellitus
**99.**	97849	Insulin dependent diabetes maturity onset
**100.**	97894	Type I diabetes mellitus with exudative maculopathy
**101.**	98071	Insulin-dependent diabetes mellitus with ophthalmic comps
**102.**	98392	Maturity onset diabetes in youth type 1
**103.**	98704	Insulin dependent diabetes mellitus with ulcer
**104.**	99231	Type I diabetes mellitus with mononeuropathy
**105.**	99311	Type I diabetes mellitus with ophthalmic complications
**106.**	99716	Insulin dependent diabetes mellitus with hypoglycaemic coma
**107.**	99719	Insulin-dependent diabetes without complication
**108.**	100770	Insulin dependent diabetes mellitus with diabetic cataract
**109.**	101311	Insulin dependent diabetes mellitus with polyneuropathy
**110.**	101735	Insulin-dependent diabetes mellitus with neurological comps
**111.**	102112	Type I diabetes mellitus with gangrene
**112.**	102163	Insulin dependent diabetes mellitus with nephropathy
**113.**	102620	Type I diabetes mellitus with persistent microalbuminuria
**114.**	102704	Type I diabetic dietary review
**115.**	102740	Type 1 diabetes mellitus with ophthalmic complications
**116.**	102946	Insulin-dependent diabetes mellitus with renal complications
**117.**	104453	Type 1 diabetic dietary review
**118.**	105337	Type I diabetes mellitus - poor control
**119.**	108007	Type I diabetes mellitus with multiple complications
**120.**	108360	Type I diabetes mellitus in remission
**121.**	108724	Type I diabetes mellitus with gastroparesis
**122.**	109051	Insulin dependent diabetes mellitus with gangrene
**123.**	109628	Type 1 diabetes mellitus in remission
**124.**	109837	Type I diabetes mellitus with renal complications
**125.**	109878	Diet advice for insulin-dependent diabetes
**126.**	110400	Type 1 diabetes mellitus with diabetic cataract
**127.**	111106	Type 1 diabetes mellitus without complication

For peer review only

Supplementary material to paper "**Using electronic health records to quantify and stratify the severity of type 2 diabetes in primary care in England: rationale and cohort study design" Zghebi et al. 2017**

Figure S1 Severity hierarchy of cardiovascular disease domains (cardiac muscle- myocardium and pericardium).

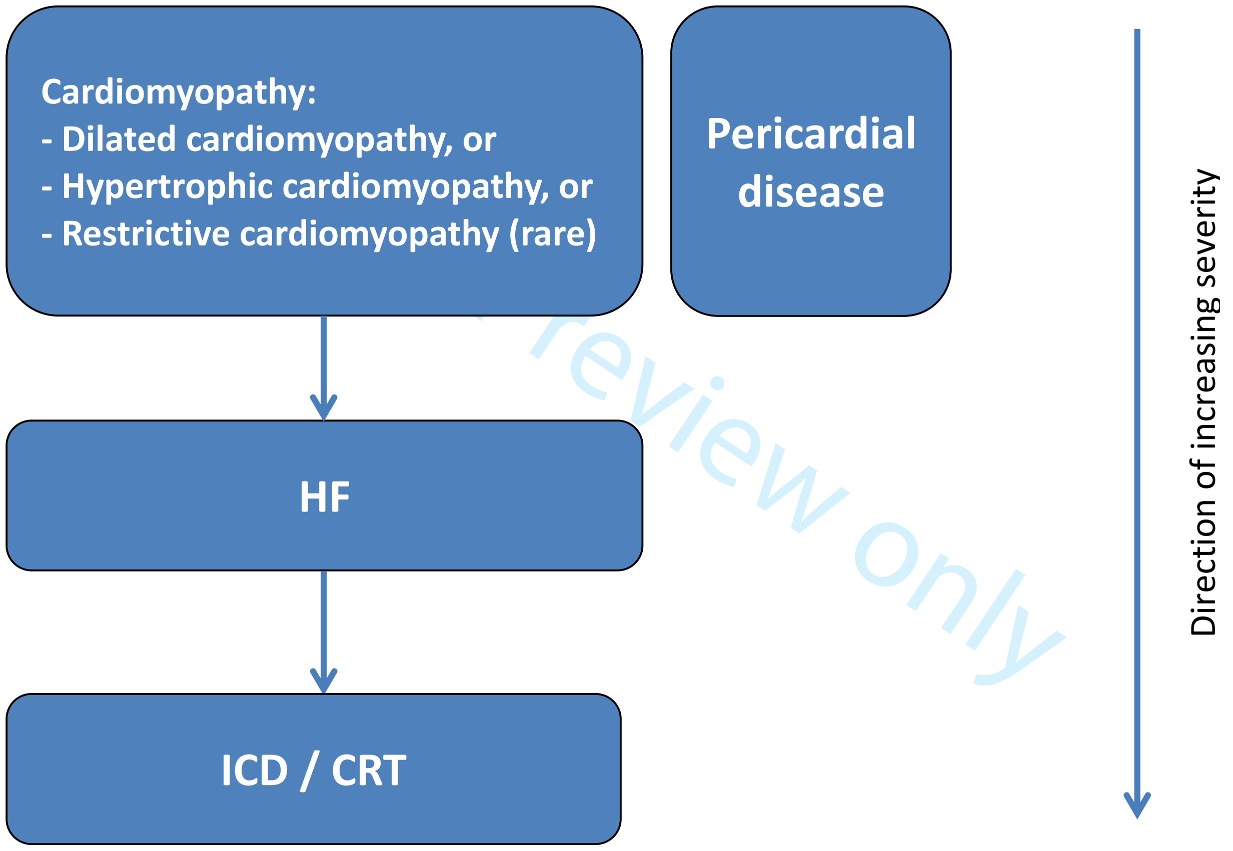

For peer review only - http://bmjopen.bmj.com/site/about/guidelines.xhtml

Supplementary material to paper "**Using electronic health records to quantify and stratify the severity of type 2 diabetes in primary care in England: rationale and cohort study design" Zghebi et al. 2017**

Figure S2 Severity hierarchy of cardiovascular disease domains (Heart valve diseases)

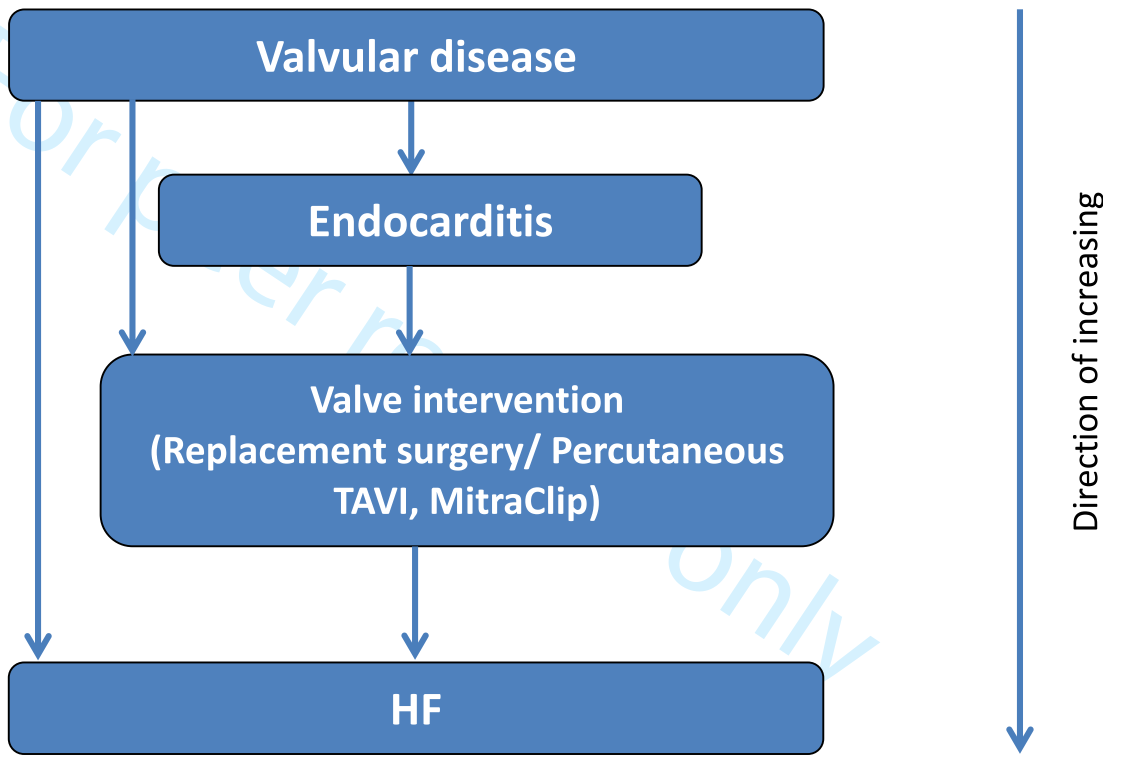

For peer review only - http://bmjopen.bmj.com/site/about/guidelines.xhtml

Supplementary material to paper "**Using electronic health records to quantify and stratify the severity of type 2 diabetes in primary care in England: rationale and cohort study design" Zghebi et al. 2017**

Table S3 Factors used in previous studies to define severity of diabetes

Study ID	Factors used to define diabetes severity / Outcome(s)	Severity scale/ levels	Validation study
**Gini et al. (2016)[(1)]**	• Indication for insulin therapy<br>• The presence of diabetes-related complications<br><br>**Outcomes:**<br>Assessing the positive predictive value (PPV) for the presence/absence of type 2 diabetes against the GP's response to the questionnaire and measure the agreement on the severity level.	Four levels of T2DM severity:<br>• **Level 1**: Clinical definition of the disease, no indication for Insulin therapy and no complications listed in Level 3.<br>• **Level 2**: as in Level 1 except insulin is indicated.<br>• **Level 3**: as in Level 1 except with one of the following complications: (1) arterial stenosis, angina, MI, TIA, ischaemic stroke, intermittent claudication, diabetic foot ulcer, lower limb amputation, (2) retinopathy, (3) incipient or overt diabetic nephropathy/ dialysis.<br>• **Level 4**: As in Level 3 except insulin is indicated	A random sample of identified cases was validated by interviewing their self-selected general practitioners (GPs).
**Grootenhuis et al. (1994)[(2)]**	Type 2 diabetes symptom severity items categorised over six clinical dimensions:<br>• Hyperglycaemic<br>• Hypoglycaemic<br>• Psychological<br>• Cardiovascular<br>• Neuropathic<br>• Ophthalmological<br><br>**Outcomes:**<br>To measure differences in symptom severity between patients and detect changes over time within patients.	A 34-item patient-derived checklist used as a measure for type 2 diabetes symptom severity.	The face and content validity of severity items and dimension structures were based on literature and experiences of diabetologists.
**Young et al. (2008)[(3)]**	From laboratory data and diabetes-related complications:<br>• Retinopathy<br>• Nephropathy<br>• Neuropathy<br>• Cerebrovascular<br>• Cardiovascular<br>• Peripheral vascular disease<br>• Metabolic<br><br>**Outcomes:**<br>Risk of mortality, hospitalisation and healthcare utilisation.	Developed the Diabetes Complication Severity Index (DCSI) that is categorised to two or three levels (0=no abnormality; 1=some abnormality, 2=severe abnormality) depending on the severity of the complication. So if no abnormalities were present, the patient received no score, a 1 was added to the DCSI if patient had any complication classified as some abnormality and 2 was added if patient had any complication classified as severe abnormality.	The study design was a validation sample

For peer review only - http://bmjopen.bmj.com/site/about/guidelines.xhtml

Supplementary material to paper "**Using electronic health records to quantify and stratify the severity of type 2 diabetes in primary care in England: rationale and cohort study design**" **Zghebi et al. 2017**

References

1. Gini R, Schuemie MJ, Mazzaglia G, Lapi F, Francesconi P, Pasqua A, et al. Automatic identification of type 2 diabetes, hypertension, ischaemic heart disease, heart failure and their levels of severity from Italian General Practitioners' electronic medical records: a validation study. BMJ open. 2016;6(12):e012413. Epub 2016/12/13.
2. Grootenhuis PA, Snoek FJ, Heine RJ, Bouter LM. Development of a type 2 diabetes symptom checklist: a measure of symptom severity. Diabetic medicine : a journal of the British Diabetic Association. 1994;11(3):253-61. Epub 1994/04/01.
3. Young BA, Lin E, Von Korff M, Simon G, Ciechanowski P, Ludman EJ, et al. Diabetes complications severity index and risk of mortality, hospitalization, and healthcare utilization. Am J Manag Care. 2008;14(1):15-24.

For peer review only

For peer review only - http://bmjopen.bmj.com/site/about/guidelines.xhtml

Using electronic health records to quantify and stratify the severity of type 2 diabetes in primary care in England: rationale and cohort study design

Journal:	*BMJ Open*
Manuscript ID	bmjopen-2017-020926.R3
Article Type:	Protocol
Date Submitted by the Author:	10-Apr-2018
Complete List of Authors:	Zghebi, Salwa; University of Manchester, NIHR School for Primary Care Research, Centre for Primary Care<br>Rutter, Martin; University of Manchester Faculty of Biology, Medicine and Health, Division of Diabetes, Endocrinology and Gastroenterology; Manchester University NHS Foundation Trust, Manchester Diabetes Centre<br>Ashcroft, Darren; University of Manchester, Centre for Pharmacoepidemiology and Drug Safety,<br>Salisbury, Chris; University of Bristol, Academic Unit of Primary Health Care<br>Mallen, Christian; Keele University, Arthritis Research UK Primary Care Centre<br>Chew-Graham, Carolyn; University of Keele<br>Reeves, David; University of Manchester, Institute of Population Health; Centre for Biostatistics<br>van Marwijk, Harm; University of Brighton<br>Qureshi, Nadeem; University of Nottingham, School of Medicine<br>Weng, Stephen; University of Nottingham, NIHR School of Primary Care Research<br>Peek, N; Health e-Research Centre, Institute of Population Health, University of Manchester<br>Planner, Claire; NIHR School for Primary Care Research, Centre for Primary Care, Institute of Population Health<br>Nowakowska, Magdalena ; University of Manchester, NIHR School for Primary Care Research, Centre for Primary Care<br>Mamas, Mamas; Keele Cardiovascular Research group, Centre for Prognosis Research, Institute for Primary Care and Health Sciences, Keele University<br>Kontopantelis, Evangelos; University of Manchester, Faculty of Biology, Medicine and Health; NIHR School for Primary Care Research, Centre for Primary Care
**Primary Subject Heading**:	Diabetes and endocrinology
Secondary Subject Heading:	General practice / Family practice, Health services research
Keywords:	Type 2 diabetes, Electronic Health Records, PRIMARY CARE, Hospitalisation records, Cardiovascular disease, Diabetes Severity Algorithm

For peer review only - http://bmjopen.bmj.com/site/about/guidelines.xhtml

SCHOLARONE™
Manuscripts

For peer review only

Using electronic health records to quantify and stratify the severity of type 2 diabetes in primary care in England: rationale and cohort study design

Salwa S Zghebi,[1,2] Martin K Rutter,[3,4] Darren M Ashcroft,[5] Chris Salisbury,[6] Christian Mallen,[7] Carolyn A. Chew-Graham,[7] David Reeves,[1] Harm Van marwijk,[8] Nadeem Qureshi,[9] Stephen Weng,[9] Niels Peek,[10] Claire Planner,[1] Magdalena Nowakowska,[1,2] Mamas Mamas,[11] Evangelos Kontopantelis[1,2]

1. Division of Population Health, Health Services Research and Primary Care, School of Health Sciences, Faculty of Biology, Medicine and Health, Health Innovation Manchester, University of Manchester, Manchester, UK.

2. NIHR School for Primary Care Research, University of Manchester, Manchester, UK.

3. Division of Diabetes, Endocrinology and Gastroenterology, School of Medical Sciences, Faculty of Biology, Medicine and Health, University of Manchester, Manchester, M13 9PL, UK.

4. Manchester Diabetes Centre, 193 Hathersage Rd, Manchester University NHS Foundation Trust, Manchester Academic Health Science Centre, Manchester, M13 0JE, UK.

5. Division of Pharmacy and Optometry, School of Health Sciences, Faculty of Biology, Medicine and Health, Health Innovation Manchester, University of Manchester, Manchester, UK.

6. Centre for Academic Primary Care, Population Health Sciences, Bristol Medical School, University of Bristol, Bristol, UK.

7. Research Institute for Primary Care and Health Sciences, Keele University, Staffordshire, UK.

8. Division of Primary Care and Public Health, Brighton and Sussex Medical School, University of Brighton, Brighton, UK.

9. School of Medicine, University of Nottingham, Nottingham, UK.

10. Division of Informatics, Imaging & Data Sciences (L5), School of Health Sciences, Faculty of Biology, Medicine and Health, Health Innovation Manchester, University of Manchester, Manchester, UK.

11. Keele Cardiovascular Research group, Centre for Prognosis Research, Institute for Primary Care and Health Sciences, Keele University, Stoke-on-Trent, UK.

Corresponding author. Salwa S Zghebi. Division of Population Health, Health Services Research and Primary Care, University of Manchester, Manchester, UK. Email address: Salwa.Zghebi@manchester.ac.uk

Number of words (Abstract): 300

For peer review only

For peer review only - http://bmjopen.bmj.com/site/about/guidelines.xhtml

Number of words (full text): 3,996
Number of Tables 1
Number of Figures: 4

For peer review only

For peer review only - http://bmjopen.bmj.com/site/about/guidelines.xhtml

Abstract

Introduction: The increasing prevalence of type 2 diabetes (T2DM) presents a significant burden on affected individuals and health-care systems internationally. There is, however, no agreed validated measure to infer diabetes severity from electronic health records (EHRs). We aim to quantify T2DM severity and validate it using clinical adverse outcomes.

Methods and Analysis: Primary care data from the Clinical Practice Research Datalink (CPRD), linked hospitalisation and mortality records between April-2007 and March-2017 for T2DM patients in England will be used to develop a clinical algorithm to grade T2DM severity. The EHR-based algorithm will incorporate main risk factors (severity domains) for adverse outcomes to stratify T2DM cohorts by baseline and longitudinal severity scores. Provisionally, T2DM severity domains, identified through a systematic review and expert opinion are: diabetes duration, HbA_{1c}, microvascular complications, comorbidities, and co-prescribed treatments. Severity scores will be developed by two approaches: i) calculating a count score of severity domains; ii) through hierarchical stratification of complications. Regression models estimates will be used to calculate domains weights. Survival analysis for the association between weighted severity scores and future outcomes: cardiovascular events; hospitalisation (diabetes-related, cardiovascular); and mortality (diabetes-related, cardiovascular, all-cause mortality) will be performed as a statistical validation. The proposed EHR-based approach will quantify the T2DM severity for primary care performance management and inform the methodology for measuring severity of other primary care-managed chronic conditions. We anticipate that the developed algorithm will be a practical tool for practitioners, aid clinical management decision-making, inform stratified medicine, support future clinical trials and contribute to more effective service-planning and policy-making.

Ethics and Dissemination: The study protocol was approved by the Independent Scientific Advisory Committee (ISAC). Some data were presented at the NIHR SPCR Showcase, September-2017, Oxford,UK; the Diabetes UK Professional Conference March-2018, London,UK. The study findings will be disseminated in relevant academic conferences and peer-reviewed journals.

For peer review only - http://bmjopen.bmj.com/site/about/guidelines.xhtml

Strengths and limitations of this study

- This is the first UK-based study to develop a diabetes severity scoring tool based on real-world electronic healthcare data to grade people with type 2 diabetes by their clinical severity.
- The study will include a large sample size and use high-quality medical data routinely-collected from general practices with access to linked national hospitalisation and cause-specific mortality datasets.
- The association between the computed severity scores and future adverse outcomes (cardiovascular event, hospitalisation and mortality) will be used to validate the developed severity algorithm.
- There is a possibility to miss other severity indicators not recorded in the data sources used such as pharmacy dispensing data.
- Given that the linkage scheme is only available for consented general practices in England, the study cohort will be restricted to eligible patients registered within England (form nearly 58% of total CPRD general practices).

For peer review only

Introduction

The worldwide prevalence of diabetes mellitus is increasing with World Health Organisation (WHO) estimating that over 422 million adults had diabetes in 2016 with a growth in the global prevalence from 4.7% in 1980 to 8.5% in 2014.[1,2] In the UK, the prevalence of type 2 diabetes (T2DM) has nearly doubled between 2004 and 2014.[3,4] People with diabetes have a higher risk for morbidity and mortality when compared to individuals without diabetes,[3,5] and hence the increasing prevalence presents a significant burden on health care resources. Diabetes management expenses are estimated to consume up to 11% of the total health care budget in the UK and USA.[6,7] Diabetes is a complex metabolic condition, of an increasing severity,[8,9] at individually varying levels, and progressive development of vascular complications and end organ damage over time. Diabetes is mainly managed in primary care in the UK,[10] overall Europe,[11] USA,[12] and Asia.[13,14] In the UK, nearly 75% of the diabetes-associated costs relate to management of diabetic complications.[6]

The 'severity' of clinical conditions can be conceptualised as a progression of the underlying disease process. Increasing disease severity and development of associated complications lead to greater treatment complexity and clinical impact. The severity of chronic conditions, such as diabetes, has not been widely considered despite the clinical relevance and its likely impact on health care systems, where few studies have assessed disease severity among patients with T2DM or have quantified temporal trends or scores of severity.[15,16]

Severity scores could be clinically useful, particularly in T2DM, as such a new summary score could contribute above what existing risk scores offer (e.g. moving beyond risk for death) and also be highly relevant for use in six main clinical and research areas: i) identifying complex patients with a higher need for future care; ii) identifying patients at early stages of disease (benchmarking); iii) providing information that will directly inform clinical care; iv) identifying trajectories in severity over time; v) supporting research, for example, by serving as an important diabetes-specific covariate to consider in analyses, similarly to the Charlson Comorbidity Index, or as an outcome; and vi) providing data that will inform resource allocation in health systems, such as the National Health Service (NHS).

For peer review only - http://bmjopen.bmj.com/site/about/guidelines.xhtml

Here, we describe the conceptual development of a T2DM severity algorithm model, using electronic health records (EHRs) on clinical consultations and treatments. Our aim is to use routinely-collected clinical and administrative data to develop a scoring tool which would quantify and grade the severity of T2DM. This model could potentially be used by clinicians to stratify patients based on disease severity and we aim to demonstrate potential advantages over similar risk scores. To validate the algorithm, our secondary aim is to examine the association of severity grades with risk of three main adverse outcomes: cardiovascular disease, hospitalisation, and mortality. The developed severity stratification algorithm is anticipated to have direct impact on clinical practice and have wider implications on service-planning and policy-making.

Methods and Analysis

Data Source

This study will use data from the Clinical Practice Research Datalink (CPRD). The CPRD is one of the world's largest EHR with anonymised primary care records from over 650 general practices over the UK.[17] Nearly 76% of all CPRD registered patients are in England.[17] CPRD data include clinical diagnoses, prescribed therapies, biochemical test results and referrals to healthcare services. The accuracy and completeness of diagnostic coding and validity of CPRD data, in clinical research, have been reported as excellent with positive predictive values (PPVs) of over 90% for nearly 14 conditions (including diabetes and cerebrovascular disease).[18] PPVs were defined as the proportion of CPRD diagnoses that were validated as true cases when compared to a gold standard such as GP questionnaire or primary care records.

Two metrics for research-quality data, recommended to be used by researchers, are available for CPRD records.[17] For patient-level data, the flag 'acceptable' indicates that a patient's record has met certain quality standards such as registration status, valid age and gender, and record of patient events. For practice-level data, the up-to-standard (UTS) date is used as a data quality measure. The UTS date is calculated for each CPRD general practice

For peer review only - http://bmjopen.bmj.com/site/about/guidelines.xhtml

as the latest date at which the general practice meets minimum quality criteria based on two central concepts: the continuity of recorded data and the number of recorded deaths, in comparison to an expected national range.[17,19]

Nearly 75% of general practices in England (approximately 58% of all CPRD practices) have consented to the CPRD Linkage Scheme for access to a number of linked datasets and national disease registries.[17] These include hospital data (including outpatient, admissions, and Accident and Emergency (A&E) data), mortality records (held by the Office for National Statistics (ONS)), socio-economic status and the cancer registry. The linked hospitalisation records, held by the Hospital Episode Statistics (HES), provide ethnicity data, admission and discharge dates, clinical diagnoses and procedures during hospitalisation recorded using the tenth revision of the International Classification of Diseases (ICD-10) and operating procedure codes (OPCS). This study will only include patients registered with English general practices which provide up-to-standard data and which participate in the CPRD Linkage Scheme.

Study Population

The study cohort will include individuals aged 35 years or over with T2DM (with ≥1 diagnostic code as in Table S1) between 1st April 2007 and 31st March 2017. Patients with an ever record of type 1 diabetes (with ever code for type 1 diabetes as in Table S2) will be excluded unless have records of non-insulin antidiabetic therapies. We chose the study period after 2006 as the quality of CPRD data has improved substantially to adhere with the then introduced important changes to the national incentive scheme The Quality and Outcomes Framework (QOF), an incentivisation programme for all GP surgeries in the UK.[20,21] QOF exception reporting process allows general practices to exclude patients from indicators or a clinical domain based on discretionary exception codes. However, evidence on the use of exception codes has shown that they are being used appropriately by practices and overall exception rates for diabetes patients are low.[22]

For this cohort construction, we will consider implementing previously-validated algorithms designed to identify diabetes cases by avoiding potential misclassification in routinely-

For peer review only - http://bmjopen.bmj.com/site/about/guidelines.xhtml

collected data such as CPRD.[23,24] Eligible patients will be followed-up until censored at the earliest instance of any of the following event dates: patient transferred out of the practice (any cause), last collection date for the practice, the study end on the 31st March 2017 or death. The main demographic and clinical characteristics of the defined diabetes cohorts (such as age, gender, geographic region within England, patient-level and general practice-level social deprivation, BMI, and baseline HbA_{1c}) will be identified. Based on our previous studies, the expected sample size in a year will include 11,000 T2DM patients registered with English general practices linked to the HES Admitted Patient Care dataset, HES Outpatient and HES A&E datasets. A random 80% of the identified diabetes cohort (training dataset) will be used to develop the severity tool, with the remaining 20% of the cohort used as a validation dataset, as described below.

Severity Domains

A systematic literature search for studies that developed algorithms or models to assess and quantify the severity of diabetes was conducted to identify the domains and sub-domains for T2DM severity (to be published separately). Also, expert clinical opinion from members of the research team was used to supplement the search process and identify possible omissions. Clinical members in the team include pharmacists (DMA, SSZ), general practitioners (CS, CM, CCG, HVm, and NQ), a consultant diabetologist (MKR) and a consultant cardiologist (MM), who used their expertise to create a list of relevant clinical domains for T2DM severity. The final domains to be included in the severity model will be decided during the analysis stage. Currently, the identified clinical domains that are relevant to the degree of progression of T2DM include: patient factors (diabetes duration (the period between T2DM diagnosis and the severity score estimation) and body mass index (BMI)), monitoring laboratory tests (glycated haemoglobin (HbA_{1c}) categories (threshold of 7% [53 mmol/mol]), blood glucose levels), type of antidiabetic therapy, other prescribed medications (such as lipid-regulating medications and angiotensin-converting enzyme inhibitors (ACEIs)), comorbidities (including diabetes-related complications, depression), hospitalisation, and surgical interventions. Comorbidities will be identified using appropriate code lists and contribute to the severity score according to each score computing method.

For peer review only - http://bmjopen.bmj.com/site/about/guidelines.xhtml

The identified domains and sub-domains are described in Table 1. Domains will also be reviewed by a panel of 'experts by experience' (people with lived experience of T2DM) to provide patient validation of the severity scoring tool.

The clinical Read codes for the defined severity domains codes and the product codes for drug therapies will be identified using the [pcdsearch] Stata user command.[25] The [pcdsearch] command is a search programme developed to extract code lists from typically very long lookup files associated with primary care databases using an input file containing a list of stubs for codes of interest to be searched for. For CPRD, the lookup files Medical dataset is searched for all clinical Read codes and the Product lookup file, that includes unique product codes, is searched for all treatments. The corresponding ICD-10 codes will be used to identify clinical domains recorded in the hospitalisation data and ONS mortality records. The relevant CPRD operational identification entities for the laboratory tests will be identified. This is an ongoing process whereby the final lists of domains and codes will be reached by consensus among the clinical members of the team. All code lists will be available on the online clinical code repository (ClinicalCodes.org).[26]

Study Outcomes

The adverse outcomes of interest will be the development of the first event among cardiovascular disease, (myocardial infarction, stroke), future hospitalisation (any hospitalisation, diabetes-related and cardiovascular hospitalisation) and death (diabetes-related mortality, cardiovascular mortality and all-cause mortality). Secondary outcome will be future hospitalisation due to hypoglycaemia, a relevant and potentially preventable adverse outcome. The hospitalisation and death outcomes will be identified using the linked HES and ONS mortality data, respectively. Similarly with severity domains, Read codes and ICD-10 codes (available on ClinicalCodes.org online repository) will be used to identify the outcomes as appropriate.

For peer review only - http://bmjopen.bmj.com/site/about/guidelines.xhtml

Statistical Analyses

Diabetes Severity Algorithm

Using annual data bins and grouping diabetes patients in the training dataset (include random 80% of the total diabetes cohort) from 1st April to 31st March between 2007 to 2017, the developed diabetes algorithm will grade the severity of T2DM using pre-defined (sub)domains. Our study period (after 2006) was selected to ensure very high data quality in primary care, while the addition of secondary care data will make our analyses even more robust, in terms of accurately classifying T2DM severity levels.

We will consider two approaches to derive numerical or categorical diabetes severity scores or levels. Firstly, we will use a binary classification (severity indicator: present/absent) within each sub-domain and calculate an aggregate score. The second approach, through hierarchical stratification of end organ microvascular and macrovascular complications, will involve increasing weighting within each sub-domain, as severity increases where scores of 1, 2, or 3 on each subdomain will be assigned based on clinical input in terms of severity. Then, regression model, using death (primary outcome) and future hospitalisation (secondary outcome) as dependent variable, will be fitted from which the weights of its estimates will be used to calculate the weights for severity sub-domains. The highest possible severity score will be known when the final list of included domains and sub-domains is decided. Hierarchical diagrams of the clinical severity of identified diabetes-related complications domains (Figure 1) cerebrovascular (Figure 2), and cardiovascular (Figure 3, Figure 4, Figure S1, Figure S2) domains are presented.

Sub-domains with many measurements over time, such as HbA_{1c} levels and BMI, will be modelled as time-varying covariates (alternative models will be considered to account for non-linear changes). Within each time window the average of these measurements will be used, and we will also use multiple imputation approaches to account for missing data. Provisionally, we will consider using data records within look-back periods between 3-5 years. Different look-back windows within this period will be tested to obtain the optimal time window. Descriptive statistics of the study population and the estimated scores will be performed.

For peer review only - http://bmjopen.bmj.com/site/about/guidelines.xhtml

Severity Algorithm Validation

When we have developed a first version of the severity score tool, it will be validated statistically by quantifying the association of severity scores to future adverse outcomes. The primary outcome will be developing a cardiovascular event (myocardial infarction, stroke), future hospitalisation and death (diabetes-related mortality, cardiovascular mortality and all-cause mortality). A secondary adverse outcome will be future hospital admission due to hypoglycaemia, a relevant and potentially preventable adverse outcome. Cox proportional hazards (after assessing proportional hazards assumption) and/or competing-risks regressions will be used to perform survival analyses and assess the relationship between the severity score and outcomes controlling for all available patient characteristics (such as age, gender, ethnicity, and level of social deprivation). We aim to analyse and assess how severity scores differ across different levels of these variables. As stated earlier, a random 20% of the study cohort (validation dataset) will be used to validate the performance of the severity algorithm that was developed in the training dataset (80% of the diabetes cohort). Using the calculated subdomains weights (derived by the regression model estimates), Cox regression analysis will be used to validate the developed severity algorithm and we will assess the model performance using relevant measures (such as area under the receiver operating characteristic curve (AUROC). Using this analysis, we will also test what how much the 'sophisticated' full model that includes all relevant severity domains will add in terms of predictive validity of the adverse outcomes over a simpler model that includes demographic data (age, gender and level of social deprivation) alone.

Longitudinal Trends of Diabetes Severity

In newly diagnosed T2DM patients during the study period we will assess the temporal trends of severity scores over time by calculating the time needed to progress along the quintiles of the severity scale starting from the date or year of diagnosis and identify the predictors to this progression across quintiles.

For peer review only - http://bmjopen.bmj.com/site/about/guidelines.xhtml
For peer review only

While the inclusion of some domains such as carotid artery stenting, the use of ACEIs or lipid-regulating drugs, kidney transplantation and laser therapy are clinically-relevant and represent markers of greater level of disease severity they also improve the prognosis (lessen the grade of severity) i.e. raising a risk of "feedback". While we will further assess their role, we may have to not include these domains in the modelling at the analysis stage. All statistical analyses will be conducted using Stata Statistical Software for Windows: *Release 15* (College Station, TX: StataCorp LP, USA).

Patient and Public Involvement and Engagement (PPIE)

The public interest in the reuse of routinely-collected electronic health data for research purposes has expanded over recent years. Our study presents a valuable opportunity to address the challenge of how to develop meaningful and productive PPIE collaborations in observational studies that make use of secondary data.

We will identify suitable PPIE partners to collaborate on this study and develop creative strategies for meaningful involvement as the project develops. Participants to provide their input that will be particularly valuable in providing feedback into the diabetes severity algorithms, and deciding what information would be most useful to patients and how to be presented.

In the latter stages of the study, jointly with our PPIE partners, we will plan and deliver a patients' and carers' workshop where we will present emerging findings and seek feedback to inform further work and the dissemination strategy. This will include an exercise around weights of domains within the algorithm. PPIE work will be reported using the GRIPP (Guidance for Reporting Involvement of Patients and Public) checklist.[27]

For peer review only - http://bmjopen.bmj.com/site/about/guidelines.xhtml

Discussion

The global and country-specific prevalence of diabetes has increased substantially.[2,28-31] For T2DM, prevalence rates have doubled over the past two decades.[4,32,33] Diabetes diagnosis is associated with higher risk for morbidity and mortality in comparison to people without diabetes.[5] Despite the natural progression of most chronic diseases, including T2DM, and their important clinical implication on prognosis and medical resources utilisation, only a few studies have attempted to grade the severity of chronic conditions over time. Our proposed study aims to develop a contemporary algorithm to quantify the severity of T2DM, a highly prevalent condition, using routine EHR. The scoring tool will be based on clinically-relevant diagnoses and treatments modelled as severity domains and sub-domains. The algorithm will enable clinicians to grade T2DM patients according to the level of their disease severity, at baseline and longitudinally, as driven by the weights of the included clinical domains.

Prior Studies

Currently, there is limited data around validated severity measures that can be used in routine clinical practice managing patients with T2DM. Consequently, we are in parallel finalising a conducted systematic review for clinical-based diabetes severity models which will inform the refinement of this work. Here, we discuss three relevant diabetes severity measures that have been previously reported from countries outside the UK.

In the first study, Gini et al. (2016) categorised the severity of T2DM (N=300) into four levels based on insulin use and the presence of diabetic complications (Table S3).[15] A validation study in a random sample of cases was performed by interviewing their general practitioners. We however, aim to include more T2DM patients and consider more clinical data such as other coexisting conditions, non-insulin therapies, statins and ACEIs, that have been shown to be associated with reduced risk of adverse outcomes.

In the second study, a diabetes symptom checklist was created to measure the perceived symptom severity and assess the changes over time in 185 T2DM patients.[16] The checklist

For peer review only - http://bmjopen.bmj.com/site/about/guidelines.xhtml

contained 34 items categorised by main clinical symptoms (Table S3). The patterns of comorbidities and prescribed treatments were reportedly associated with significant differences in the estimated severity scores.[16] The sample size was however relatively small and the checklist was based on patients' perception on diabetes symptoms severity.

Thirdly, Young et al. (2008), calculated a diabetes complications severity index (DCSI) in 4,229 patients with type 1 or type 2 diabetes in one US geographic region to examine its association with adverse outcomes (risk of hospitalisation and mortality). In comparison to using a simple numerical count of complications, DCSI found to be a better tool to predict adverse outcomes.[34] The authors used pharmacy (insulin use only) and laboratory data to compute the severity index and included patients from clinics with largest ethnic diversity. However, the DCSI missed additional domains such as diabetes duration, hyperlipidaemia and a wider range of diabetes-related and end-organ damage manifestations that we aim to assess in addition to hospitalisation and mortality.

In the wider literature, although several studies have investigated the possible role of other various factors on the severity of diabetes, none have provided a severity scoring tool that utilises data from various clinical domains as planned in our study that can use the wealth of information routinely-collected in electronic healthcare databases. In these studies, approaches used to define diabetes severity included: comparing T2DM severity, before and after obesity surgery;[35] examining the association between diabetes severity and either: haematological and immunological changes,[36] levels of urine citrate,[37] a biomarker for adverse outcomes;[38] grip strength;[39] or the use of complementary medicine to manage T2DM.[40]

Overall, the severity of T2DM has been assessed using the following domains: the complexity of antidiabetic treatment regimens[35] or HbA_{1c} levels;[37] a summary severity variable that includes vascular complications;[41] or a health status composite, number of comorbidities and patterns of treatments.[42,43] Other putative and less clinically-robust indicators or animal models were reportedly used to assess the severity of T2DM such as the effect of different patient education approaches on diabetes severity;[44] evaluating the effect of parental history;[45] and the role of genetic,[46,47] metabolic,[48] or inflammatory mediators.[49]

For peer review only - http://bmjopen.bmj.com/site/about/guidelines.xhtml

In comparison to our planned study, none of previous studies included as many routinely-collected clinically-relevant variables as in our more inclusive summary severity score algorithm or assessed the association of estimated scores with the various adverse outcomes included in our defined primary and secondary endpoints.

Potential Strengths and Limitations

Our proposed study has several potential strengths: First, it aims to present a contemporary measure to the available tools and the first UK-based study to develop an EHR-based severity scoring tool to grade patients by their T2DM severity. Second, the study uses high-quality real-world medical data routinely-collected from general practices. The use of routinely-collected data indicates that severity scores can be generated automatically with minimal effort. Third, the views of PPIE collaborators will be incorporated in the development of the severity tool. Fourth, we will access two linked datasets: hospitalisation (HES) data (to maximise data capture and reduce condition misclassification), and cause-specific mortality data to ascertain causes of death. Fifth, the sample size is expected to be large enough to drive the development and evaluation of the severity algorithm. Sixth, a statistical validation of the developed algorithm is planned and described.

As we will use data available in CPRD and HES datasets, one of the limitations we anticipate is the possibility to miss other severity indicators not recorded in used datasets. These include detailed pharmacy data such as 'actual' dispensing and adherence data. Also, the use of routinely-collected data is associated with missing values, being collected from questionnaires, and issues around the accuracy of coding. However, we plan to use appropriate imputation methods and definite criteria to minimise the effect of coding issues. A possible limitation that should be acknowledged is underestimated and poorly represented T2DM severity levels for patients not regularly attending a general practice, people missing appointments or not being reliably captured in the database due to very high mobility status (e.g. homelessness). This limitation aligns with QOF exception reporting that allows practices to exclude patients from indicators or a clinical domain based on discretionary exception codes. However, the use of QOF exception coding was considered

For peer review only - http://bmjopen.bmj.com/site/about/guidelines.xhtml

appropriate and its levels were very low, especially for informed dissent.[22] Another limitation is that our study will be restricted to patients registered with general practices in England, as CPRD currently only provides linkage to external national datasets for consented general practices in England. However, English general practices form the majority of all CPRD practices including nearly 76% of total registered patients.[17] Finally, due to project time constrains, there is no possibility to validate the developed diabetes scores using questionnaires (as reported previously *[15]*) or replicating the algorithm in a separate dataset. However, we have planned a statistical validation of the developed algorithm.

Importance and the Clinical Implications of the Severity Tool

The developed algorithm and severity tool may have significant implications for primary care both in terms of disease management and resource allocation. Ideally, through future work of further validation and assessment of clinical utility, the severity tool will be of practical use in primary care through its implementation in the clinical computing systems used in the UK.[50] The clinical significance of the developed severity algorithm to primary care is driven by the inclusion of highly-relevant clinical domains, such as diabetes-related complications and comorbidities, mapped to routinely-collected data.

Additionally, by assessing the longitudinal patterns of severity, the developed tool may be more clinically relevant than the currently used proxy (HbA_{1c}), and thus it could be a more reliable indicator in informing practices' remuneration for diabetes care. Categorising individuals based on their diabetes severity will be relevant for risk stratification (which may enable safer delegation of care within the clinical team), identify individualised patient risks and will help practitioners triage patients in need of a greater clinical input which informs towards stratified medicine to reduce future life-changing diabetes-related complications. Moreover, the weights of the severity scores may inform future clinical trials as the scoring tool considers a broader range of cardiovascular conditions than in most randomised clinical trials. Given the relatively low rates of cardiovascular outcomes in some trials, identifying diabetes patients who are at higher risk via our severity algorithm would help to power trials with overall longer term benefit for patients. Finally, the composite severity score may

For peer review only - http://bmjopen.bmj.com/site/about/guidelines.xhtml

For peer review only

serve as an important confounding factor in future research, as for example, to match diabetes cases and controls in observational studies and clinical trials.

Ethics and Dissemination

The study protocol was approved by the CPRD's Independent Scientific Advisory Committee (ISAC). Some data were presented at the annual National Institute for Health Research School for Primary Care Research (NIHR SPCR) Showcase, September 2017, Oxford, UK and at the Diabetes UK Professional Conference, March 2018, London, UK. The study findings will be disseminated in relevant academic conferences and peer-reviewed journals.

Author Contributions

E.K. S.S.Z. M.M., M.K.R and H.V.m developed the study design and data analysis plan. S.S.Z. M.M., M.K.R and H.V.m agreed on provisional clinical code lists. S.S.Z. prepared the first draft of the manuscript, and E.K., M.M., M.K.R. and H.V.m critically reviewed initial versions. C.P. contributed to the planned PPIE work. D.R., C.C.G., C.M., N.P., D.M.A., C.S., N.Q., M.N. and S.W reviewed and critically edited the manuscript. All authors approved the final version of the protocol before submission. S.S.Z. is the guarantor.

Funding

This study is funded by the National Institute for Health Research School for Primary Care Research (NIHR SPCR), grant number 331. This report is independent research by the National Institute for Health Research. The views expressed in this publication are those of the authors and necessarily those of the NHS, the National Institute for Health Research or the Department of Health.

Competing Interests

E.K., H.V.m, M.M., D.R., C.C.G. C.P., C.M., N.P., M.N. declare no competing interests. S.S.Z. reports support by the NIHR SPCR during this study. D.M.A. has received grant funding from Abbvie and has served on advisory boards for Pfizer and GSK. M.K.R. has received

For peer review only - http://bmjopen.bmj.com/site/about/guidelines.xhtml
For peer review only

educational grant support from MSD and Novo Nordisk; has modest stock ownership in GSK; and has consulted for Roche. N.Q. reports grants from the NIHR SPCR, during the conduct of the study. C.S. reports grants from NIHR SPCR, during the conduct of the study; grants from NHS CLAHRC West, grants from Avon Primary Care Research Collaborative, outside the submitted work. S.W. serves as a member of the Clinical Practice Research Datalink Independent Scientific Committee (ISAC) at the UK Medicines and Health Regulatory Agency.

List of Abbreviations

ACEI: Angiotensin converting enzyme inhibitors; **CABG**: coronary artery bypass graft; **CHD**: coronary heart disease; **CKD**: chronic kidney disease; **CPRD**: Clinical Practice Research Datalink; **CRT**: cardiac resynchronization therapy; **DCSI**: diabetes complications severity index; **EHR**: electronic health record; **EVAR**: Endovascular aneurysm repair; **GRIPP**: Guidance for Reporting Involvement of Patients and Public; **HbA_{1c}**: glycated haemoglobin; **HES**: Hospital Episode Statistics; **HES A&E:** HES Accident & Emergency; **HF**: heart failure; **ICD**: implantable cardioverter defibrillator; **ICD-10**: the 10th revision of the International Classification of Diseases; **ISAC**: Independent Scientific Advisory Committee; **MI**: myocardial infarction; **NHS:** National Health Service; **ONS**: Office for National Statistics; **OPCS**: operating procedure codes; **PAD**: peripheral artery disease; **PCI**: percutaneous coronary intervention; **PPIE:** patient and public involvement and engagement; **PTCA**: percutaneous transluminal coronary angioplasty; **PVD**: peripheral vascular disease; **QOF**: Quality and Outcomes Framework; **SVT**: supraventricular tachycardia; **TAVI**: transcatheter aortic valve implantation; **T2DM**: type 2 diabetes; **TIA**: transient ischemic attack; **VF**: ventricular fibrillation; **VT**: ventricular tachycardia; **WHO**: World Health Organization.

For peer review only

References

1. World Health Organization (WHO) media centre. Diabetes. [Fact sheet] 2016 [cited 2016 29 April]; Available from: http://www.who.int/mediacentre/factsheets/fs312/en/.
2. World Health Organization (WHO). Global Report on Diabetes. 2016 [cited 2017 11 February]; Available from: http://apps.who.int/iris/bitstream/10665/204871/1/9789241565257_eng.pdf.
3. Zghebi SS, Steinke DT, Carr MJ, Rutter MK, Emsley RA, Ashcroft DM. Examining Trends in Type 2 Diabetes Incidence, Prevalence and Mortality in the UK between 2004 and 2014. Diabetes, Obesity and Metabolism. 2017.
4. Sharma M, Nazareth I, Petersen I. Trends in incidence, prevalence and prescribing in type 2 diabetes mellitus between 2000 and 2013 in primary care: a retrospective cohort study. BMJ Open. 2016;6:e010210.
5. American Diabetes Association (ADA). Standards of Medical Care in Diabetes - 2015. Diabetes Care. 2015;38(Suppl 1):S1-S49.
6. Hex N, Bartlett C, Wright D, Taylor M, Varley D. Estimating the current and future costs of Type 1 and Type 2 diabetes in the UK, including direct health costs and indirect societal and productivity costs. Diabetic Medicine. 2012;29:855-62.
7. Walker BR, Colledge NR, Ralston SH, Penman ID. Davidson's Principles and Practice of Medicine. China: Elsevier 2014 [cited 2016 30 June]. Available from: http://www.elsevier-etextbooks.com/pdfreader/davidsons-principles-practice-medicine57814.
8. Walker BR, Colledge NR, Ralston SH, Penman ID. Davidson's Principles and Practice of Medicine: Elsevier; 2014 [cited 2017 11 May]. Available from: http://elsevierelibrary.co.uk/pdfreader/davidsons-principles-practice-medicine57814.
9. Nefs G, Pop VJM, Denollet J, Pouwer F. The Longitudinal Association between Depressive Symptoms and Initiation of Insulin Therapy in People with Type 2 Diabetes in Primary Care. Plos One. 2013;8(11).
10. Murrells T, Ball J, Cookson G, Maben J, Lee G, Griffiths P. Managing diabetes in primary care: how does the configuration of the workforce affect quality of care? National Nursing Research Unit, King's College London, 2013 November 2013. Report No.: Department of Health Policy Research Programme, ref. 016/0058.
11. Luk VE, Patricia S, Luc F, Liesbeth B, Jan DM. Priorities for diabetes primary care in Europe. Primary Care Diabetes. 2008;2(1):3-8.
12. Spann SJ, Nutting PA, Galliher JM, Peterson KA, Pavlik VN, Dickinson LM, et al. Management of Type 2 Diabetes in the Primary Care Setting: A Practice-Based Research Network Study. Ann Fam Med. 2006;4(1):23–31.
13. Tai T-Y, Chuang L-M, Tsai S-T, Huang B-Y, The Diabcare (Taiwan) Study Group. Treatment of Type 2 Diabetes Mellitus in a Primary Care Setting in Taiwan: Comparison with Secondary/Tertiary Care. Journal of the Formosan Medical Association = Taiwan yi zhi. 2006;105(2):105–17.
14. Mafauzy M. Diabetes control and complications in private primary healthcare in Malaysia. Med J Malaysia. 2005;60(2):212-7.
15. Gini R, Schuemie MJ, Mazzaglia G, Lapi F, Francesconi P, Pasqua A, et al. Automatic identification of type 2 diabetes, hypertension, ischaemic heart disease, heart failure and their levels of severity from Italian General Practitioners' electronic medical records: a validation study. BMJ open. 2016;6(12):e012413. Epub 2016/12/13.
16. Grootenhuis PA, Snoek FJ, Heine RJ, Bouter LM. Development of a type 2 diabetes symptom checklist: a measure of symptom severity. Diabetic medicine : a journal of the British Diabetic Association. 1994;11(3):253-61. Epub 1994/04/01.
17. Herrett E, Gallagher AM, Bhaskaran K, Forbes H, Mathur R, van Staa TP, et al. Data Resource Profile: Clinical Practice Research Datalink (CPRD). International Journal of Epidemiology. 2015;44(3):827-36.

For peer review only - http://bmjopen.bmj.com/site/about/guidelines.xhtml

18. Khan NF, Harrison SE, Rose PW. Validity of diagnostic coding within the General Practice Research Database: a systematic review. British Journal of General Practice. 2010;60(572):e128-e36.
19. Williams T, van Staa T, Puri S, Eaton S. Recent advances in the utility and use of the General Practice Research Database as an example of a UK Primary Care Data resource. Therapeutic Advances in Drug Safety. 2012;3(2):89-99.
20. Lester H, Campbell S. Developing Quality and Outcomes Framework (QOF) indicators and the concept of 'QOFability'. Quality in Primary Care. 2010;18:103-9.
21. Calvert M, Shankar A, McManus RJ, Lester H, Freemantle N. Effect of the quality and outcomes framework on diabetes care in the United Kingdom: retrospective cohort study. BMJ. 2009;338:b1870.
22. Doran T, Kontopantelis E, Fullwood C, Lester H, Valderas JM, Campbell S. Exempting dissenting patients from pay for performance schemes: retrospective analysis of exception reporting in the UK Quality and Outcomes Framework. BMJ. 2012;344:e2405.
23. de Lusignan S, Khunti K, Belsey J, Hattersley A, van Vlymen J, Gallagher H, et al. A method of identifying and correcting miscoding, misclassification and misdiagnosis in diabetes: a pilot and validation study of routinely collected data. Diabetic Medicine. 2010;27(2):203-9.
24. Wright AK, Kontopantelis E, Emsley R, Buchan I, Sattar N, Rutter MK, et al. Life Expectancy and Cause-Specific Mortality in Type 2 Diabetes: A Population-Based Cohort Study Quantifying Relationships in Ethnic Subgroups. Diabetes Care. 2017;40(3):338-45.
25. Olier I, Springate DA, Ashcroft DM, Doran T, Reeves D, Planner C, et al. Modelling Conditions and Health Care Processes in Electronic Health Records: An Application to Severe Mental Illness with the Clinical Practice Research Datalink. Plos One. 2016;11(2):e0146715.
26. Springate DA, Kontopantelis E, Ashcroft DM, Olier I, Parisi R, Chamapiwa E, et al. ClinicalCodes: An Online Clinical Codes Repository to Improve the Validity and Reproducibility of Research Using Electronic Medical Records. PLoS ONE. 2014;9(6):e99825.
27. Staniszewska S, Brett J, Mockford C, Barber R. The GRIPP checklist: Strengthening the quality of patient and public involvement reporting in research. International Journal of Technology Assessment in Health Care. 2011;27(4):391-9.
28. Lipscombe LL, Hux JE. Trends in diabetes prevalence, incidence, and mortality in Ontario, Canada 1995–2005: a population-based study. Lancet. 2007;369:750-56.
29. Onat A, Hergenc G, Uyarel H, Can G, Ozhan H. Prevalence, incidence, predictors and outcome of type 2 diabetes in Turkey. Anadolu kardiyoloji dergisi : AKD = the Anatolian journal of cardiology. 2006;6(4):314-21.
30. International Diabetes Federation (IDF). IDF Diabetes Atlas - 7th Edition. 2015 [cited 2017 08 February]; Available from: http://www.diabetesatlas.org/.
31. Lin CC, Li CI, Hsiao CY, Liu CS, Yang SY, Lee CC, et al. Time trend analysis of the prevalence and incidence of diagnosed type 2 diabetes among adults in Taiwan from 2000 to 2007: a population-based study. BMC Public Health. 2013;13(318).
32. Gonzalez ELM, Johansson S, Wallander MA, Rodriguez LAG. Trends in the prevalence and incidence of diabetes in the UK: 1996-2005. J Epidemiol Community Health. 2009;63(4):332-6.
33. Evans JMM, Barnett KN, Ogston SA, Morris AD. Increasing prevalence of type 2 diabetes in a Scottish population: effect of increasing incidence or decreasing mortality? Diabetologia. 2007;50(4):729-32.
34. Young BA, Lin E, Von Korff M, Simon G, Ciechanowski P, Ludman EJ, et al. Diabetes complications severity index and risk of mortality, hospitalization, and healthcare utilization. Am J Manag Care. 2008;14(1):15-24.
35. Runkel M, Muller S, Brydniak R, Runkel N. Downgrading of type 2 diabetes mellitus (T2DM) after obesity surgery: duration and severity matter. Obesity surgery. 2015;25(3):494-9. Epub 2014/09/12.

For peer review only - http://bmjopen.bmj.com/site/about/guidelines.xhtml

36. Okano K, Araki M, Yamamoto M, Ishikawa T, Ichihara K, Yamada O. Exploration of hematological and immunological changes associated with the severity of type 2 diabetes mellitus in Japan. Nursing & health sciences. 2008;10(1):65-9. Epub 2008/02/09.
37. Fram EB, Moazami S, Stern JM. The Effect of Disease Severity on 24-Hour Urine Parameters in Kidney Stone Patients With Type 2 Diabetes. Urology. 2016;87:52-9. Epub 2015/11/04.
38. Eldor R, Klieger Y, Sade-Feldman M, Vaknin I, Varfolomeev I, Fuchs C, et al. CD247, a novel T cell-derived diagnostic and prognostic biomarker for detecting disease progression and severity in patients with type 2 diabetes. Diabetes care. 2015;38(1):113-8. Epub 2014/11/05.
39. Loprinzi PD, Loenneke JP. Evidence of a Link Between Grip Strength and Type 2 Diabetes Prevalence and Severity Among a National Sample of U.S. Adults. Journal of physical activity & health. 2016;13(5):558-61. Epub 2015/11/10.
40. Nahin RL, Byrd-Clark D, Stussman BJ, Kalyanaraman N. Disease severity is associated with the use of complementary medicine to treat or manage type-2 diabetes: data from the 2002 and 2007 National Health Interview Survey. BMC complementary and alternative medicine. 2012;12:193. Epub 2012/10/24.
41. Linzer M, Pierce C, Lincoln E, Miller DR, Payne SM, Clark JA, et al. Preliminary validation of a patient-based self-assessment measure of severity of illness in type 2 diabetes: results from the pilot phase of the Veterans Health Study. The Journal of ambulatory care management. 2005;28(2):167-76. Epub 2005/06/01.
42. Gatlin PK, Insel KC. Severity of Type 2 Diabetes, Cognitive Function, and Self-Care. Biological research for nursing. 2015;17(5):540-8. Epub 2014/11/14.
43. Dunning T, Martin M. Type 2 diabetes: is it serious? Journal of Diabetes Nursing. 1998;2(3):70-6.
44. Windrum P, Garcia-Goni M, Coad H. The Impact of Patient-Centered versus Didactic Education Programs in Chronic Patients by Severity: The Case of Type 2 Diabetes Mellitus. Value in health : the journal of the International Society for Pharmacoeconomics and Outcomes Research. 2016;19(4):353-62. Epub 2016/06/23.
45. Svensson E, Berencsi K, Sander S, Mor A, Rungby J, Nielsen JS, et al. Association of parental history of type 2 diabetes with age, lifestyle, anthropometric factors, and clinical severity at type 2 diabetes diagnosis: results from the DD2 study. Diabetes/metabolism research and reviews. 2016;32(3):308-15. Epub 2015/09/27.
46. Powell DS, Maksoud H, Charge SB, Moffitt JH, Desai M, Da Silva Fihlo RL, et al. Apolipoprotein E genotype, islet amyloid deposition and severity of Type 2 diabetes. Diabetes research and clinical practice. 2003;60(2):105-10. Epub 2003/04/23.
47. Jiang Q, Lyu XM, Yuan Y, Wang L. Plasma miR-21 expression: an indicator for the severity of Type 2 diabetes with diabetic retinopathy. Bioscience reports. 2017;37(2). Epub 2017/01/22.
48. Campbell-Tofte J, Hansen HS, Mu H, Molgaard P. Increased lipids in non-lipogenic tissues are indicators of the severity of type 2 diabetes in mice. Prostaglandins, leukotrienes, and essential fatty acids. 2007;76(1):9-18. Epub 2006/11/14.
49. Garcia-Elorriaga G, Padilla-Reyes M, Cruz-Olivo F, Fuentes-Allen JL, del Rey-Pineda G, Gonzalez-Bonilla C. [Pro-inflammatory cytokines related to severity and mortality in type 2 diabetes patients with soft tissue infection]. Revista medica del Instituto Mexicano del Seguro Social. 2012;50(3):237-41. Epub 2012/11/28. Citocinas, diabetes e infeccion de tejidos blandos. Su relacion con la severidad y mortalidad.
50. Kontopantelis E, Buchan I, Reeves D, Checkland K, Doran T. Relationship between quality of care and choice of clinical computing system: retrospective analysis of family practice performance under the UK's quality and outcomes framework. BMJ open. 2013;3:e003190.

For peer review only - http://bmjopen.bmj.com/site/about/guidelines.xhtml

Table 1 The main (sub) domains identified to quantify the severity of type 2 diabetes

	Severity domain	Severity sub-domain
1.	**Risk factors***	• Duration of type 2 diabetes[40]<br>• Body mass index (BMI)<br>• Hypertension<br>• Hyperlipidaemia<br>• Personal/Family history of cardiovascular disease<br>• Blood glucose levels<br>○ Glycated haemoglobin (HbA_{1c})[35]<br>○ Fasting (FBG) and random (RBG) blood glucose
2.	**Type/pattern of antidiabetic treatment, insulin use and other therapies**	• Antidiabetic therapy ever;[40] Therapies with cardiovascular benefits versus other; Changes in drug treatments;[43] or the number of prescribed treatments[42]<br>• Insulin use: prescription ever or within 1 year of diagnosis; Insulin initiation:[15] time to initiation<br>• Other therapies: angiotensin converting enzyme inhibitors (ACEI) and lipid-regulating therapies
3.	**Microvascular diabetic complications**[15,34]	• Neuropathy (Foot ulcer, Charcot foot, gangrene, amputation)<br>• Nephropathy<br>• Retinopathy (laser therapy and blindness)
4.	**Renal disease**	• Microalbuminuria and proteinuria<br>• Moderate-severe chronic kidney disease (CKD) stages 3 and 4[34]<br>• End-stage renal disease (ESRD): kidney transplant and dialysis
5.	**Cardiovascular and Cerebrovascular disease**	• Atherosclerosis[15,34]<br>• Myocardial infarction (MI) [15,34]<br>• Angina[15,34]<br>• Atrial/ventricular fibrillation (AF)/(VF)[34]<br>• Heart valve disease<br>• Heart failure (HF)[34]<br>• Peripheral vascular disease (PVD)[34]<br>• Transient ischemic attack (TIA)<br>• Ischaemic stroke, haemorrhagic stroke [15,34]
6.	**Cardiovascular and Cerebrovascular interventions**	• Coronary artery bypass graft (CABG)<br>• Coronary artery interventions (PCI/PTCA)<br>• Endovascular aneurysm repair (EVAR)<br>• PVD stenting and bypass procedures<br>• Heart valve interventions<br>• Use of defibrillator<br>• Carotid artery events, stenting and bypass interventions
7.	**Other comorbidities**	• Anxiety<br>• Depression<br>• Dementia<br>• Cognitive impairment
8.	**Hospital admissions**	• Any-cause hospital admissions<br>• Diabetes-attributable admission<br>• Cardiovascular disease-related admission
9.	**Emergency diabetes-related event**	• Hypoglycaemia<br>• Hyperosmolar hyperglycaemic state (HHS)[34]<br>• Diabetic ketoacidosis (DKA) or other coma[34]

**Other demographic data (such as age, gender and the level of deprivation) are important predictors for adverse outcomes and will be included in the later risk-prediction analysis.*

For peer review only - http://bmjopen.bmj.com/site/about/guidelines.xhtml

Figure 1 Severity hierarchy of microvascular diabetic complications

Figure 2 Severity hierarchy of cerebrovascular domains

Figure 3 Severity hierarchy of coronary and vascular domains

Figure 4 Severity hierarchy of cardiovascular disease domains (heart rate, rhythm and conduction)

For peer review only

For peer review only - http://bmjopen.bmj.com/site/about/guidelines.xhtml

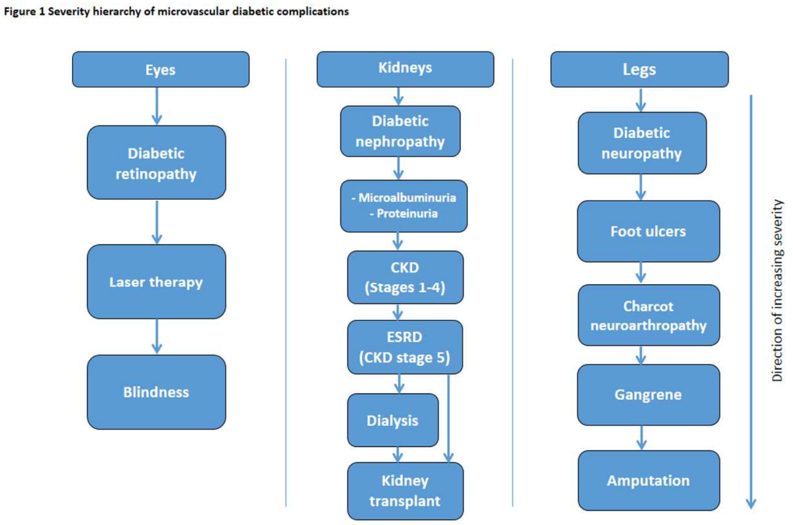

Figure 1 Severity hierarchy of microvascular diabetic complications

338x190mm (300 x 300 DPI)

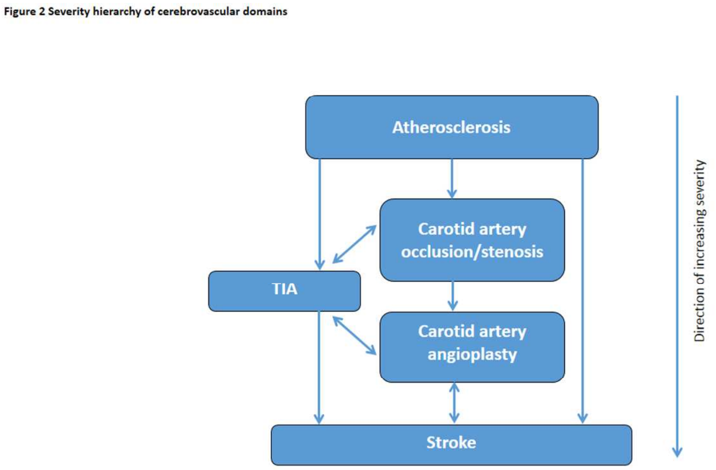

Figure 2 Severity hierarchy of cerebrovascular domains

338x190mm (300 x 300 DPI)

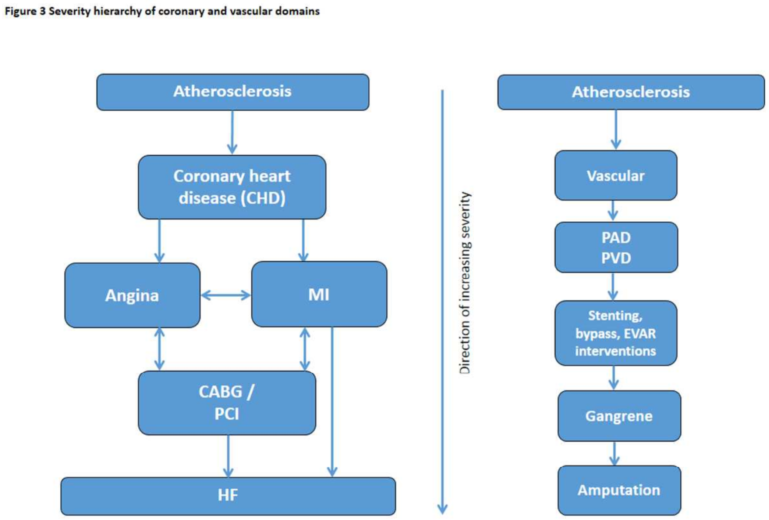

Figure 3 Severity hierarchy of coronary and vascular domains

338x190mm (300 x 300 DPI)

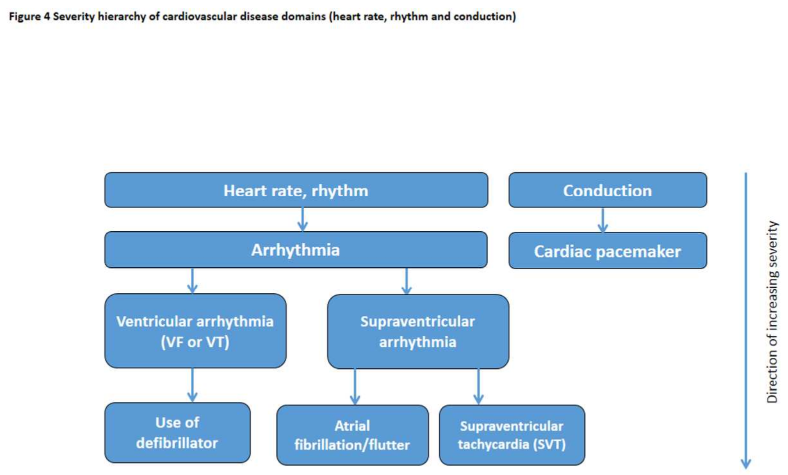

Figure 4 Severity hierarchy of cardiovascular disease domains (heart rate, rhythm and conduction)

338x190mm (300 x 300 DPI)

Supplementary data

Table S1 Inclusion medical codes for type 2 diabetes

	Medical code	Read term
**1.**	506	Non-insulin dependent diabetes mellitus
**2.**	758	Type 2 diabetes mellitus
**3.**	1407	Insulin treated Type 2 diabetes mellitus
**4.**	4513	Non-insulin dependent diabetes mellitus
**5.**	5884	NIDDM - Non-insulin dependent diabetes mellitus
**6.**	8403	Non-insulin dependent diabetes mellitus - poor control
**7.**	12640	Type 2 diabetes mellitus with nephropathy
**8.**	12736	Type 2 diabetes mellitus with gangrene
**9.**	17262	Non-insulin-dependent diabetes mellitus with retinopathy
**10.**	17859	Type 2 diabetes mellitus
**11.**	18143	Type II diabetes mellitus with arthropathy
**12.**	18209	Type 2 diabetes mellitus with renal complications
**13.**	18219	Type II diabetes mellitus
**14.**	18264	Insulin treated Type II diabetes mellitus
**15.**	18278	Insulin treated Type 2 diabetes mellitus
**16.**	18390	Type 2 diabetes mellitus with persistent microalbuminuria
**17.**	18425	Type 2 diabetes mellitus with polyneuropathy
**18.**	18496	Type 2 diabetes mellitus with retinopathy
**19.**	18777	Type 2 diabetes mellitus with renal complications
**20.**	22884	Type II diabetes mellitus
**21.**	24458	Type II diabetes mellitus - poor control
**22.**	24693	Non-insulin dependent diabetes mellitus with arthropathy
**23.**	24836	Type 2 diabetes mellitus with nephropathy
**24.**	25041	Dietary advice for type II diabetes
**25.**	25591	Type 2 diabetes mellitus with exudative maculopathy
**26.**	25627	Type 2 diabetes mellitus - poor control
**27.**	26054	Type 2 diabetes mellitus with persistent proteinuria
**28.**	29979	Non-insulin-dependent diabetes mellitus without complication
**29.**	32627	Type 2 diabetes mellitus with ketoacidosis
**30.**	34268	Type 2 diabetes mellitus with neurological complications
**31.**	34450	Hyperosmolar non-ketotic state in type 2 diabetes mellitus
**32.**	34912	Non-insulin dependent diabetes mellitus with ulcer
**33.**	35385	Type 2 diabetes mellitus with neuropathic arthropathy
**34.**	36633	Hyperosmolar non-ketotic state in type 2 diabetes mellitus
**35.**	37648	Insulin treated non-insulin dependent diabetes mellitus
**36.**	37806	Type 2 diabetes mellitus with peripheral angiopathy
**37.**	40401	Non-insulin dependent diabetes mellitus with gangrene
**38.**	42762	Type 2 diabetes mellitus with retinopathy
**39.**	43227	Type II diabetes mellitus with multiple complications
**40.**	43785	Non-insulin dependent diabetes mellitus with hypoglyca coma
**41.**	44779	Type 2 diabetes mellitus with diabetic cataract
**42.**	44982	Type 2 diabetes mellitus with diabetic cataract
**43.**	45467	Non-insulin dependent diabetes mellitus with polyneuropathy
**44.**	45913	Type 2 diabetes mellitus - poor control
**45.**	45919	Type 2 diabetes mellitus with neurological complications

For peer review only - http://bmjopen.bmj.com/site/about/guidelines.xhtml

Supplementary material to paper "**Using electronic health records to quantify and stratify the severity of type 2 diabetes in primary care in England: rationale and cohort study design**" **Zghebi et al. 2017**

**46.**	46150	Type 2 diabetes mellitus with gangrene
**47.**	46917	Type 2 diabetes mellitus with hypoglycaemic coma
**48.**	47315	Type II diabetes mellitus - poor control
**49.**	47321	Type 2 diabetes mellitus with ophthalmic complications
**50.**	47409	Type II diabetes mellitus with polyneuropathy
**51.**	47816	Type II diabetes mellitus with neuropathic arthropathy
**52.**	47954	Type 2 diabetes mellitus without complication
**53.**	48192	Type II diabetes mellitus with diabetic cataract
**54.**	49074	Type 2 diabetes mellitus with ulcer
**55.**	49655	Type II diabetes mellitus with retinopathy
**56.**	49869	Type 2 diabetes mellitus with arthropathy
**57.**	50225	Type II diabetes mellitus with renal complications
**58.**	50429	Non-insulin-dependent diabetes mellitus with ophthalm comps
**59.**	50527	Type II diabetes mellitus with polyneuropathy
**60.**	50609	Pre-existing diabetes mellitus, non-insulin-dependent
**61.**	50813	Type II diabetes mellitus with mononeuropathy
**62.**	51756	Type 2 diabetes mellitus with ketoacidotic coma
**63.**	52303	Non-insulin-dependent diabetes mellitus with renal comps
**64.**	53392	Type II diabetes mellitus without complication
**65.**	54899	Type II diabetes mellitus with peripheral angiopathy
**66.**	55075	Type II diabetes mellitus with ulcer
**67.**	55842	Non-insulin-dependent diabetes mellitus with neuro comps
**68.**	56268	Type II diabetes mellitus with hypoglycaemic coma
**69.**	57278	Type II diabetes mellitus with renal complications
**70.**	58604	Type II diabetes mellitus with retinopathy
**71.**	59253	Type 2 diabetes mellitus with arthropathy
**72.**	59365	Non-insulin dependent diabetes mellitus with nephropathy
**73.**	59725	Type II diabetes mellitus with ophthalmic complications
**74.**	60699	Type 2 diabetes mellitus with peripheral angiopathy
**75.**	60796	Type II diabetes mellitus with persistent proteinuria
**76.**	61071	Type 2 diabetes mellitus with hypoglycaemic coma
**77.**	62107	Type II diabetes mellitus with gangrene
**78.**	62146	Non-insulin-dependent diabetes mellitus with multiple comps
**79.**	62674	Type 2 diabetes mellitus with mononeuropathy
**80.**	63690	Type 2 diabetes mellitus with gastroparesis
**81.**	64571	Type II diabetes mellitus with nephropathy
**82.**	64668	Insulin treated Type II diabetes mellitus
**83.**	65267	Type 2 diabetes mellitus with multiple complications
**84.**	65704	Type 2 diabetes mellitus with ulcer
**85.**	66965	Type 2 diabetes mellitus with neuropathic arthropathy
**86.**	67905	Type II diabetes mellitus with neurological complications
**87.**	69278	Non-insulin depend diabetes mellitus with diabetic cataract
**88.**	70316	Type 2 diabetes mellitus with ophthalmic complications
**89.**	72320	Non-insulin dependent diabetes mellitus with mononeuropathy
**90.**	83532	Diabetes type 2 review
**91.**	85991	Type II diabetes mellitus with persistent microalbuminuria
**92.**	91646	Type II diabetes mellitus with ulcer
**93.**	93727	Type II diabetes mellitus with diabetic cataract
**94.**	95351	Type II diabetes mellitus with mononeuropathy
**95.**	98616	Type II diabetes mellitus with neurological complications
**96.**	98723	Type II diabetes mellitus with hypoglycaemic coma
**97.**	100964	Type II diabetes mellitus with ophthalmic complications
**98.**	101801	Type II diabetic dietary review
**99.**	102201	Type II diabetes mellitus with nephropathy

For peer review only - http://bmjopen.bmj.com/site/about/guidelines.xhtml

**100.**	102611	Type 2 diabetic dietary review
**101.**	103902	Type II diabetes mellitus with arthropathy
**102.**	104323	Type II diabetes mellitus with gangrene
**103.**	104639	Type II diabetes mellitus with peripheral angiopathy
**104.**	105784	Type 2 diabetes mellitus without complication
**105.**	106061	Type II diabetes mellitus with ketoacidotic coma
**106.**	106528	Type II diabetes mellitus with ketoacidosis
**107.**	107701	Hyperosmolar non-ketotic state in type II diabetes mellitus
**108.**	107824	Type II diabetes mellitus in remission
**109.**	108005	Type 2 diabetes mellitus with multiple complications
**110.**	109103	Type II diabetes mellitus without complication
**111.**	109197	Type II diabetes mellitus with neuropathic arthropathy
**112.**	109865	Type 2 diabetes mellitus with polyneuropathy

For peer review only

Table S2 Exclusion type 1 diabetes codes

	Medical code	Read term
**1.**	1038	Insulin dependent diabetes mellitus
**2.**	1549	Type 1 diabetes mellitus
**3.**	1647	Insulin dependent diabetes mellitus
**4.**	6509	Insulin dependent diabetes mellitus with retinopathy
**5.**	6791	Insulin dependent diabetes mellitus - poor control
**6.**	10418	Type 1 diabetes mellitus with nephropathy
**7.**	10692	Type 1 diabetes mellitus with ketoacidosis
**8.**	12455	Type I diabetes mellitus
**9.**	17545	Type I diabetes mellitus with diabetic cataract
**10.**	17858	Type 1 diabetes mellitus
**11.**	18230	Type 1 diabetes mellitus with neuropathic arthropathy
**12.**	18387	Type 1 diabetes mellitus with retinopathy
**13.**	18505	IDDM-Insulin dependent diabetes mellitus
**14.**	18642	Type 1 diabetes mellitus with arthropathy
**15.**	18683	Type 1 diabetes mellitus with ulcer
**16.**	21983	Type 1 diabetes mellitus with renal complications
**17.**	22871	Type 1 diabetes mellitus with exudative maculopathy
**18.**	24423	Type I diabetes mellitus
**19.**	24490	Diabetes mellitus, juvenile type, no mention of complication
**20.**	24694	Insulin dependent diabetes mellitus with mononeuropathy
**21.**	26855	Unstable insulin dependent diabetes mellitus
**22.**	30294	Type 1 diabetes mellitus with persistent microalbuminuria
**23.**	30323	Type 1 diabetes mellitus with persistent proteinuria
**24.**	31310	Insulin dependent diabetes maturity onset
**25.**	32359	Perceived control of insulin-dependent diabetes
**26.**	35288	Type 1 diabetes mellitus - poor control
**27.**	38161	Type I diabetes mellitus with retinopathy
**28.**	39070	Type 1 diabetes mellitus with hypoglycaemic coma
**29.**	39809	Insulin dependent diab mell with neuropathic arthropathy
**30.**	40023	Diabetes mellitus, juvenile type, with hyperosmolar coma
**31.**	40682	Type 1 diabetes mellitus maturity onset
**32.**	40837	Type 1 diabetes mellitus with ketoacidotic coma
**33.**	41049	Type 1 diabetes mellitus with retinopathy
**34.**	41716	Insulin dependent diabetes mellitus with polyneuropathy
**35.**	42567	Diabetes mellitus, juvenile type, with ketoacidotic coma
**36.**	42729	Type I diabetes mellitus with hypoglycaemic coma
**37.**	42831	Type 1 diabetes mellitus with neurological complications
**38.**	43921	Unstable type 1 diabetes mellitus
**39.**	44260	Insulin dependent diabetes mellitus with diabetic cataract
**40.**	44440	Insulin dependent diabetes mellitus with hypoglycaemic coma
**41.**	44443	Insulin dependent diabetes mellitus with ulcer
**42.**	45276	Insulin dependent diabetes mellitus with multiple complicat
**43.**	45914	Type 1 diabetes mellitus - poor control
**44.**	46301	Type 1 diabetes mellitus with polyneuropathy
**45.**	46850	Type I diabetes mellitus - poor control
**46.**	46963	Insulin-dependent diabetes mellitus with renal complications

For peer review only - http://bmjopen.bmj.com/site/about/guidelines.xhtml

**47.**	47582	Type 1 diabetes mellitus with renal complications
**48.**	47649	Type 1 diabetes mellitus with ophthalmic complications
**49.**	47650	Type 1 diabetes mellitus with multiple complications
**50.**	49146	Type I diabetes mellitus with neurological complications
**51.**	49276	Insulin-dependent diabetes mellitus with ophthalmic comps
**52.**	49554	Type 1 diabetes mellitus with diabetic cataract
**53.**	49949	Unstable type I diabetes mellitus
**54.**	50960	Pre-existing diabetes mellitus, insulin-dependent
**55.**	51261	Insulin dependent diabetes mellitus
**56.**	51957	Type I diabetes mellitus with ulcer
**57.**	52104	Insulin dependent diabetes mellitus with multiple complicatn
**58.**	52283	Insulin-dependent diabetes mellitus with neurological comps
**59.**	53200	Diabetes mellitus, juvenile type, with ketoacidosis
**60.**	54008	Type 1 diabetes mellitus with neuropathic arthropathy
**61.**	54600	Unstable insulin dependent diabetes mellitus
**62.**	55239	Type 1 diabetes mellitus with gastroparesis
**63.**	56448	Insulin-dependent diabetes without complication
**64.**	57621	Insulin dependent diabetes mellitus with nephropathy
**65.**	60107	Unstable type I diabetes mellitus
**66.**	60208	Type I diabetes mellitus with neuropathic arthropathy
**67.**	60499	Insulin dependent diabetes mellitus with gangrene
**68.**	61344	Type I diabetes mellitus with renal complications
**69.**	61829	Type 1 diabetes mellitus with neurological complications
**70.**	62209	Type I diabetes mellitus with ketoacidosis
**71.**	62352	Type I diabetes mellitus with arthropathy
**72.**	62613	Type I diabetes mellitus without complication
**73.**	63017	Type I diabetes mellitus maturity onset
**74.**	64446	Insulin dependent diab mell with peripheral angiopathy
**75.**	65616	Insulin dependent diabetes mellitus with arthropathy
**76.**	66145	Type I diabetes mellitus with ketoacidotic coma
**77.**	66872	Type I diabetes mellitus with nephropathy
**78.**	68105	Type 1 diabetes mellitus with mononeuropathy
**79.**	68390	Type 1 diabetes mellitus with ulcer
**80.**	68792	Diabetes mellitus, juvenile type, + unspecified complication
**81.**	69043	Dietary advice for type I diabetes
**82.**	69676	Type 1 diabetes mellitus without complication
**83.**	69748	Diabetes mellitus, juvenile type, + ophthalmic manifestation
**84.**	69993	Type 1 diabetes mellitus with gangrene
**85.**	70766	Type 1 diabetes mellitus with hypoglycaemic coma
**86.**	72702	Insulin dependent diabetes mellitus - poor control
**87.**	85660	Diabetes type 1 review
**88.**	91942	Type I diabetes mellitus with multiple complications
**89.**	91943	Type I diabetes mellitus with polyneuropathy
**90.**	93468	Type 1 diabetes mellitus with peripheral angiopathy
**91.**	93875	Insulin dependent diabetes mellitus with retinopathy
**92.**	93878	Type I diabetes mellitus with ulcer
**93.**	93922	Diabetes mellitus, juvenile type, with renal manifestation
**94.**	95343	Type I diabetes mellitus with retinopathy
**95.**	95992	Type I diabetes mellitus without complication
**96.**	96235	Type I diabetes mellitus maturity onset

For peer review only - http://bmjopen.bmj.com/site/about/guidelines.xhtml

**97.**	97446	Type 1 diabetes mellitus maturity onset
**98.**	97474	Unstable type 1 diabetes mellitus
**99.**	97849	Insulin dependent diabetes maturity onset
**100.**	97894	Type I diabetes mellitus with exudative maculopathy
**101.**	98071	Insulin-dependent diabetes mellitus with ophthalmic comps
**102.**	98392	Maturity onset diabetes in youth type 1
**103.**	98704	Insulin dependent diabetes mellitus with ulcer
**104.**	99231	Type I diabetes mellitus with mononeuropathy
**105.**	99311	Type I diabetes mellitus with ophthalmic complications
**106.**	99716	Insulin dependent diabetes mellitus with hypoglycaemic coma
**107.**	99719	Insulin-dependent diabetes without complication
**108.**	100770	Insulin dependent diabetes mellitus with diabetic cataract
**109.**	101311	Insulin dependent diabetes mellitus with polyneuropathy
**110.**	101735	Insulin-dependent diabetes mellitus with neurological comps
**111.**	102112	Type I diabetes mellitus with gangrene
**112.**	102163	Insulin dependent diabetes mellitus with nephropathy
**113.**	102620	Type I diabetes mellitus with persistent microalbuminuria
**114.**	102704	Type I diabetic dietary review
**115.**	102740	Type 1 diabetes mellitus with ophthalmic complications
**116.**	102946	Insulin-dependent diabetes mellitus with renal complications
**117.**	104453	Type 1 diabetic dietary review
**118.**	105337	Type I diabetes mellitus - poor control
**119.**	108007	Type I diabetes mellitus with multiple complications
**120.**	108360	Type I diabetes mellitus in remission
**121.**	108724	Type I diabetes mellitus with gastroparesis
**122.**	109051	Insulin dependent diabetes mellitus with gangrene
**123.**	109628	Type 1 diabetes mellitus in remission
**124.**	109837	Type I diabetes mellitus with renal complications
**125.**	109878	Diet advice for insulin-dependent diabetes
**126.**	110400	Type 1 diabetes mellitus with diabetic cataract
**127.**	111106	Type 1 diabetes mellitus without complication

For peer review only

Supplementary material to paper "**Using electronic health records to quantify and stratify the severity of type 2 diabetes in primary care in England: rationale and cohort study design" Zghebi et al. 2017**

Figure S1 Severity hierarchy of cardiovascular disease domains (cardiac muscle- myocardium and pericardium).

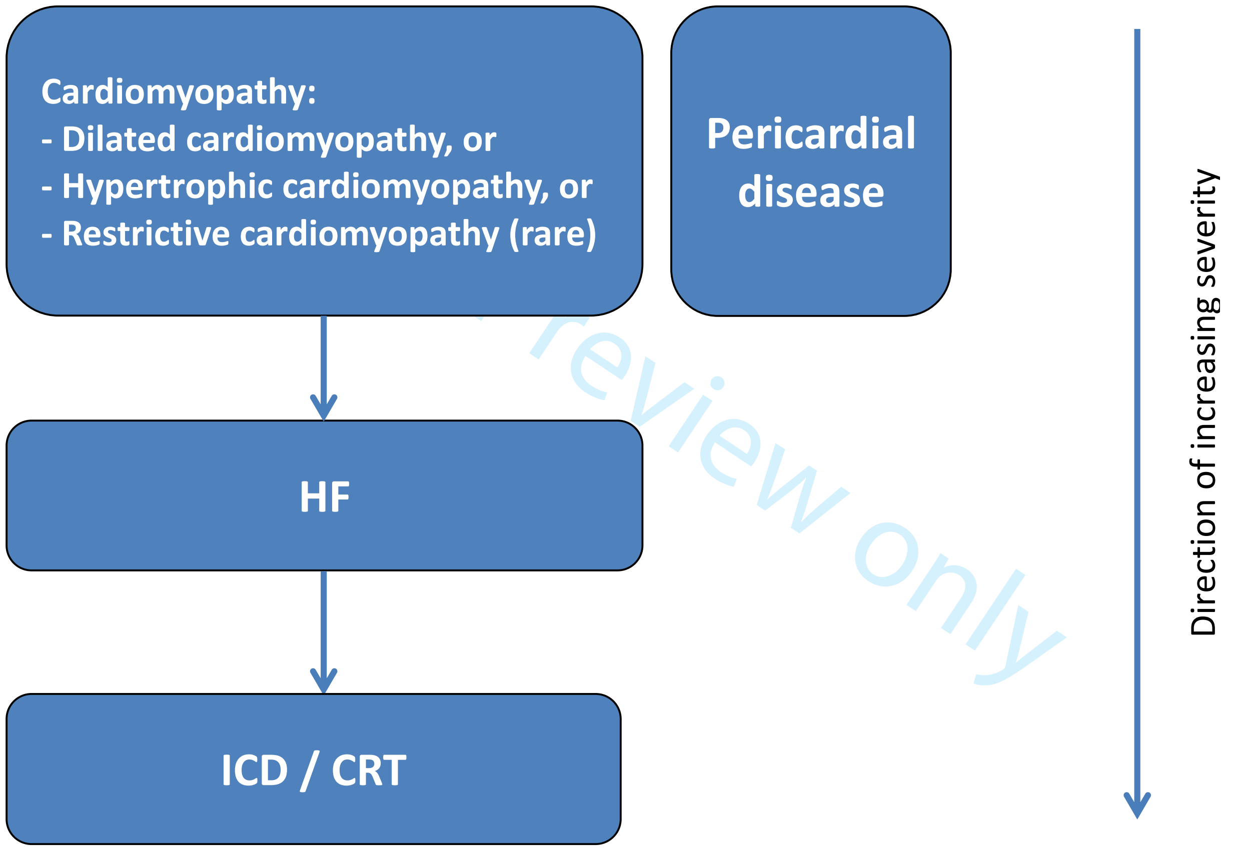

For peer review only - http://bmjopen.bmj.com/site/about/guidelines.xhtml

Supplementary material to paper "**Using electronic health records to quantify and stratify the severity of type 2 diabetes in primary care in England: rationale and cohort study design" Zghebi et al. 2017**

Figure S2 Severity hierarchy of cardiovascular disease domains (Heart valve diseases)

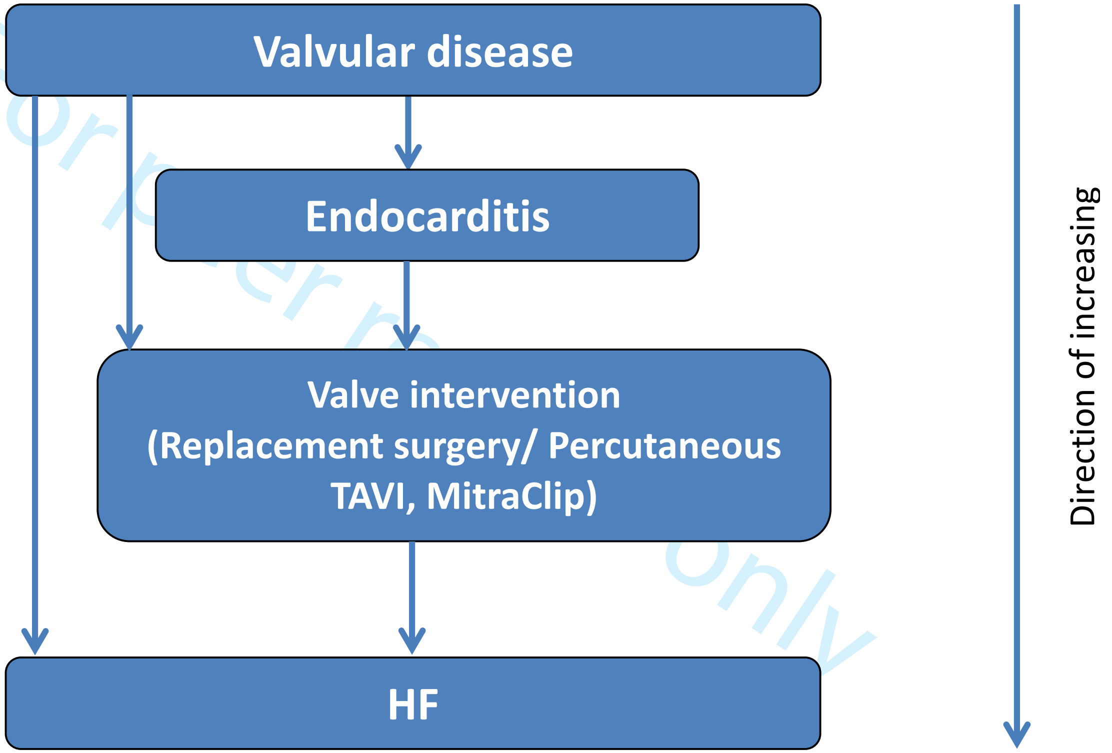

For peer review only - http://bmjopen.bmj.com/site/about/guidelines.xhtml

Supplementary material to paper "**Using electronic health records to quantify and stratify the severity of type 2 diabetes in primary care in England: rationale and cohort study design" Zghebi et al. 2017**

Table S3 Factors used in previous studies to define severity of diabetes

Study ID	Factors used to define diabetes severity / Outcome(s)	Severity scale/ levels	Validation study
**Gini et al. (2016)[(1)]**	• Indication for insulin therapy<br>• The presence of diabetes-related complications<br><br>**Outcomes:**<br>Assessing the positive predictive value (PPV) for the presence/absence of type 2 diabetes against the GP's response to the questionnaire and measure the agreement on the severity level.	Four levels of T2DM severity:<br>• **Level 1**: Clinical definition of the disease, no indication for Insulin therapy and no complications listed in Level 3.<br>• **Level 2**: as in Level 1 except insulin is indicated.<br>• **Level 3**: as in Level 1 except with one of the following complications: (1) arterial stenosis, angina, MI, TIA, ischaemic stroke, intermittent claudication, diabetic foot ulcer, lower limb amputation, (2) retinopathy, (3) incipient or overt diabetic nephropathy/ dialysis.<br>• **Level 4**: As in Level 3 except insulin is indicated	A random sample of identified cases was validated by interviewing their self-selected general practitioners (GPs).
**Grootenhuis et al. (1994)[(2)]**	Type 2 diabetes symptom severity items categorised over six clinical dimensions:<br>• Hyperglycaemic<br>• Hypoglycaemic<br>• Psychological<br>• Cardiovascular<br>• Neuropathic<br>• Ophthalmological<br><br>**Outcomes:**<br>To measure differences in symptom severity between patients and detect changes over time within patients.	A 34-item patient-derived checklist used as a measure for type 2 diabetes symptom severity.	The face and content validity of severity items and dimension structures were based on literature and experiences of diabetologists.
**Young et al. (2008)[(3)]**	From laboratory data and diabetes-related complications:<br>• Retinopathy<br>• Nephropathy<br>• Neuropathy<br>• Cerebrovascular<br>• Cardiovascular<br>• Peripheral vascular disease<br>• Metabolic<br><br>**Outcomes:**<br>Risk of mortality, hospitalisation and healthcare utilisation.	Developed the Diabetes Complication Severity Index (DCSI) that is categorised to two or three levels (0=no abnormality; 1=some abnormality, 2=severe abnormality) depending on the severity of the complication. So if no abnormalities were present, the patient received no score, a 1 was added to the DCSI if patient had any complication classified as some abnormality and 2 was added if patient had any complication classified as severe abnormality.	The study design was a validation sample

For peer review only - http://bmjopen.bmj.com/site/about/guidelines.xhtml

Supplementary material to paper "**Using electronic health records to quantify and stratify the severity of type 2 diabetes in primary care in England: rationale and cohort study design**" **Zghebi et al. 2017**

References

1. Gini R, Schuemie MJ, Mazzaglia G, Lapi F, Francesconi P, Pasqua A, et al. Automatic identification of type 2 diabetes, hypertension, ischaemic heart disease, heart failure and their levels of severity from Italian General Practitioners' electronic medical records: a validation study. BMJ open. 2016;6(12):e012413. Epub 2016/12/13.
2. Grootenhuis PA, Snoek FJ, Heine RJ, Bouter LM. Development of a type 2 diabetes symptom checklist: a measure of symptom severity. Diabetic medicine : a journal of the British Diabetic Association. 1994;11(3):253-61. Epub 1994/04/01.
3. Young BA, Lin E, Von Korff M, Simon G, Ciechanowski P, Ludman EJ, et al. Diabetes complications severity index and risk of mortality, hospitalization, and healthcare utilization. Am J Manag Care. 2008;14(1):15-24.

For peer review only

For peer review only - http://bmjopen.bmj.com/site/about/guidelines.xhtml
